# Modeling impact and cost‐effectiveness of driving‐Y gene drives for malaria elimination in the Democratic Republic of the Congo

**DOI:** 10.1111/eva.13331

**Published:** 2022-01-07

**Authors:** Nawaphan Metchanun, Christian Borgemeister, Gaston Amzati, Joachim von Braun, Milen Nikolov, Prashanth Selvaraj, Jaline Gerardin

**Affiliations:** ^1^ Center for Development Research (ZEF) University of Bonn Bonn Germany; ^2^ Université Evangélique en Afrique Bukavu Democratic Republic of the Congo; ^3^ Institute for Disease Modeling Bellevue Washington USA; ^4^ Department of Preventive Medicine and Institute for Global Health Northwestern University Chicago Illinois USA

**Keywords:** Africa, biotechnology, gene drive, genetically modified organisms, high burden countries, malaria, malaria elimination, sex ratio distorter

## Abstract

Malaria elimination will be challenging in countries that currently continue to bear high malaria burden. Sex‐ratio‐distorting gene drives, such as driving‐Y, could play a role in an integrated elimination strategy if they can effectively suppress vector populations. Using a spatially explicit, agent‐based model of malaria transmission in eight provinces spanning the range of transmission intensities across the Democratic Republic of the Congo, we predict the impact and cost‐effectiveness of integrating driving‐Y gene drive mosquitoes in malaria elimination strategies that include existing interventions such as insecticide‐treated nets and case management of symptomatic malaria. Gene drive mosquitoes could eliminate malaria and were the most cost‐effective intervention overall if the drive component was highly effective with at least 95% X‐shredder efficiency at relatively low fertility cost, and associated cost of deployment below 7.17 $int per person per year. Suppression gene drive could be a cost‐effective supplemental intervention for malaria elimination, but tight constraints on drive effectiveness and cost ceilings may limit its feasibility.

## 1 INTRODUCTION

Female *Anopheles* mosquitoes can transmit *Plasmodium* parasites that cause malaria, a life‐threatening infectious disease. The most commonly used vector control methods to prevent mosquito bites are sleeping under insecticide‐treated mosquito nets (ITNs) and spraying the inside walls of a house with an insecticide (indoor residual spraying, IRS) (WHO, 2019b). Treatment of symptomatic malaria cases with artemisinin‐based combination therapy (ACT) can effectively manage malaria burden, although access to prompt and quality care remains a barrier. Nevertheless, despite being preventable and treatable, with considerable control successes during the last 20 years (WHO, 2016a, 2019a), malaria still has devastating impacts on the health and livelihoods of people around the world. The World Health Organization (WHO) estimates that about 3.7 billion people are at risk of the disease in 97 predominantly tropical countries (UNICEF, 2019; WHO, 2019a), even though billions of dollars are spent annually on malaria control and elimination. Most malaria cases occur in sub‐Saharan Africa (SSA), accounting for 93% of total malaria cases worldwide (WHO, 2019a, 2020c). With 12% of all cases in SSA, the Democratic Republic of the Congo (DRC) is the second highest‐burden country on the continent (WHO, 2019a). Nearly all of the DRC’s population lives in high malaria transmission zones (Vector Link, 2019). Consequently, the disease remains one of the country's most serious public health problems and is the number one cause of death (IHME, 2018; Ngatu et al., 2019).

Despite sustained malaria control, malaria incidence in the DRC has increased in the last few years (WHO, 2019a), and more than 40% of children who fell ill because of malaria did not receive adequate care (Unitaid, 2019; WHO, 2019a). Health system weaknesses and gaps in the coverage of core interventions caused by financial and programmatic limitations are likely responsible for this recent rise in cases (WHO Malaria Policy Advisory Committee 2018), and elimination remains elusive. Sustained access to vector control has been a central strategy in the DRC’s complex operating environment, where challenges are compounded by domestic political conflicts (Ngatu et al., 2019) and insufficient funding for malaria control (Head et al., 2017). These challenges emphasize the urgent necessity of developing new strategies for malaria control and elimination for the DRC and beyond (WHO Malaria Policy Advisory Committee, 2018; President’s Malaria Initiative, 2019; Roll Back Malaria Partnership, 2017; WHO, 2019a).

Transgenic mosquitoes carrying gene drives have recently been successfully developed in the laboratory (Committee on Gene Drive Research in Non‐Human Organisms, 2016). Gene drive is a novel method that involves the inheritance of specific traits from one generation to the next at rates higher than the 50% chance afforded through Mendelian inheritance in heterozygotes, and gives certain genes a substantially higher or lower probability of inheritance and thereby alters the frequency of such genes in the population. A gene that alters the fertility or survival of the target species could thereby alter the species population size, depending on the species and the drive system applied (Beaghton et al., 2017; Buchman et al., 2018; Burt & Deredec, 2018; Gantz et al., 2015; Hammond & Galizi, 2017; Marshall et al., 2011; North et al., 2019, 2020). Given rising resistance to existing insecticides and antimalarial drugs (Bhagavathula et al., 2016; Bull et al., 2019; Mnzava et al., 2015; Protopopoff et al., 2018; WHO, 2014), gene drive mosquitoes might hold great potential to accelerate and achieve lasting gains in malaria control (Committee on Gene Drive Research in Non‐Human Organisms, 2016). The future utility of gene drives also depends on their economic aspects compared with existing or future alternatives (WHO/TDR & FNIH, 2014). This study assesses the cost‐effectiveness of gene drives together with conventional interventions by estimating Disability‐Adjusted Life Years (DALYs), DALYs averted, and the cost‐effectiveness of vector control methods in the DRC.

Although gene drive has yet to pass the research and development stage, with driving‐Y gene drive yet to be developed in the laboratory and lead candidates of gene drives only tested in confined cage trials (ENSSER, 2019; Simoni et al., 2020), public concern has been voiced over gene‐related technologies that intend to alter the targeted species population, including previous techniques such as *Wolbachia*‐based and sterile insect techniques. For example, concerns on previously developed genetic controls, such as a genetically modified version of *Aedes aegypti* for control of mosquito‐transmitted arboviral diseases, have led to a debate on potential hazards including the unexpected contamination of transgenes in the environment, possible harms to the targeted species’ morphology, consequences of transgenes to gene flows (Paes de Andrade et al., 2016), and whether releasing modified mosquitoes to control vector‐borne diseases is suitable for a large‐scale implementation (Flores & O’Neill, 2018). These questions remain for gene drives. At the same time, proof of efficacy presents a challenge, and informed decision‐making on gene drive releases into the wild will require a step‐wise approach for safety monitoring, additional information about potential effectiveness, and the evaluation of potential environmental risk and benefits to health in terms of disease control (Committee on Gene Drive Research in Non‐Human Organisms, 2016; WHO, 2010).

Disease modeling is a powerful tool that can complement laboratory findings and help develop control strategies involving transgenic mosquitoes. The scientific community, including the WHO and other policy groups, has increasingly recognized the importance of disease modeling in guiding the development of gene drives and genetically modified organisms (Committee on Gene Drive Research in Non‐Human Organisms, 2016; James et al., 2018; WHO, 2010). In this work, we explore the possible outcomes of applying gene drives as an intervention for malaria control in SSA settings in combination with established control programs—including ITNs and ACT distributions—while also evaluating the economic cost of the resulting programs.

We model areas in eight provinces of the DRC by calibrating the transmission intensity of the selected areas to malaria prevalence estimates from open data sources, accounting for existing intervention coverage, and using local rainfall and temperature to drive seasonality in vector abundance. In each selected province, we determine effective release strategies of gene drive mosquitoes and define parameter regimes of a sex‐ratio‐distorting suppressive gene drive system, the driving‐Y system, that results in the elimination of malaria. In the driving‐Y system, the process of shredding the male's X chromosome results in male‐biased progeny as the Y chromosome can still be carried through unaffected sperm and driven into the next generation (Hammond & Galizi, 2017). The system leads to fecundity reduction, the reduction of the potential to produce offspring, which affects the egg batch size and has implications for the success of the driving system (Bradshaw & McMahon, 2008; Moro et al., 2018). We simulate various intervention scenarios, including both conventional and gene drive approaches to vector control, identify combinations of interventions that lead to malaria elimination, and use modeled predictions of malaria burden to estimate DALYs averted and compare the cost‐effectiveness of driving‐Y gene drives and existing vector control interventions in the DRC.

## 2 MATERIALS AND METHODS

The simulations in this study use Epidemiological MODeling software (EMOD) v2.18 (IDM, 2019), an agent‐based, discrete‐time, Monte Carlo simulator of malaria transmission with a vector life cycle (Eckhoff, 2011) and within‐host parasite and immune dynamics (Eckhoff, 2012, 2013). The modeling framework combines an epidemiological model of *Plasmodium falciparum* transmission between individual human agents and cohorts of mosquito agents distinguished by life stage, feeding and oviposition stage, age, and genotype. The vector lifecycle consists of four stages: egg, larvae, immature adults, and host‐seeking adults, with temperature‐dependent larval development, immature maturation, and sporogony. Mosquito abundance is driven by the availability of larval habitat, and mosquito mortality is also affected by temperature and humidity. In humans, the model includes asexual parasite and gametocyte densities, human immunity, effects of antimalarial drugs, and symptomatic aspects of malaria, all of which have been previously calibrated to field data (Gerardin, Eckhoff, et al., 2015; Gerardin, Ouedraogo, et al., 2015; Selvaraj et al., 2018). The model dynamically simulates vector‐human and human‐vector transmissions during blood meals. Driving‐Y is one of several gene drive strategies that can be simulated within EMOD (Selvaraj et al., 2020).

**FIGURE 1:**
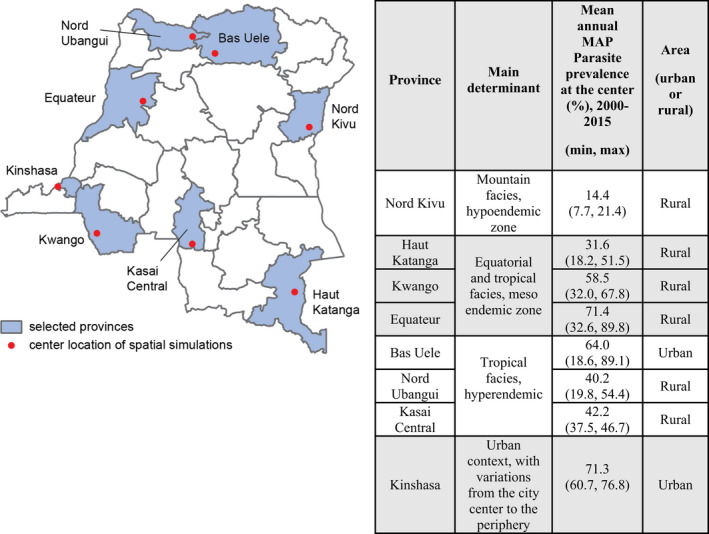
Location and epidemiological characteristics of the eight modeled sites in the DRC. Map: Geolocation of the central nodes of the 25 x 25 km simulation areas. Table: Main determinants, rural/urban classification, and MAP estimates of the 2015 parasite prevalence for each site

We selected eight provinces in the DRC for simulations (Figure 1) in both nonspatial and spatial simulation frameworks. The selection was based on malaria parasite prevalence data from the DRC‐Demographic and Health Survey (DHS) 2013–14 (USAID, 2015a, 2015b), Malaria Atlas Project (MAP) parasite prevalence estimates (The Malaria Atlas Project, 2018), and provincial stratification by climate zones, endemicity, and urban/rural (President’s Malaria Initiative, 2019) to ensure that selected locations spanned the range of transmission intensities observed in the DRC (details of site selection are in Supporting information 1). For each site, simulations were run on a square 25km x 25km grid containing 25 nodes, 5 kilometers apart in both the nonspatial framework where no vector migration was present across all nodes and the spatial framework where vector migration was included. The model's outputs of malaria incidence and mortality were then used to assess the cost‐effectiveness of interventions.

We selected a central node for each simulated province by identifying a survey point from the MAP parasite prevalence survey database (The Malaria Atlas Project, 2018) such that the 25‐node simulation area fell entirely within the selected province (QGIS Development Team, 2019) and used WorldPop population estimates (School of Geography & Environmental Science, Southampton University of Democratic Republic of the Congo 100m Population, 2013) to verify that the central node and all surrounding nodes are populated. We applied site‐specific environmental covariates based on node geolocation including climate (rainfall, temperature, humidity) and seasonality averaged from monthly vectorial capacity. Since *An*. *gambiae* mosquitoes, the only modeled mosquito species in this study, breed primarily in temporary puddles replenished by rainfall and drained through evaporation and infiltration (Koenraadt et al., 2004), the simulations used climate data to model the availability of larval habitat, which drove the number of vectors throughout the year and thus biting intensity and transmission (Eckhoff, 2011). Weather stations and readings by the National Oceanic and Atmospheric Administration (NOAA) Global Surface Summary of the Day were used for generating temperature and dewpoint anomalies. Baseline monthly averages were generated using WorldClim 1.4 raster files in a grid format, 2.5 arc minutes, and 30 arc seconds from WorldClim 1.4 (Hijmans et al., 2005). Rainfall files were generated by downscaling RFE 2.0 Rainfall Estimates from NOAA's Climate Prediction Center (NOAA, 2006). In both nonspatial and spatial simulation frameworks, seasonality was enforced in the models. To calibrate seasonality of larval habitat abundance, we simulated 100 samples per iteration and 10 iterations per site and minimized the Euclidean distance between simulated monthly vectorial capacity and the average monthly vectorial capacity between 2000 and 2015 in two public datasets (v200906 and Sheffield) (IRI/LDEO, 2019). Daily temperature series was generated for each node as in Chabot‐Couture et al. (2014).

We calibrated the overall larval habitat abundance (25 samples per iteration, 3 iterations per site) by sampling global scale factors on the previously fitted seasonality profile to minimize the Euclidean distance between the model's parasite prevalence and the mean 2015 annual parasite prevalence of the location from MAP estimates(Bhatt et al., 2015). The annual means of estimated parasite rate in children between the ages of two and ten (PfPR_2‐10_) from the year 2000 to 2015 were retrieved from MAP rasters (Bhatt et al., 2015) for all simulation nodes. For Haut Katanga, where parasite prevalence was lower, the larval habitat multiplier was calibrated so that the average modeled parasite prevalence for years 2013–2015 was close to the MAP estimates for the same period. We set each node's population to 1000 individuals and set birth and mortality rates to 36.3 per 1000 people per year. The human population size used is large enough to sustain low‐transmission malaria but not unrealistically large for rural areas. The simulation was run for 50 years to initialize population immunity.

In the final 10 years of the 50‐year initialization period, the following interventions were imposed (President’s Malaria Initiative, 2019):

ITNs: modeled ITN usage was based on % of children under the age of five (<5) who slept under an ITN the previous night: 6%, 38%, and 56% in 2007, 2010, and 2013, respectively, and applied to individuals of all ages.
Case management of symptomatic cases with artemisinin‐based combination therapy (ACTs): 19% of uncomplicated malaria cases in all ages received treatment with artemether–lumefantrine, based on the 2013 DHS survey reporting 19% of febrile children under 5 receiving ACT.
Indoor Residual Spraying (IRS) was not included as less than 1% of the DRC population was protected by IRS between 2007 and 2018 (WHO, 2010, 2019a).

After the 50‐year initialization, simulations were run for the next 15 years, covering the period of 2016–2030 to align with WHO’s global technical strategy for all malaria‐endemic countries to attain malaria control and elimination by the year 2030 (WHO, 2015a). Outcomes were evaluated at 5, 10, and 15 years.

For ITNs used in the model, the initial strength of the blocking effect on indoor mosquito feeding on an individual with an ITN was 0.9, and the blocking decayed at an exponential rate with a mean of 730 days. The blocking effect captures the physical barrier of a bednet that prevents a mosquito from making contact with a human. The initial strength of the killing effect was 0.6 and decayed at an exponential rate with a mean of 1460 days. Killing and blocking parameters were obtained from calibration to clinical trial data (Eckhoff, 2013). The model assumed an individual who received an ITN had a 0.65 probability of using it on any given night, and ITNs were redistributed every 3 years. For ACT, the parameters and values used in the model followed (Gerardin, Eckhoff, et al., 2015).

The model focuses on final mosquito offspring under the gene drive intervention, and females that mate with a male carrying driving‐Y will have as offspring wild‐type females and males carrying the driving‐Y. The fraction of offspring that are driving‐Y males is then 0.5+0.5*(X‐shredder efficiency), and the fraction of offspring that are females is 0.5–0.5*(X‐shredder efficiency). Only females that mate with a driving‐Y male have their fertility reduced, and the total egg batch size is reduced by the fecundity reduction for each female that mates with a modified male (Eckhoff et al., 2017).

**FIGURE 2:**
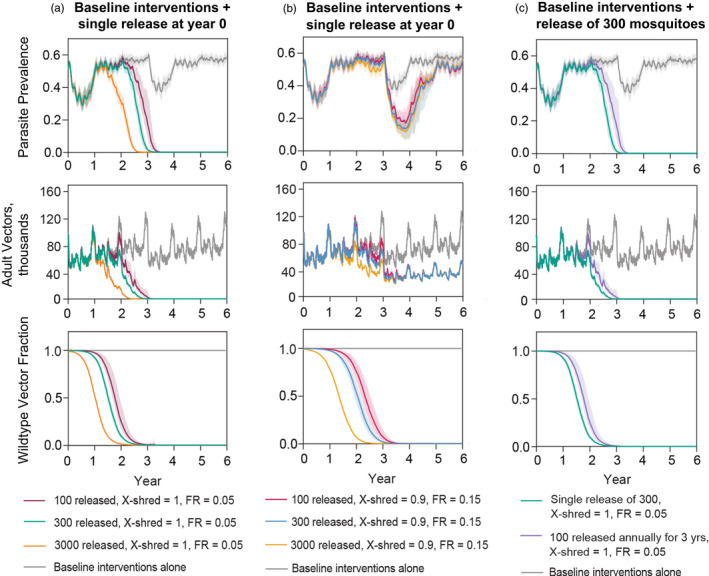
Selecting frequency and size of gene drive release in a nonspatial model in the presence of baseline ITN and ACT use. Single releases occurred at Year 0 and multiple releases were at year 0, 1, and 2. Shaded areas indicate 95% predicted intervals from 25 stochastic realizations. (a) Single release of a highly efficient drive. (b) Single release of a less efficient drive. (c) Single and multiple releases of a highly efficient drive. Results for the Equateur site are shown. Results for other sites are in Supporting information S2.3

We selected a gene drive release size and schedule by simulating highly efficient drives in the nonspatial framework (Figure 2, Supporting information 2) and identifying a schedule that could result in elimination. We simulated 25 stochastic realizations per candidate schedule. The range of 100–300 mosquitoes to release provided a sufficient number of gene drives to seed successfully in the majority of the simulations, avoiding stochastic die‐outs that occur at lower release numbers. The release size of driving‐Y mosquitoes in this study is smaller than those of previously developed genetically modified mosquitoes using other gene‐edited techniques (Alphey et al., 2010; Bouyer et al., 2020; Lees et al., 2015; Undurraga et al., 2016). Using a single release of 300 drives per node in the nonspatial framework, we varied the X‐shredder efficiency from 0.5 to 1.0 and fecundity reduction from 0 to 0.5, simulated 10 stochastic realizations per X‐shredder efficiency and fecundity reduction parameter combination, and evaluated whether malaria was eliminated. A simulation was defined as reaching malaria elimination when all‐age parasite prevalence in the model is not detectable, that is, dropped to zero and remained zero until the end of the 15‐year simulation timeframe. We selected parameter sets of X‐shredder efficiencies (0.9, 0.95, 1.0) and fecundity reductions (0.05, 0.1, 0.15) to generate 9 combinations of driving‐Y parameters that could eliminate malaria in the nonspatial framework (Supporting information 2).

The driving‐Y gene drive release strategy and parameters were then applied to the spatial simulation framework, where we identified which interventions or combinations of interventions could eliminate malaria in the selected locations. In all simulated scenarios, the interventions, including gene drive mosquitoes, were applied on the first day of year 0 unless indicated otherwise. All gene drive mosquitoes were released at the central node at the center of the selected 25x25km grid location on Day 1 of the beginning of the 15‐year simulation timeframe regardless of the seasonality of the site at the time of release. This timing corresponded to the tail end of the higher‐transmission season.

In the spatial simulation framework, vectors could move between adjacent nodes less than 10 km apart with a rate inversely proportional to their distance. Adjacent nodes included diagonally adjacent nodes. The vector migration rate ranged from 0.09 to 0.12 (9%–12% of female mosquitoes migrate out of a grid cell on any day). We did not include human migration in the simulations and found no difference in parasite reduction outcomes upon including human migration (Figure S6.2), likely because the simulated vector migration rates are high enough to stably suppress the vector population.

ITNs at 50% coverage and ACT at 19% coverage were applied uniformly across all nodes in baseline scenarios, which were used as the main comparator against other scenarios. Each of the individual ITNs and ACT (case management rate with ACT) and the combination of ITNs and ACT was analyzed at three levels of coverage: 50%, 80%, and 95%, following the selected coverage levels in Evans et al. (2005). If not specified, the coverage of ITN or ACT was 0%. Scenarios that failed to eliminate malaria were re‐simulated with the addition of a single release of 300 driving‐Y gene drive mosquitoes at the central node on the first day of Year 0. Nine combinations of X‐shredder efficiency and fecundity reduction, as described above, were tested. Each scenario (ITN/ACT/gene drive parameter combination) was simulated for 25 stochastic realizations.

We calculated Disability‐Adjusted Life Years (DALY) from model outputs—population by age group, uncomplicated clinical cases, severe cases, and deaths—at Year 5, Year 10, and Year 15 by giving equal weights to years of healthy life lost at young ages and older ages and with 0% discount rate for future lost years of a healthy life. The standard life expectancy at the age of death in years, and the DRC’s country lifetable (WHO, 2016b), and disability weights (GBD 2017 Causes of Death Collaborators, 2017) of moderate (0.051) and severe (0.133) were applied in DALYs calculation. DALYs averted (Salomon & Culyer, 2014; WHO, 2017) were then calculated by comparing outcomes to those of baseline scenarios.

Costs of all interventions, including gene drive mosquitoes, were calculated standardized to the year 2000 adjusted for inflation for the results to be comparable to those of previous WHO milestone studies (Evans et al., 2005; Morel et al., 2005). Estimated costs are expressed in international dollars ($int) (Tan‐Torres Edejer et al., 2003). Coverage‐dependent costs per person per year of applying ITNs, ACT, and the combination of ITNs and ACT were obtained from the WHO‐CHOICE database (WHO, 2020d) and a previous WHO study (Morel et al., 2005). For scenarios that included gene drive mosquito releases, we assumed the financial cost of gene drives as a single intervention ranged from 0.72 $int to 7.17 $int per person per year (Table 1) based on per‐person costs of vector control approaches that involve the release of mosquitoes to modify the vector population in previous studies (Meghani & Boëte, 2018; O’Neill et al., 2018; Undurraga et al., 2016) found in a systematic scoping review (Supporting information 8). We applied the US government consumer price index (CPI) (WHO, 2016b) to adjust for inflation and the cumulative inflation rates to the Year 2000 values. Costs of gene drive were added to the costs of any underlying intervention(s) also distributed (Table 1). Cost‐effectiveness was calculated for each 5‐year interval beginning in 2015 by dividing average yearly costs in $int by average yearly effectiveness in DALYs averted (Tan‐Torres Edejer et al., 2003). More cost‐effective interventions were identified by drawing a graph of an expansion path through the incremental cost‐effectiveness ratio (ICER), which uses the monetary value to compare the interventions (Tan‐Torres Edejer et al., 2003), and selecting interventions with more favorable cost‐effectiveness. The expansion path is drawn to connect the choices of interventions and present the order that the interventions would be chosen once more resources become available, considering only cost‐effectiveness. The additional cost required to avert each additional DALY, ICER, is the slope of each expansion path (Tan‐Torres Edejer et al., 2003).

**TABLE 1:** Estimated costs of interventions per year per one million population applied in the study

	Interventions	Coverage (%)	Cost per year ($int, millions) per one million population [i.e., cost per capita] using 2000 base year
Scenarios without gene drives	Insecticide‐treated bed nets (ITNs)	50	0.47
	Insecticide‐treated bed nets (ITNs)	80	0.63
	Insecticide‐treated bed nets (ITNs)	95	0.71
	Case management with artemisinin‐based combination therapy (ACT)	50	0.19
	Case management with artemisinin‐based combination therapy (ACT)	80	0.20
	Case management with artemisinin‐based combination therapy (ACT)	95	0.21
	Combination (ITNs and ACT)	50	0.68
	Combination (ITNs and ACT)	80	0.82
	Combination (ITNs and ACT)	95	0.74

	Interventions	Coverage (%)	Cost per year ($int, millions) per one million population [i.e., cost per capita] using 2000 base year	
			Lower bound	Upper bound
Scenarios with gene drives	300 gene drive mosquitoes with X‐shredder efficiencies = 1.0 alone	NA	0.72	7.17
	ITNs plus gene drives with X‐shredder efficiencies = 0.95 and 1.0	80	1.35	7.80
	ITNs plus gene drives with X‐shredder efficiencies = 0.95 and 1.0	95	1.43	7.88
	ACT plus gene drives with X‐shredder efficiencies = 0.95 and 1.0	95	0.93	7.38
	ITNs+ACT plus gene drives with X‐shredder efficiencies = 0.95 and 1.0	50	1.40	7.85
	ITNs+ACT plus gene drives with X‐shredder efficiencies = 0.95 and 1.0	80	1.54	7.99
	ITNs+ACT plus gene drives with X‐shredder efficiencies = 0.9, 0.95 and 1.0	95	1.46	7.91

## 3 RESULTS

This study uses mathematical modeling to explore the potential role of driving‐Y gene drives for malaria control and elimination in the DRC. Models were parameterized to capture malaria transmission in eight provinces that span the range of transmission seasonality and intensity across the DRC. Releasing gene drive mosquitoes lowers parasite prevalence in all modeled locations regardless of transmission intensity (Figure 2 and Supporting information 2). The elimination outcomes are similar in areas where transmission intensity is similar.

**FIGURE 3:**
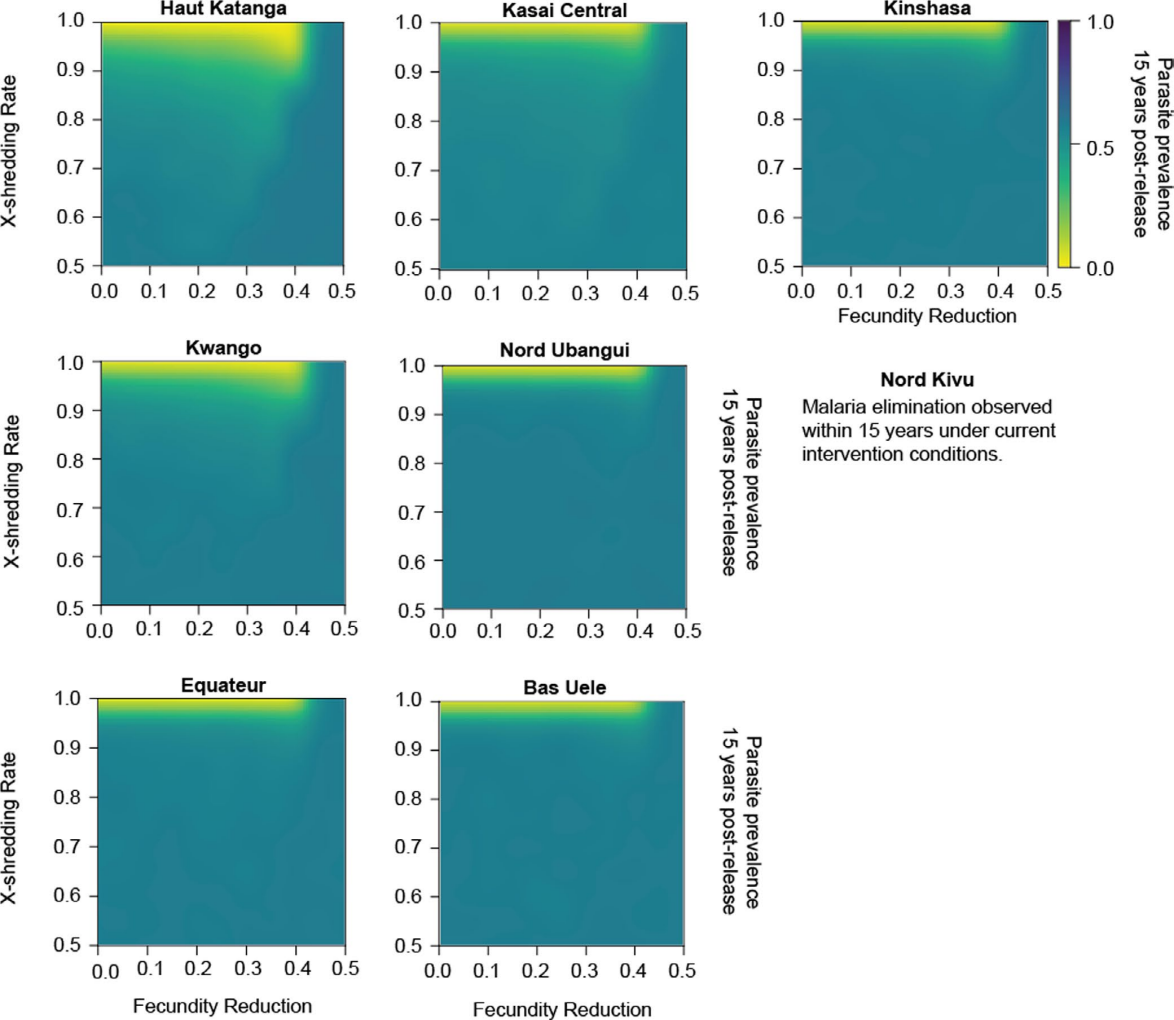
Dependence of final parasite prevalence on X‐shredder efficiency and fecundity reduction for the eight modeled sites in the nonspatial framework. Simulation outputs are means of 10 stochastic realizations per parameter combination and are observed 15 years after the release of 300 gene drive mosquitoes. See Supporting information 3 for upper and lower 95% predicted interval bounds and for outcomes with 100 or 200 mosquitoes released

Initial explorations were performed in a nonspatial simulation framework. Increasing the number of gene drive mosquitoes released from 100 to 300 and 3000 resulted in similar parasite prevalence reduction. In most locations, when compared to three releases of 100 gene drive mosquitoes with a one‐year interval between each release, a single release of 300 gene drive mosquitoes resulted in a similar reduction of parasite prevalence. Successful drives that could eliminate malaria within the 15‐year timeframe were those with very powerful X‐shredder efficiency at little to no cost of fertility (Galizi et al., 2014; Simoni et al., 2020). Across settings, gene drive was most successful at reducing malaria prevalence when the X‐shredder efficiency ranged from 0.95 to 1.0, and fecundity reduction ranged from 0 to 0.15. A similar sensitivity of mosquito population suppression to X‐shredder efficiency could be observed in previous studies using simpler models that studied mosquito population dynamics in a homogeneous and constant environment (Deredec et al., 2008, 2011) and an extended model that applied regional heterogeneity to model malaria mosquitoes at a national scale (North et al., 2019). In our study, the release of drives reduced parasite prevalence, adult vectors, and wild‐type vector fraction with a similar trend across all sites under a wide range of transmission intensity and seasonality (Figure 3, Supporting information 3). Our results also show that in areas where parasite prevalence is high, more efficient drives are required. This hindrance of suppression from possible higher productivity of the wild‐type population was also reported in previous modeling work (North et al., 2019). Furthermore, our study identified the range of driving‐Y parameter values (fecundity reduction and X‐shredder efficiency) that are likely to result in mosquito population suppression and malaria elimination in realistic settings.

The prevalence reduction aligns with the reduction in the number of adult vectors. In high transmission intensity sites, only drives with very high X‐shredder efficiencies (≥ 0.95) eliminated malaria. In lower‐transmission settings, drives with slightly lower X‐shredder efficiencies (0.9 to 0.95) could also eliminate malaria. These parameter ranges resulted in the elimination of vector populations. At X‐shredder efficiencies where malaria elimination was not observed, the impact on adult vector population size was larger than the impact on parasite prevalence (Supporting information 3). For spatial simulations, we selected a release strategy of a single release of 300 gene drive mosquitoes with fecundity reduction from 0.05 to 0.15 and X‐shredder efficiency from 0.9 to 1.0.

Interaction of gene drives with standard interventions, ITNs and ACTs, was simulated in a spatial model framework where gene drives, if used, were released at a single central location and allowed to diffuse outward. Under baseline conditions where interventions remain at constant levels, malaria elimination could not be achieved unless the area had low‐transmission intensity (Supporting information 2), which is the case for North Kivu. Regardless of transmission intensity, malaria elimination could be achieved by applying gene drives with an X‐shredder efficiency of 1.0 in all simulated settings (Table 2). Compared with nonspatial simulations under the same parameters, gene drives in spatial simulations took around 1 year longer to achieve elimination, and elimination was slightly less likely (Figure S5.8 in Supporting information 5). Rebounds of wild‐type vector fraction in spatial simulations were seen in a subset of realizations in all sites, although final suppression was usually observed by the end of Year 15. These rebounds could be driven by chasing dynamics where pockets of wild‐type mosquitoes avoid the gene drive, resulting in delays or failure of suppression (Bull et al., 2019; Champer et al., 2021; North et al., 2019).

**TABLE 2:** Minimum intervention or combination that can achieve malaria elimination in each target location within 15 years after adding driving‐Y mosquitoes into the simulated scenarios

Province	The minimal intervention(s) that could achieve malaria elimination								
Nord Kivu	Elimination is possible with interventions at pre‐existing levels.								
Intervention	ITNs			ACT			ITNs+ACT		
Coverage	50%	80%	95%	50%	80%	95%	50%	80%	95%
Haut Katanga	1.0	0.95	0.95	1.0	1.0	0.95	0.95	NA	NA
Kwango	1.0	1.0	0.95	1.0	1.0	1.0	1.0	0.95	NA
Kasai Central	1.0	1.0	0.95	1.0	1.0	1.0	1.0	0.95	NA
Nord Ubangui	1.0	1.0	0.95	1.0	1.0	1.0	1.0	0.95	NA
Bas Uele	1.0	1.0	1.0	1.0	1.0	1.0	1.0	0.95	0.9
Kinshasa	1.0	1.0	1.0	1.0	1.0	1.0	1.0	0.95	0.9
Equateur	1.0	1.0	1.0	1.0	1.0	1.0	1.0	0.95	0.9

Note

When gene drives were applied, multiple X‐shredder efficiencies were simulated. The X‐shredder efficiency in the table is the lowest X‐shredder efficiency that could result in malaria elimination. Orange color: malaria elimination without gene drives. Blue color: malaria elimination with gene drives. 1.0: gene drives with X‐shredder efficiency = 1.0. 0.95: gene drives with X‐shredder efficiencies = 0.95 and 1.0. 0.9: gene drives with X‐shredder efficiencies = 0.9, 0.95 and 1.0.

Abbreviations: ACT, case management rate with artemisinin‐based combination treatment (Artemether + Lumefantrine); ITNs, insecticide‐treated nets; NA, not applicable, gene drives were not applied in the scenarios because malaria elimination was achieved with the indicated intervention combination without gene drives.

Malaria elimination was achievable without gene drive mosquitoes by combining high coverage of both ITNs and ACT in Haut Katanga (80% coverage of both). In contrast, elimination was not achievable in Kwango, Nord Ubangui, and Kasai Central at these coverage levels, showing the need for new tools and echoing conclusions of recent eradication evaluation commissions (Feachem et al., 2019; WHO, 2020b). For all remaining modeled sites with moderate to high parasite prevalence (18.6%, 32.6%, and 60.7% in Bas Uele, Equateur, and Kinshasa provinces, respectively), a single release of single species 300 driving‐Y mosquitoes with an X‐shredder efficiency of 1.0 and fecundity reduction between 0.05 and 0.15 eliminated malaria within 15 years (Table 2). In the simulations, we assumed a single vector species of *An*. *gambiae* as this species dominates transmission in the DRC. However, results are generalizable to other species or multi‐species systems if multiple species‐specific drives are released. Reduction in transmission intensity relative to baseline was more sensitive to changes in the X‐shredder efficiency than to fecundity reduction (Supporting information 4). The models predicted that malaria elimination could be achieved with gene drive mosquitoes within 7 years, and in many of these scenarios, it was achieved 4 years postrelease (Supporting information 5), as long as X‐shredder efficiency was very high.

DALYs averted estimated from the model's outputs show similar trends as those of WHO (Table S7.1 in Supporting information 7). Population‐level cost‐effectiveness estimates for individual and combined interventions as costs per DALY averted in comparison with the baseline scenario indicate that DALYs averted, rather than cost, is the main factor determining cost‐effectiveness across interventions (Table S7.2 in Supporting information 7). In scenarios with gene drives that resulted in malaria elimination, the costs per DALY averted are lower in areas where the transmission intensity is initially higher as there were more DALYs to avert in the high transmission area with comparable costs between scenarios and the costs decrease over time as DALYs continue to be averted after elimination is achieved (Table 3).

**TABLE 3:** Average yearly cost per one million population and mean parasite prevalence reduction for interventions and combinations of interventions applied in the study for scenarios that could result in malaria elimination. 95% confidence intervals are presented in (lower, upper). (See Table S7 and Supporting information 7 for complete estimates of all scenarios.)

	Intervention	Coverage	WHO’s	Estimates from model's outputs			Transmission intensity
			Average yearly costs per one million population ($int, million)			Mean parasite prevalence reduction from baseline over 15 years (%)	
Scenarios without gene drives	ITNs and ACT	80%	0.82	0.82 (0.82, 0.82)		0.36	Low
	ITNs and ACT	95%	0.74	0.74 (0.74, 0.74)		0.36	Low
				0.74 (0.74, 0.74)		0.48	Medium
**Cost calculation range**				**Lower bound**	**Upper bound**		
Scenarios with gene drives	300 gene drive mosquitoes with X‐shredder efficiencies = 1.0 alone	NA	Not covered in WHO’s study	0.72 (0.72, 0.72)	7.17 (7.17, 7.17)	0.23	Low
				0.80 (0.62, 0.97)	7.94 (6.20, 9.68)	0.36	Medium
				0.72 (0.72, 0.72)	7.17 (7.14, 7.19)	0.36	High
	ITNs plus gene drives with X‐shredder efficiencies = 0.95 and 1.0	80	Not covered in WHO’s study	1.35 (1.35, 1.35)	7.81 (1.35, 1.35)	0.28	Low
				Elimination could not be achieved			Medium
				Elimination could not be achieved			High
	ITNs plus gene drives with X‐shredder efficiencies = 0.95 and 1.0	95	Not covered in WHO’s study	1.43 (1.43, 1.43)	7.89 (7.88, 7.88)	0.33	Low
				1.43 (1.43, 1.43)	7.88 (7.87, 7.89)	0.42	Medium
				Elimination could not be achieved			High
	ACT plus gene drives with X‐shredder efficiencies = 0.95 and 1.0	95	Not covered in WHO’s study	0.93 (0.93, 0.93)	7.39 (7.38, 7.38)	0.32	Low
				Elimination could not be achieved			Medium
				Elimination could not be achieved			High
	ITNs and ACT plus gene drives with X‐shredder efficiencies = 0.95 and 1.0	50	Not covered in WHO’s study	1.40	7.86	0.32	Low
				Elimination could not be achieved			Medium
				Elimination could not be achieved			High
	ITNs and ACT plus gene drives with X‐shredder efficiencies = 0.95 and 1.0	80	Not covered in WHO’s study	Achieved elimination without gene drives			Low
				1.54 (1.40, 1.40)	7.99 (7.85, 7.86)	0.46	Medium
				1.54 (1.54, 1.54)	7.99 (7.98, 8.00)	0.47	High
	ITNs and ACT plus gene drives with X‐shredder efficiencies = 0.9, 0.95 and 1.0	95	Not covered in WHO’s study	Achieved elimination without gene drives			Low
				Achieved elimination without gene drives			Medium
				1.46 (1.46, 1.46)	7.91 (7.89, 7.93)	0.49	High

Note

Transmission intensity in study areas: Low: Haut Katanga; Medium: Kwango, Kasai Central, Nord Ubangui; High: Bas Uele, Kinshasa, Equateur.

**FIGURE 4:**
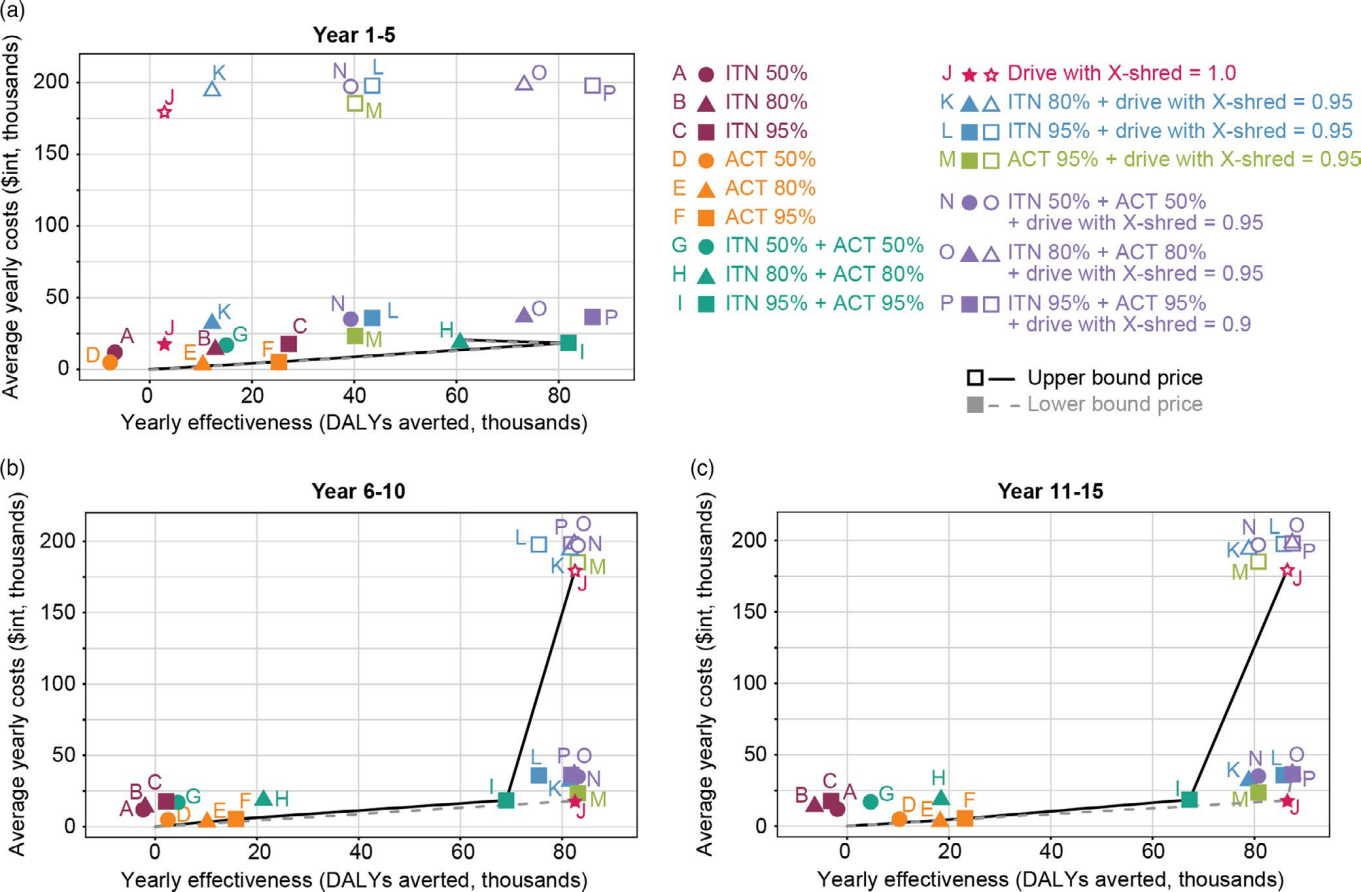
Cost‐effectiveness plane showing 16 intervention packages (10 individual and combination interventions at three assumed coverage levels) and expansion paths for Year 1–5, Year 6–10, and Year 11–15. The dashed lines (filled symbols) are expansion paths based on the lower bound, and the solid lines (empty symbols) are expansion paths based on the upper bound costs of gene drives

The expansion paths of all sites show the order in which interventions would be selected at different levels of resources available based on the ICER (Figure 4, Table 4). The ICER indicates additional costs required to avert each additional DALY by moving from the lower‐cost to the higher‐cost intervention (Tan‐Torres Edejer et al., 2003). It is calculated using average yearly costs and yearly effectiveness (Table 3). Notable differences exist between the first and the following two 5‐year intervals. The combination of ITNs and ACT at 95% coverage is the most cost‐effective intervention plan. However, it is not clear that 95% coverage of either ACT or ITNs as single interventions or in combination would be achievable under the estimated costs, as even with high expenditures, such levels of coverage have not yet been achieved (Feachem et al., 2019; Haakenstad et al., 2019). For the first interval, the high‐coverage combinations of ITNs and ACT are more cost‐effective (Figure 4, Table 4, and Tables S7.3 and S7.4 in Supporting information 7). In the following years (second and third intervals), the unit cost of gene drive mosquitoes affects the priority of the strategies on the expansion path as gene drives become more cost‐effective compared with other interventions (Figure 4).

**TABLE 4:** Intervention packages on expansion path points in Figure 4

	Time interval from mosquito release			
	Year 1–5	Year 6–10	Year 11–15	Point on expansion path
Lower bound price	95% ACT	300 gene drive mosquitoes with X‐shredder efficiencies = 1.0 alone		1^st^
	95% ITN and ACT	95% ACT plus gene drives with X‐shredder efficiencies = 0.95 and 1.0	95% ITNs and ACT plus gene drives with X‐shredder efficiencies = 0.9, 0.95 and 1.0	2^nd^
	80% ITN and ACT	50% ITNs and ACT plus gene drives with X‐shredder efficiencies = 0.95 and 1.0	80% ITNs and ACT plus gene drives with X‐shredder efficiencies = 0.95 and 1.0	3^rd^
Upper bound price	95% ACT			1^st^
	95% ITN and ACT			2^nd^
	80% ITN and ACT	300 gene drive mosquitoes with X‐shredder efficiencies = 1.0 alone		3^rd^

The cost of gene drives as a single intervention is calculated in International Dollars ($int), a hypothetical unit of currency that has the same purchasing power that the U.S. Dollar has in the United States at a given point in time (Tan‐Torres Edejer et al., 2003). Costs of gene drives ranged from 0.72 $int to 7.17 $int per person per year (Table 1). The calculation is based on the costs of vector control approaches that involve the release of mosquitoes to modify the vector population, per person, in previous studies (GBD 2017 Causes of Death Collaborators, 2017; WHO, 2015a). In case of combinations, the cost of gene drives was added to the cost of other interventions. Using the lower bound price for the cost of gene‐edited mosquitoes, gene drive as a single intervention is the most cost‐effective intervention overall as gene drive mosquitoes with an X‐shredder efficiency of 1.0 could eliminate malaria in all contexts and would be the first choice where resources are limited. The effect, however, could be seen after elimination was achieved from the second 5‐year interval onward. If malaria elimination cannot be achieved, 95% ACT coverage is the most cost‐effective intervention. The result suggests that, if gene drives with a 100% effective construct, an X‐shredder efficiency of 1.0 and fecundity reduction of 0.05–0.15, are available, and their cost is comparable to other existing interventions, gene drives would be the most cost‐effective single intervention for malaria elimination. It is advisable to maintain existing interventions, especially ACT and ITNs, early on, while gene drives are propagating. Once the mosquito population collapses, gene drives become more cost‐effective over a medium timeframe.

## 4 DISCUSSION

This study uses mathematical modeling to describe the potential role of sex‐ratio‐distorting gene drive mosquitoes in malaria control across the transmission spectrum in the DRC, an area where achieving effective control has historically been challenging. Our results suggest that population suppression through gene drives could be an effective strategy for malaria elimination in the DRC, either as a single intervention or in combination with other interventions. To the best of our knowledge, this is the first study that models the epidemiological impact and cost‐effectiveness of gene drive mosquitoes for malaria elimination. Previous studies involving gene drives for malaria control are limited in scope to laboratory experiments (Akbari et al., 2014; Curtis, 1968; Galizi et al., 2016; Pike et al., 2017), and the development and parameterization of mathematical models (Godfray et al., 2017; Heffel & Finnigan, 2019; Noble et al., 2019; North et al., 2019). By extending previous modeling work (Eckhoff et al., 2017) to approximately estimate the cost‐effectiveness of gene drive in SSA settings, our work helps fill a gap in evidence about the programmatic implementation of gene drives in the context of limited resources. The study not only estimates the feasibility of gene drives in realistic malaria elimination scenarios but also evaluates the cost of gene drives in comparison to other currently available interventions. This work helps gauge the probabilities of success and possible outcomes of gene drives that are strictly laboratory‐contained or in the transition from the laboratory‐based research to future field‐based research. Introducing modified organisms into the environment can be invasive, and preventive measures should be in place to provide timely mitigation in case of spillovers and countermeasures to halt an ongoing gene drive when necessary. Multiple safeguards will be needed in parallel (WHO, 2020a). In addition to the technical perspective provided in this study, further work is necessary, including on the ethical perspective, that is, standard research ethics, procedural ethics, and participatory management of the technology (Thompson, 2018), as a key component to implement this technology in wild mosquito populations (Wedell et al., 2019).

We found that the success of driving‐Y gene drives in all areas regardless of vector density highly depends on the ability of gene drives to shred the X chromosome. Though a naturally occurring driving‐Y chromosome that transmits >90% of male progeny can be found in *Aedes* and *Culex* mosquitoes (Burt & Crisanti, 2018) and a CRISPR‐based X‐shredder can generate up to 100% male bias in the laboratory (Galizi et al., 2014; Simoni et al., 2020), driving‐Y gene drives have yet to be developed in the laboratory. Fecundity does not appear to be a major detriment to gene drives that do not directly target female fecundity (Kyrou et al., 2018). The adoption of a driving‐Y strategy could be very challenging because it may be difficult to achieve a perfect X‐shredder efficiency at every development stage and during implementation while overcoming the challenge of meiotic sex chromosome inactivation (Thompson, 2018). Moreover, possible resistant mutants could convert wild‐type genes and spread resistance (Beaghton et al., 2017; Bull et al., 2019; Champer et al., 2021), and cleavage resistant alleles have already been observed in *An*. *gambiae* (Galizi et al., 2014).

Some sex‐ratio‐distorting drives may not be comparable to Y drive or X shredders, for example, if the female carries a drive that inactivates the reproduction of her male progeny. However, the difference in mechanisms by which sex ratio distortion is achieved may only lead to differences in how quickly a drive establishes itself rather than downstream outcomes regarding elimination, which is the focus of this study. The models predict that reaching malaria elimination does not always require bringing the number of mosquitoes down to zero and that there is continued mosquito biting after releasing gene drive mosquitoes. Our finding that the drive must be highly effective while mild fecundity costs are well‐tolerated is likely generalizable to other suppression drives as a whole, although further explorations in settings of stronger seasonality are needed. Our economic findings on the cost‐effectiveness of drives are likely to be order‐of‐magnitude similar for any highly effective suppression drive.

The success of suppressive gene drives such as driving‐Y depends on mosquito population size and allowing enough time for the drives to propagate in the mosquito population. Understanding interactions between existing vector control methods such as ITNs and IRS that temporarily reduce the mosquito population (Alphey et al., 2010; WHO, 2015a) and gene drives will be necessary given that vector control typically reduces mosquito populations. While this work focuses on the impact of vector abundance, seasonality, and conventional vector control on gene drive outcomes, spatial connectivity of mosquito populations and terrain heterogeneity will also have important implications for gene drives success. Accurate capture of local variation in mosquito population connectivity requires data on mosquito swarms and habitats at high resolution, mosquito movement patterns, and mosquito species introgression. Some of these quantities are measurable and known but most are unavailable for the DRC. A sensitivity analysis with vector migration rate reduced down to 3 orders of magnitude did not observe substantial change in elimination outcomes (Supporting information 6, Figure S6.1), although reduced migration led to later elimination.

Our models predicted that high coverage with ITNs and high access to treatment with ACTs could eliminate malaria in lower‐transmission settings, but achieving such high coverages of existing measures is not only extremely difficult but also comes with high implementation and logistical costs (Shretta et al., 2017; Zelman et al., 2014). It may take much more investment in logistics and systems to achieve 95% coverage of both ITNs and ACT than WHO’s estimates applied in the study (Table 1) (Haakenstad et al., 2019). Even if theoretically achievable, it is highly improbable to sustain necessary coverage levels in the complex operational environment of high disease burden countries like the DRC (Carrel et al., 2015; WHO, 2005). The costs estimated in the previous WHO study and applied in this work considered program level, patient level, and opportunity costs of currently available interventions in the respective WHO subregion. These estimated costs might still underestimate the costs of existing interventions given the difficulty for widespread distribution of interventions and reliability of program monitoring in the DRC (Haakenstad et al., 2019; PNLP, SwissTPH, KSPH, NRB, & INFORM, 2014). Given the challenges of continued conflict and political unrest in the DRC, gene drives could offer reliable malaria control in areas where it is difficult to deploy and monitor currently available interventions. Considering the potential percolation properties of gene drives, especially in areas where mosquito populations are more connected, successful gene drives could plausibly be more cost‐effective if the cost is comparable to currently available interventions. However, this may not be the case for areas with more sparsely connected mosquito populations and higher rates of gene pool variability (Dhole et al., 2020). Other cost components, for example, surveillance, will also contribute to the cost of gene drive once implemented. Depending on the nature of surveillance and whether a response is also needed, these maintenance costs could accumulate to substantial amounts.

Our model results show that tailoring the frequency of releases and the number of gene drive mosquitoes to be released can make malaria elimination achievable within 5 years (Supporting information 5) after a single release of gene drive mosquitoes under certain conditions, including but not limited to no importation of vectors or infections. Importation can trigger local transmission and cause resurgences (Sturrock et al., 2015). Future work that includes importation of vectors and infections is necessary to address the feasibility of sustained elimination, and to specify release schedules that are operationally practical, technically necessary for intended deployment areas, and appropriate for the local seasonality. Because of their self‐propagating and self‐sustaining properties (Hammond & Galizi, 2017), gene drives would likely result in better cost‐effectiveness once implemented compared to other genetically engineered mosquitoes previously developed (e.g., sterile insect techniques). Nonetheless, the payoffs are only observed once malaria elimination is reached—in most cases, after 5 years postrelease in the settings considered in this study. This waiting period can be critical, given many life losses in the interim in the DRC’s context. Our results highlight the importance of efficient gene drives over simply increasing the number of gene drive mosquitoes released. This aligns with the result of a recent study using the simplest model of a population with one life stage and density‐dependent mortality that the diffusion rate of Y drive males depends on the strength of drive (Beaghton et al., 2016).

We based our cost‐effectiveness analysis on the unit costs of OX513A (Alphey et al., 2018) and *Wolbachia*‐infected mosquitoes per person (Meghani & Boëte, 2018), since cost data of genetic control methods are limited. We performed a systematic scoping review (Supporting information 8) to search for evidence in costs of vector control approaches that involve the release of mosquitoes to modify vector population and selected moderate lower and upper bound costs to give approximate values. The rationale to apply the cost of gene drives per person protected instead of using the cost per gene drive mosquito is to be conservative in approaching the cost estimation given the low number of gene drive mosquitoes released in the models in this study.

The lower bound cost applied in this study is from a study on *Wolbachia*‐infected mosquitoes, where costs came from expenses for the entire deployment (O’Neill et al., 2018). These estimates are somewhat uncertain because future deployments would likely utilize less monitoring in an operational public health program than in research and occur in settings of higher population density. The self‐maintaining of *Wolbachia* postdeployment would also reduce ongoing costs. The upper bound cost is from studies on the Oxitec mosquito (Meghani & Boëte, 2018; Undurraga et al., 2016). One study source did not explicitly state the cost components (Meghani & Boëte, 2018) and the other modeled estimated costs based on preliminary estimates by Oxitec (Undurraga et al., 2016). Thus, different situations and contexts may naturally lead to deviation in costs from these estimates. The use of Oxitec mosquitoes requires surveillance efforts and recurrent re‐licensing from the patent‐owning company, which could further increase costs. The lower and upper bound range in costs applied in the study reflects the reality in the field, as the genetic control methods vary in cost components even though the methods were developed to tackle the same disease (WHO, 2005). Future research should explore the cost components of gene drives, especially development, operational, and environmental costs, that may contribute to changes in the overall cost of this type of intervention. The changes in unit cost could affect the cost‐effectiveness of the method if the cost is too high. As we demonstrated in the ICER analysis, the cost‐effectiveness is cost‐sensitive. The gene drive approach in malaria elimination is also effectiveness‐sensitive and becomes less cost‐effective compared to other strategies once its cost increases or effectiveness decreases or both.

Gene drive technology is at an early stage of development and concerns over ethics, safety, and governance, as well as questions on affordability and cost‐effectiveness, must be addressed before implementation (WHO, 2020a). For high malaria burden countries such as the DRC, collaborating on testing, implementing, and regulating new technologies like gene drives poses challenges not only from within the country but also with other countries where different systems of governance can further complicate the collaborations (Dambach et al., 2014). Presently, many countries including the DRC have insufficient resources to individually follow recommendations such as extensive risk assessment and safety testing, and close monitoring after mosquito releases (WHO, 2020a), making it a challenge to enforce legislation required under the Cartagena Protocol (Kingiri & Hall, 2012). Since organisms know no political boundaries, this challenge presents an opportunity for international collaboration to focus on effective strategies rather than political benefits and agendas. Further research on risk assessments and step‐wise implementations may help reduce uncertainties and characterize potential risks and benefits that involve crucial ethical and social challenges (Committee on Gene Drive Research in Non‐Human Organisms, 2016; The Royal Society, 2018).

This study demonstrated a modeling approach applied to *An*. *gambiae*, the predominant malaria vector in Africa (The *Anopheles gambiae* 1000 Genomes Consortium, 2017). The methodology developed here can be applied to other malaria‐transmitting mosquito species. In settings with multiple major malaria vectors, releasing an equally effective gene drive for each major vector would approximate the impact of targeting *An*. *gambiae* in this study. The study identified key aspects of both gene drive technology and its implementation that are fundamental for the technology to be a cost‐effective component of a malaria control program. Amid uncertainty about vector abundance and its behavior (Guerra et al., 2014) and no importation of infections and wild‐type mosquitoes in the models, the study offers an evaluation framework. The framework can be generalized to look at other gene drive approaches to effectively plan gene drive strategies in malaria control, especially in other high burden countries where parasite transmission intensity varies.

## CONFLICT OF INTEREST

The authors state that there are no competing interests.

## Supporting information

Supplementary Material

Table S7

## Data Availability

The data that support the findings of this study are available in GitHub repositories as follows.
gene drive: https://github.com/NaniMet/gene‐drive
dtk‐tools: https://github.com/InstituteforDiseaseModeling/dtk‐tools
EMOD: https://github.com/InstituteforDiseaseModeling/EMOD
