# Supplementary material for: Modeling impact and cost‐effectiveness of driving‐Y gene drives for malaria elimination in the Democratic Republic of the Congo
Source: Evol Appl. 2022 Jan 7;15(1):132–48. doi: 10.1111/eva.13331 (PMC8792473; doi:10.1111/eva.13331)
Supplement: Supplementary file 1 — Supplementary Material [file EVA-15-132-s001.docx]

**Supplementary: Modeling impact and cost-effectiveness of driving-Y gene drives for malaria elimination in the Democratic Republic of the Congo**

**Contents**

[Supplementary 1: Site selection process 2](#_Toc78533717)

[Supplementary 2: Non-spatial simulation framework: baseline outputs and release schedule scenarios for all study sites 3](#_Toc78533718)

[Supplementary 3: Non-spatial simulation framework: driving-Y parameters of gene-drive mosquitoes single release at year 0 13](#_Toc78533719)

[Supplementary 4: Non-spatial simulation framework: ratio between current and initial numbers of adult vectors, 15-year post- single release of 300 drive mosquitoes at year 0 17](#_Toc78533720)

[Supplementary 5: Spatial simulation framework: simulation outputs 18](#_Toc78533721)

[Supplementary 6: Sensitivity of results to vector and human migration 33](#_Toc78533722)

[Supplementary 7: Cost-effectiveness analysis 39](#_Toc78533723)

[Supplementary 8: Costs of vector control approaches that involve the release of mosquitoes to modify vector population – A systematic scoping review 47](#_Toc78533724)

# Supplementary 1: Site selection process

We selected provinces based on DRC-DHS 2013-14’s stratification (1) (please refer to Figure 1 in main texts): 1 site for the first stratum that contains only one province, Nord Kivu, 3 testing sites from the second stratum consisting of 15 provinces, another 3 testing sites from the third stratum consisting of 9 provinces, and 1 site for the last stratum that has only one province.

The provinces were randomly selected within each stratum but ensured that the selected sites covered the MAP PfPR range of low, medium, and high within the same stratum. Within each selected province, we randomly selected the central node coordinates from the reported DRC-DHS 2013-2014’s survey points and checked against WorldPop that all points within the grid chosen were populated, although we did not use WorldPop population estimates to set node populations since we are exploring a generalized setting for the province. The DRC has a low population density of 35.9 people per square kilometer and 40% of the population live in the urban area (2) such that we found many parts of the country had no reported population data. Climate and seasonality of the individual node coordinates were used to simulate seasonality of vector habitat availability and hence malaria transmission intensity.

1. President’s Malaria Initiative, FY 2019 Democratic Republic of Congo Abbreviated Malaria Operational Plan (2019) (available at https://www.pmi.gov/docs/default-source/default-document-library/malaria-operational-plans/fy19/fy-2019-democratic-republic-of-the-congo-abbreviated-malaria-operational-plan.pdf?sfvrsn=5).

2. esri, esri-DRC (2021), (available at https://www.arcgis.com/apps/Cascade/index.html?appid=7905c9073ee64033b3f168d2adf13617).

# Supplementary 2: Non-spatial simulation framework: baseline outputs and release schedule scenarios for all study sites

***Figure S2.1 Adult vectors outputs of baseline scenarios observed throughout a year of each study location***


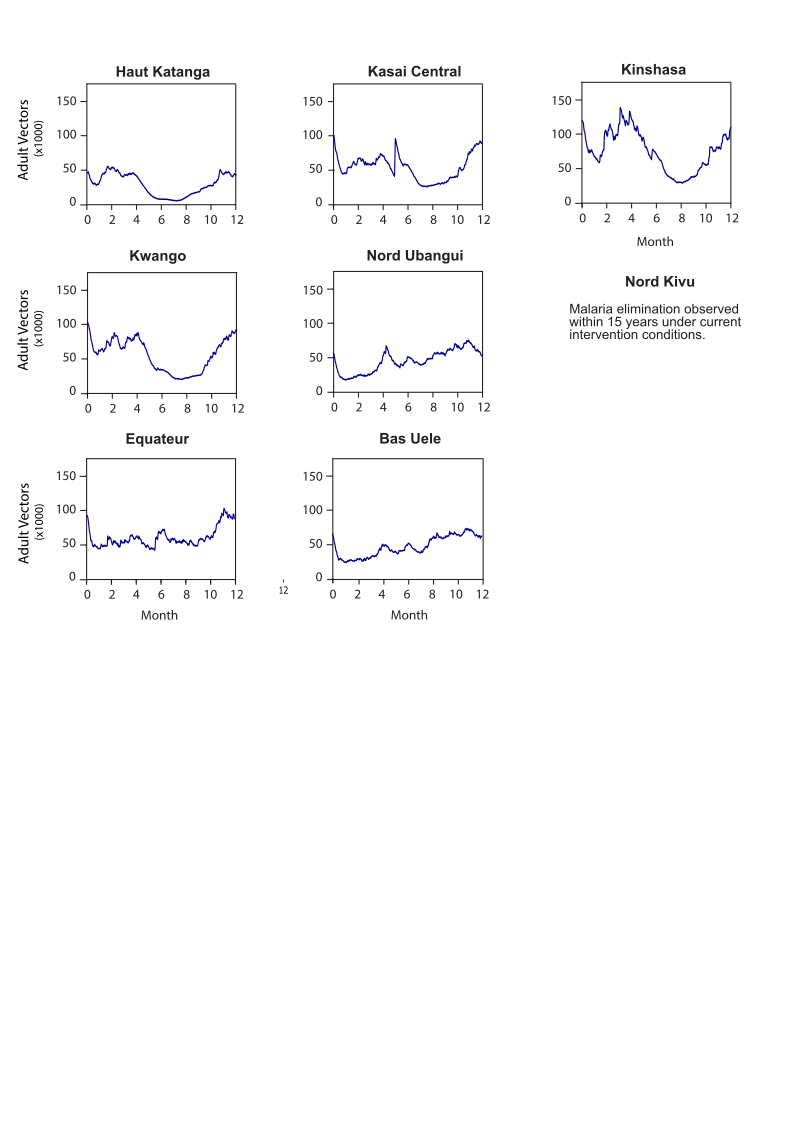


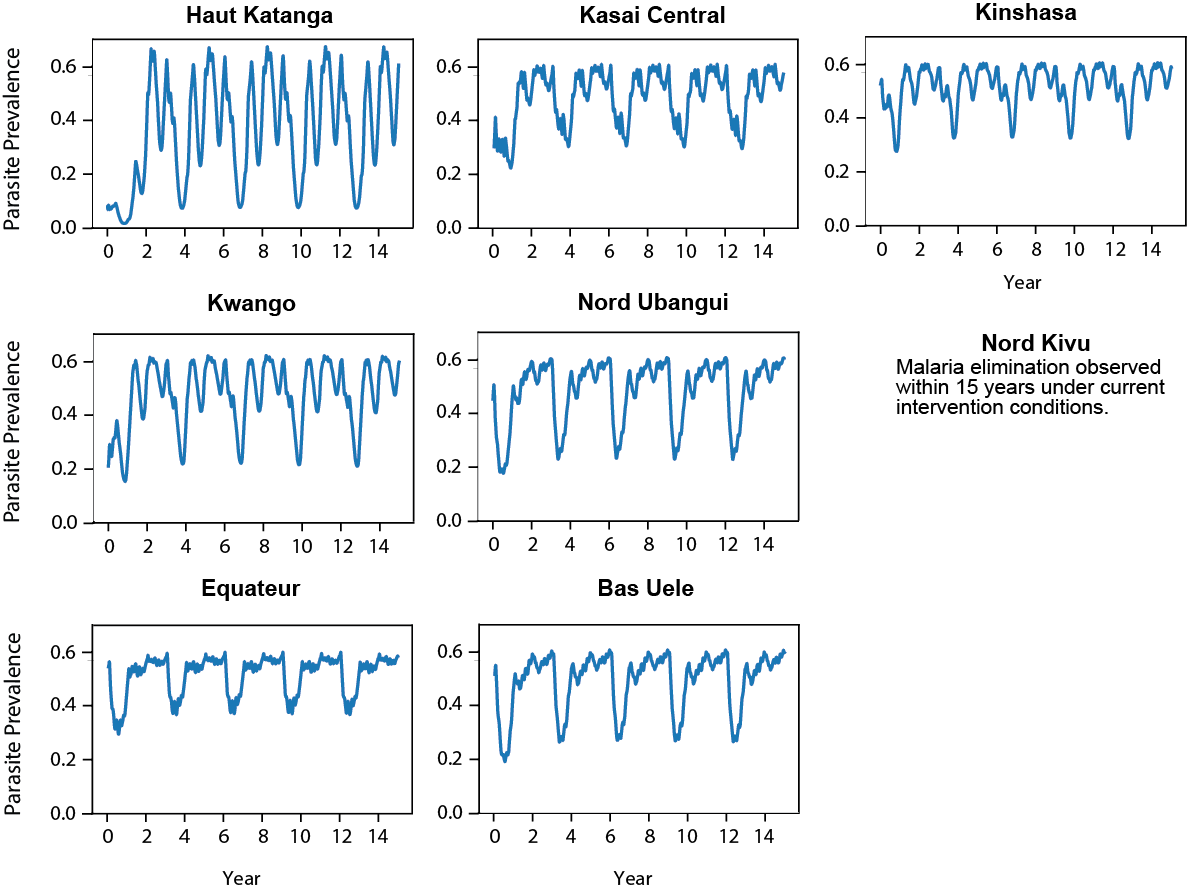
***Figure S2.2*** ***Parasite prevalence and adult vectors outputs of baseline scenarios observed throughout 15-year simulation timeframe of eight study locations:***


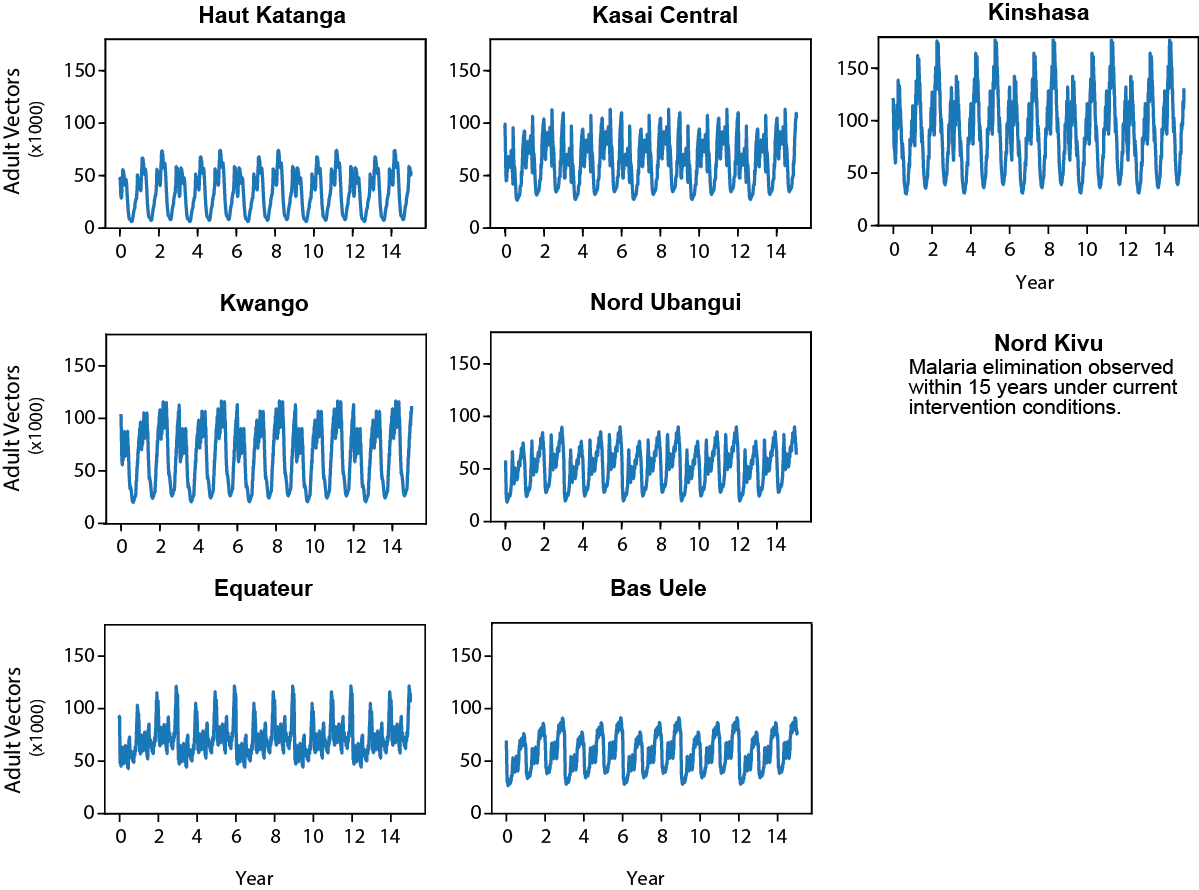


***Figure S2.3 Non-spatial simulation framework: number and frequency of driving-Y gene-drive mosquitoes released.* *Simulation outputs by site****:*

*In column A, the single release of a sufficient number of efficient gene drives, which is 300 gene drives in this study context, could eliminate malaria within 3.5 years. Increasing the number of gene drives released to 3,000 gene drives (10 times) did little in accelerating the malaria elimination (less than 6 months of the period to achieve elimination) compared to a single release of 300 gene drives. Highly efficient gene drives (column A) could eliminate malaria and less efficient gene drives (column B) could almost eliminate malaria. The single and multiple releases of the same total number of gene drives (column C) yielded similar malaria reduction.*

**Bas Uele**

**
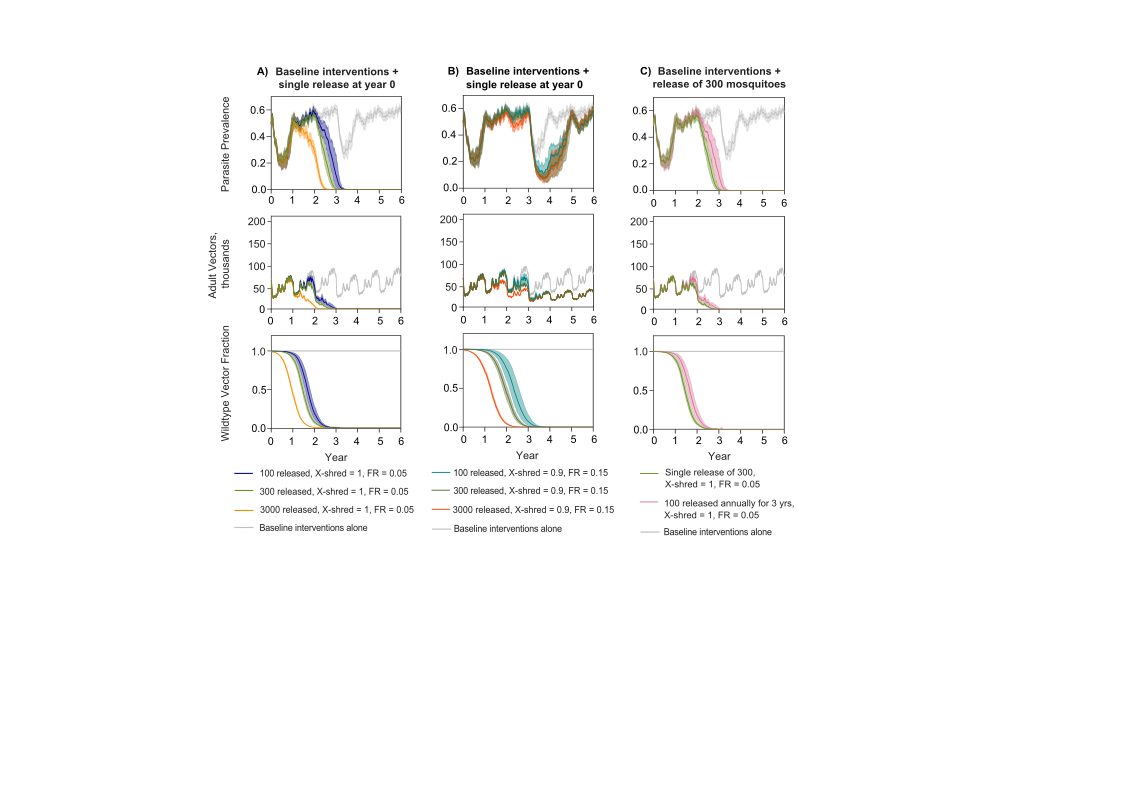
**

**Equateur**

**
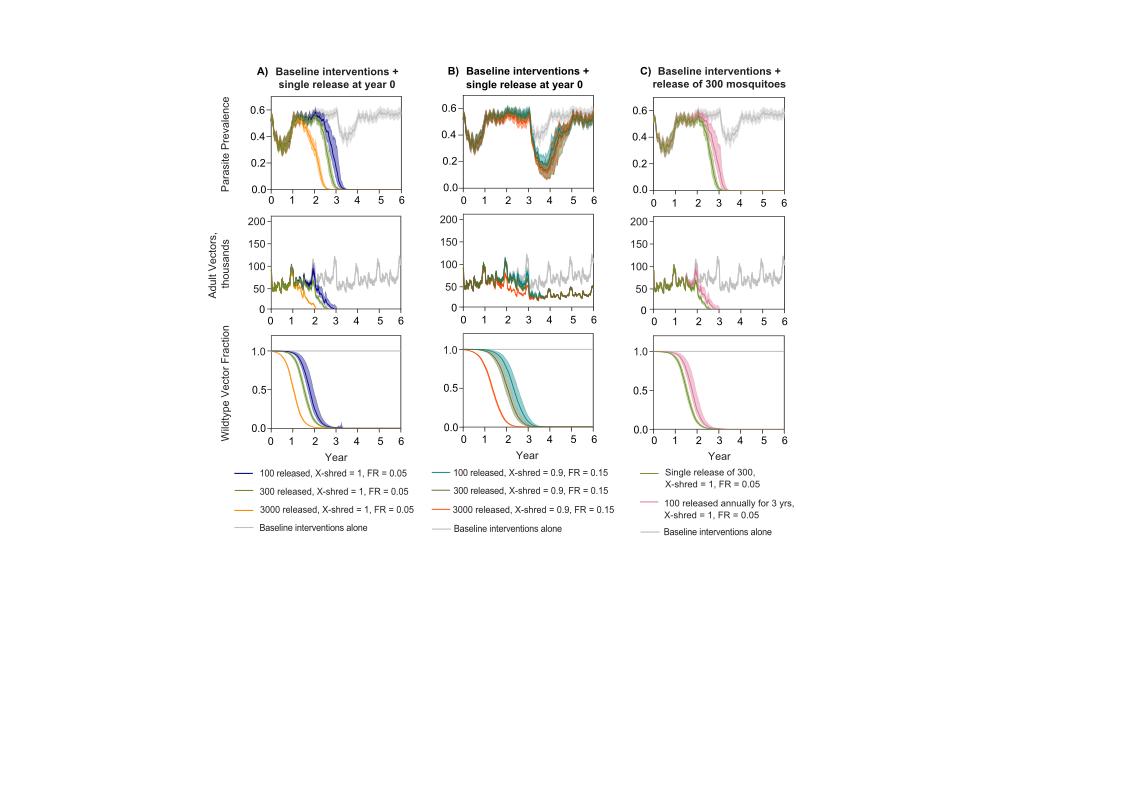
**

**Haut Katanga**

**
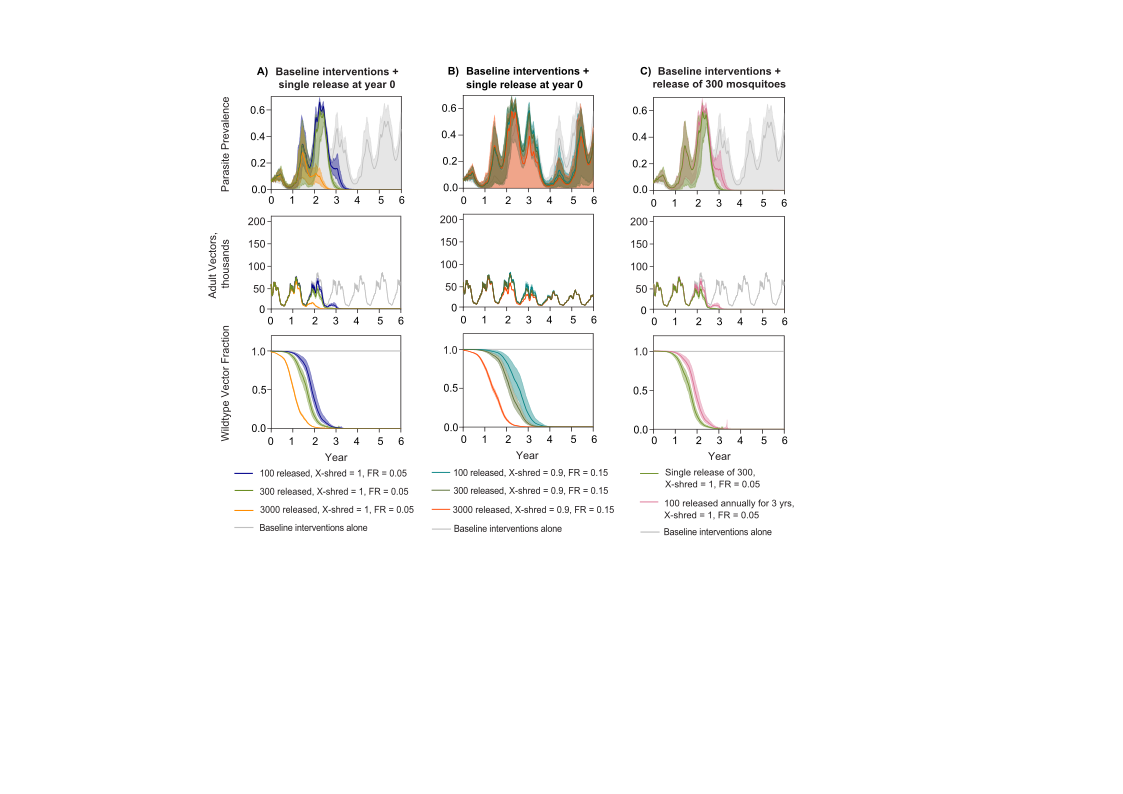
**

**Kasai Central**

**
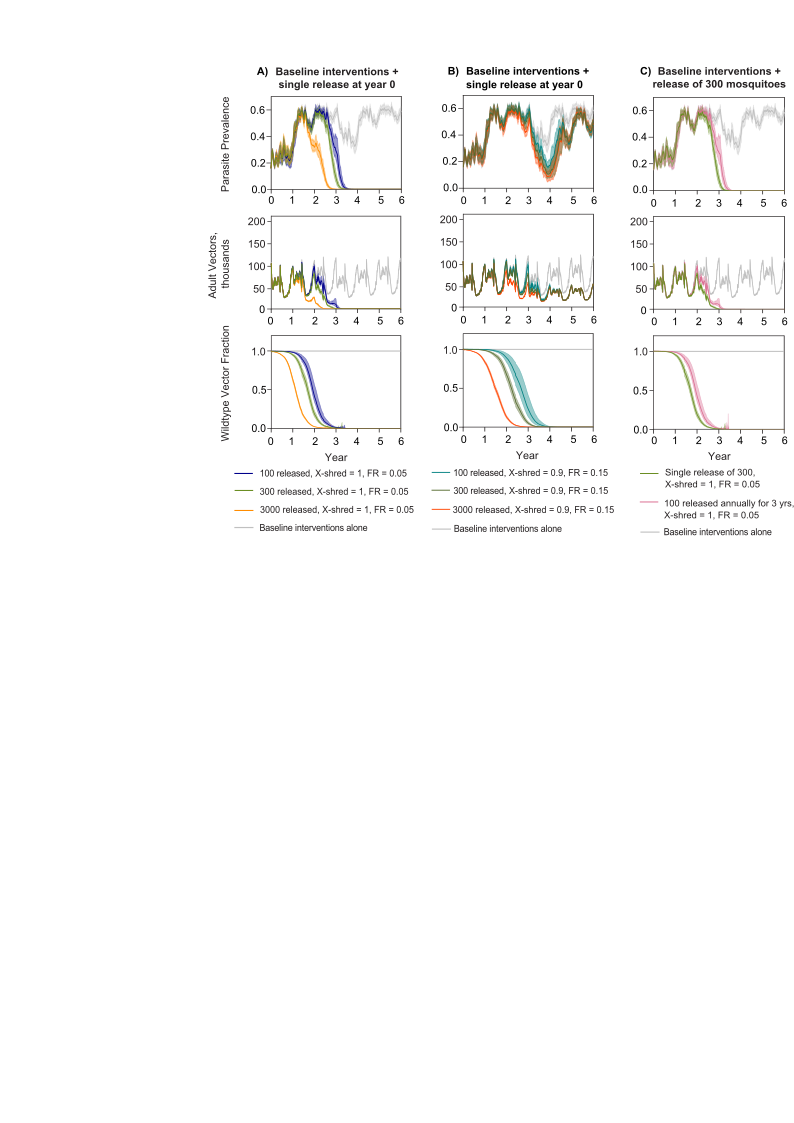
**

**Kinshasa**

**
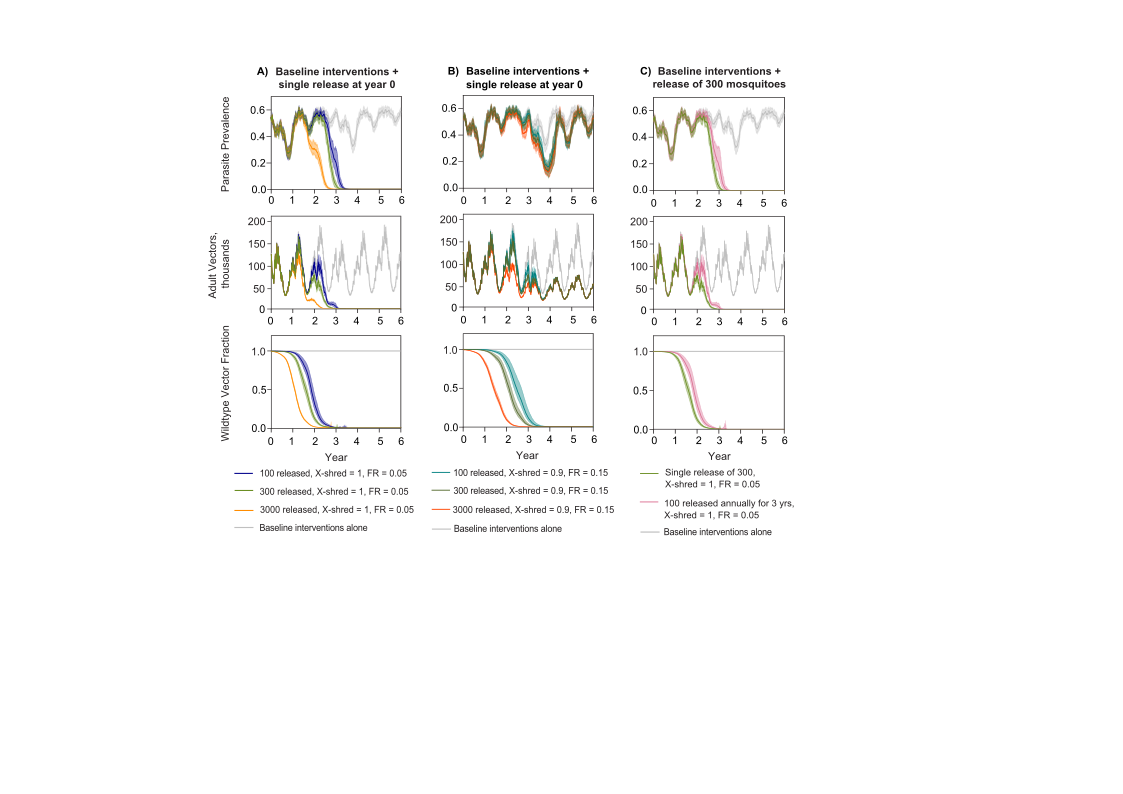
**

**Kwango**

**
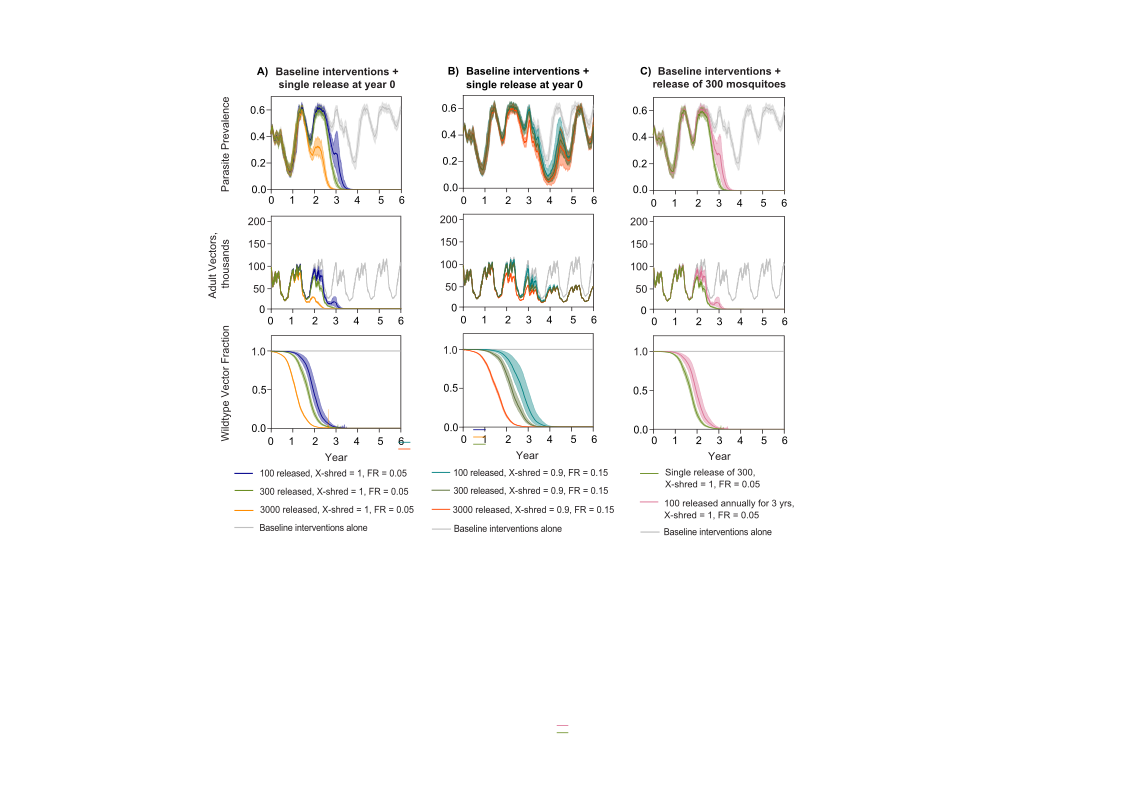
**

**Nord Ubangui**

**
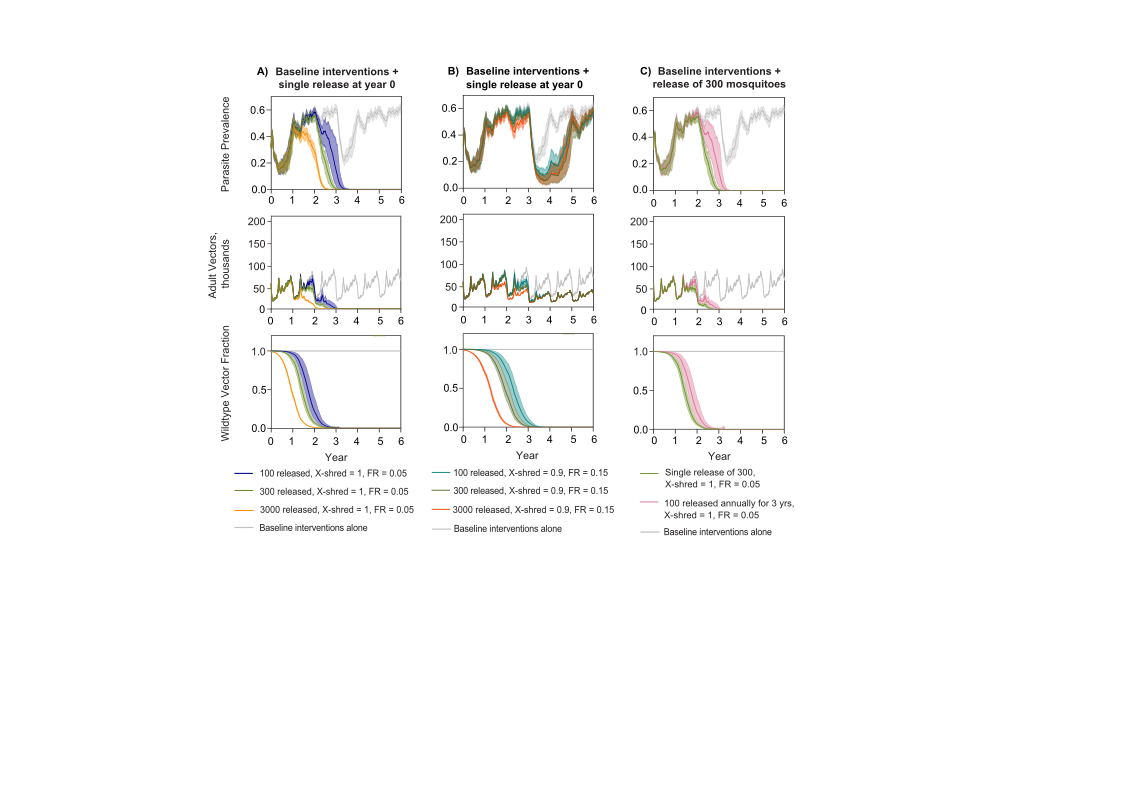
**

# Supplementary 3: Non-spatial simulation framework: driving-Y parameters of gene-drive mosquitoes single release at year 0

***Figure S3.1 Simulation outputs, observed at the end of 15-year simulation timeframe, post-release of 300 gene drive mosquitoes at year 0 in the non-spatial framework of eight study locations by driving-Y parameter values:***

***with 2.5% quantiles***


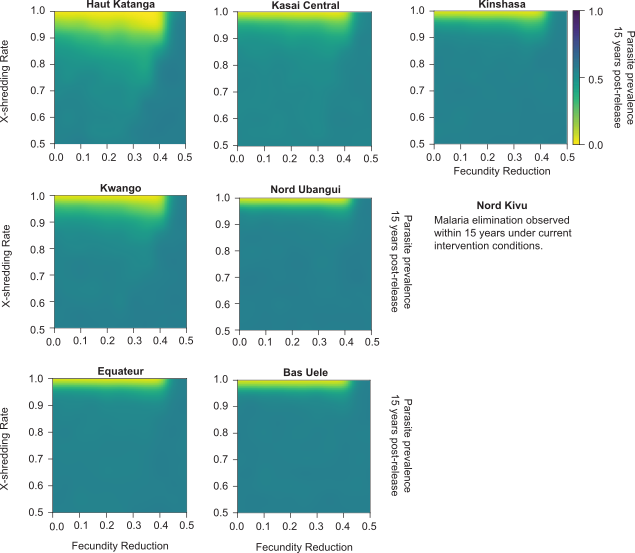


***with 97.5% quantiles***


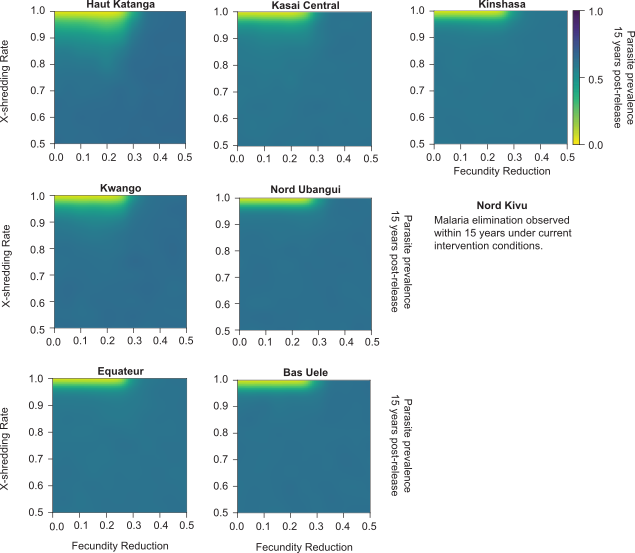


***Figure S3.2*** Non-spatial simulation framework: For each site, rows: releases of 100, 200, or 300 gene drive mosquitoes; columns: years post-release. Each box shows parasite prevalence under various driving-Y parameters.


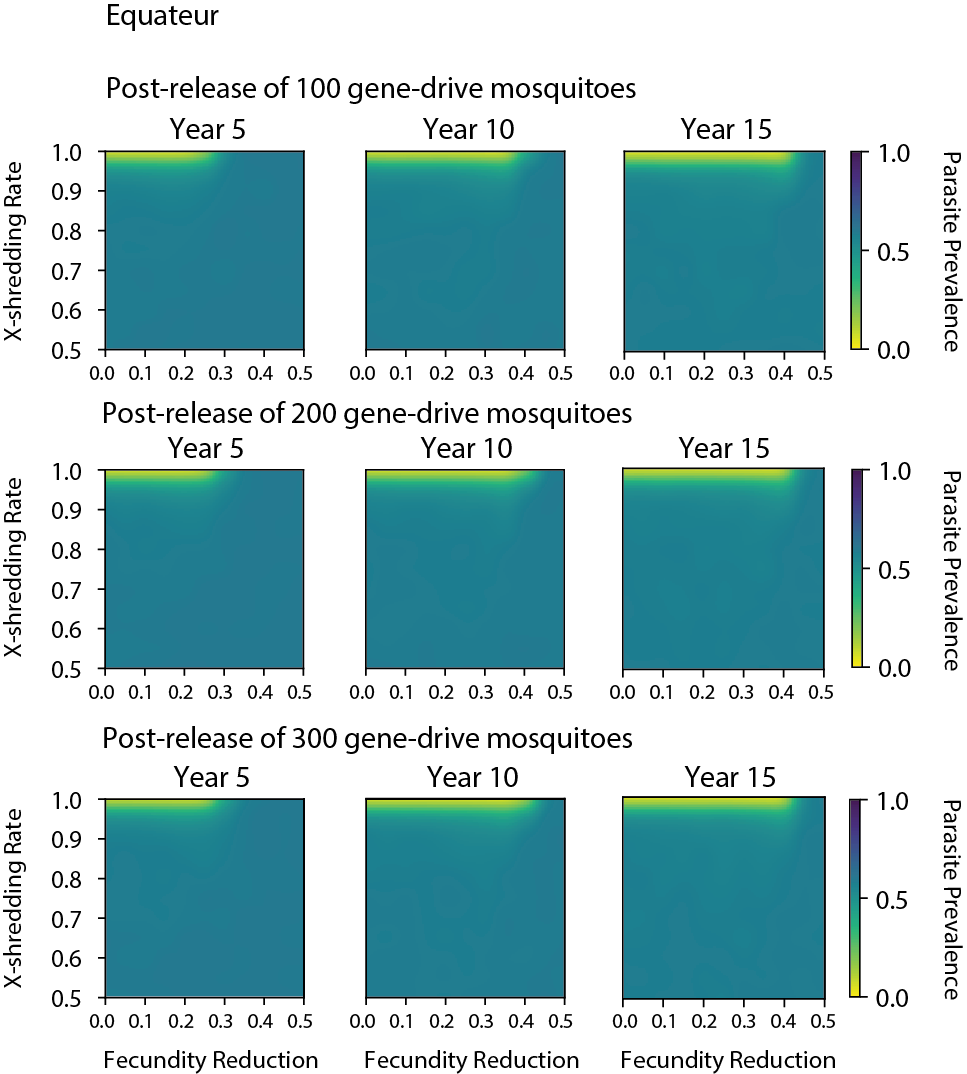

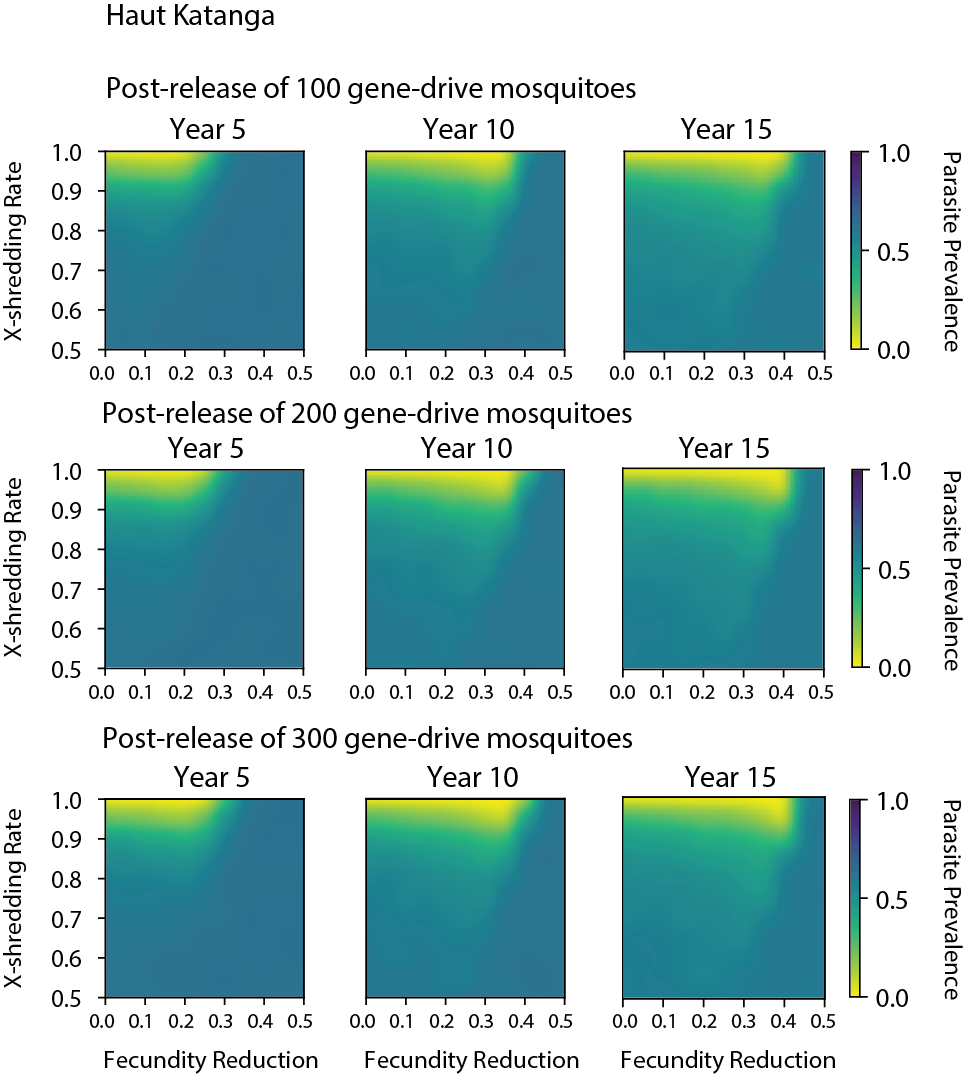


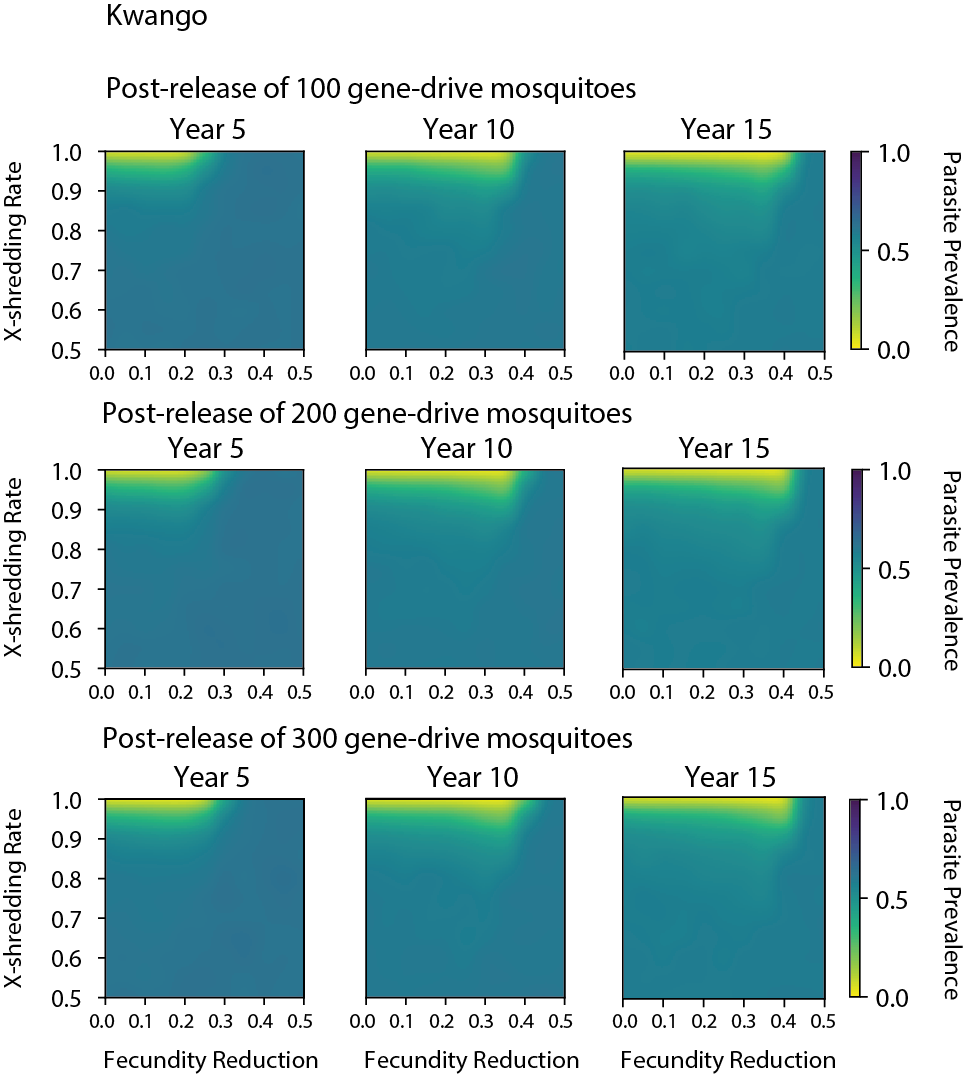

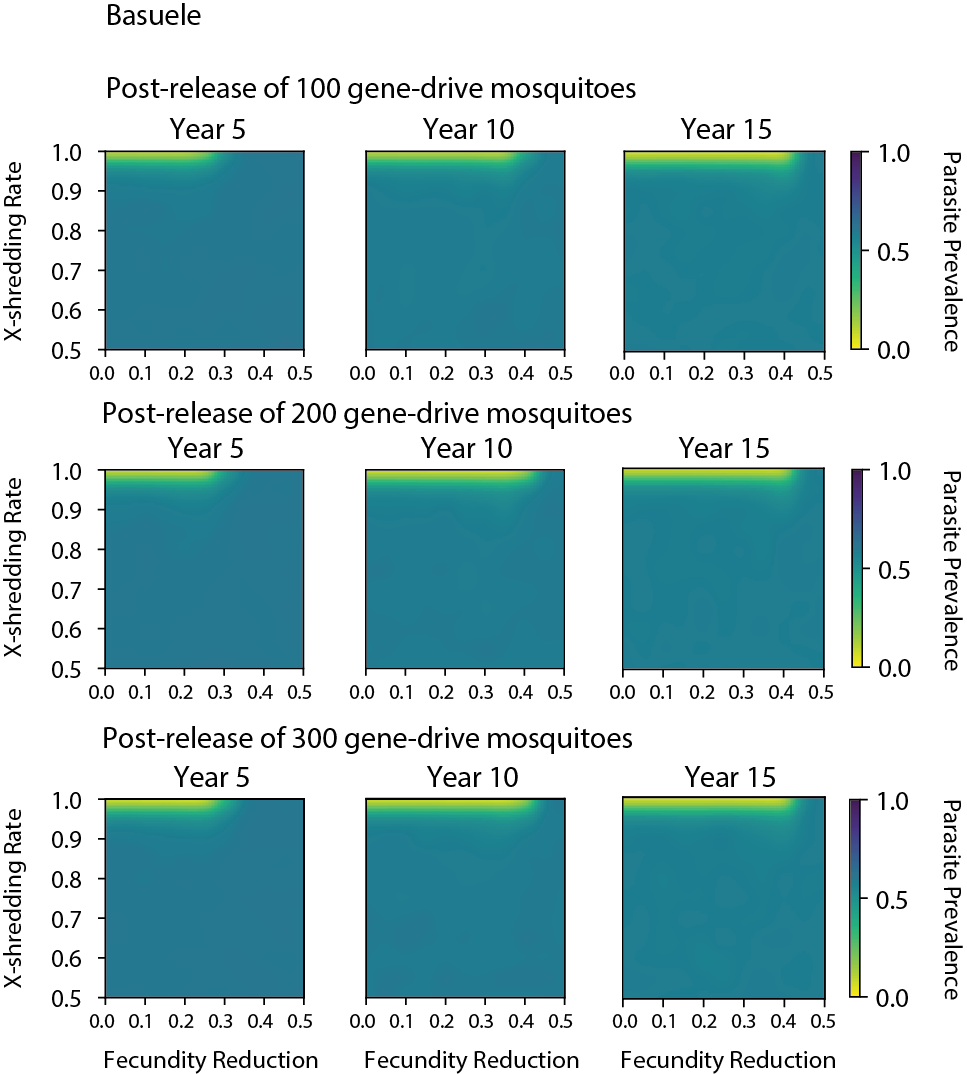


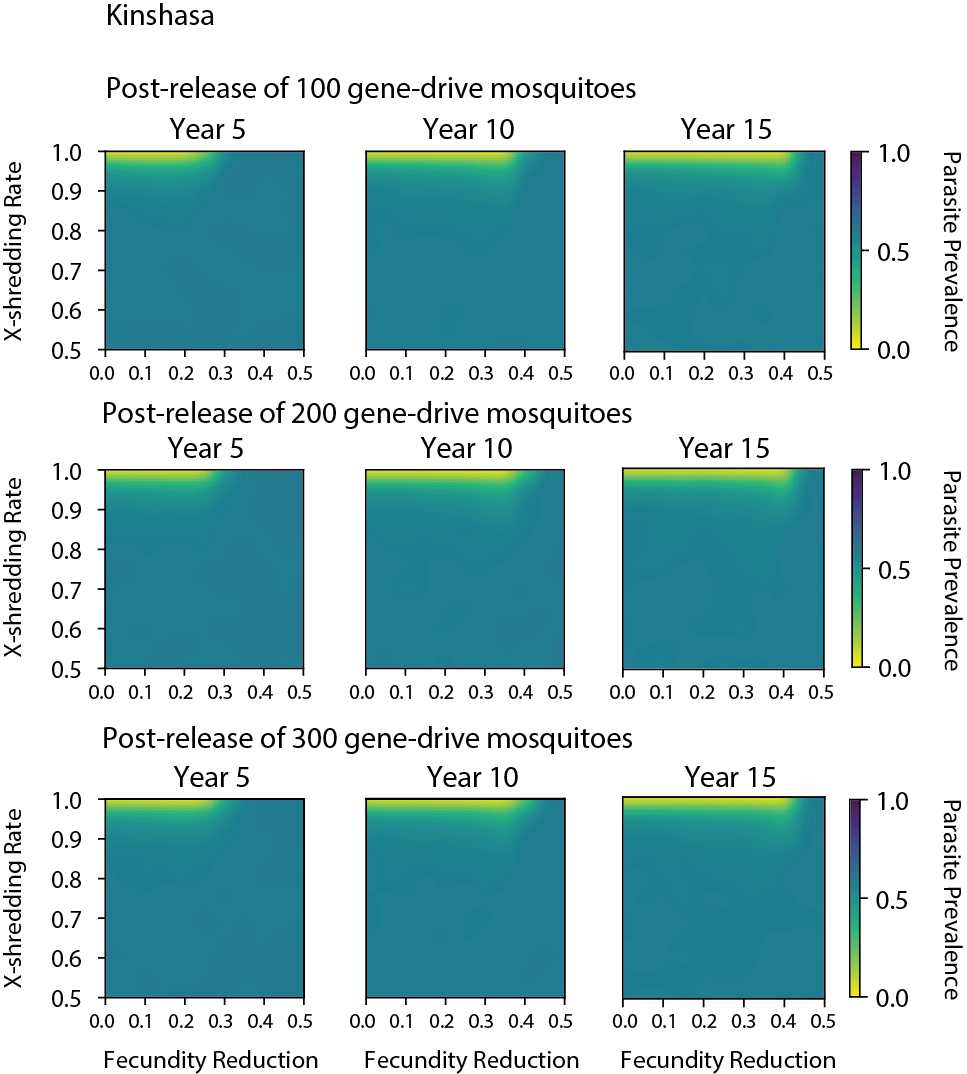

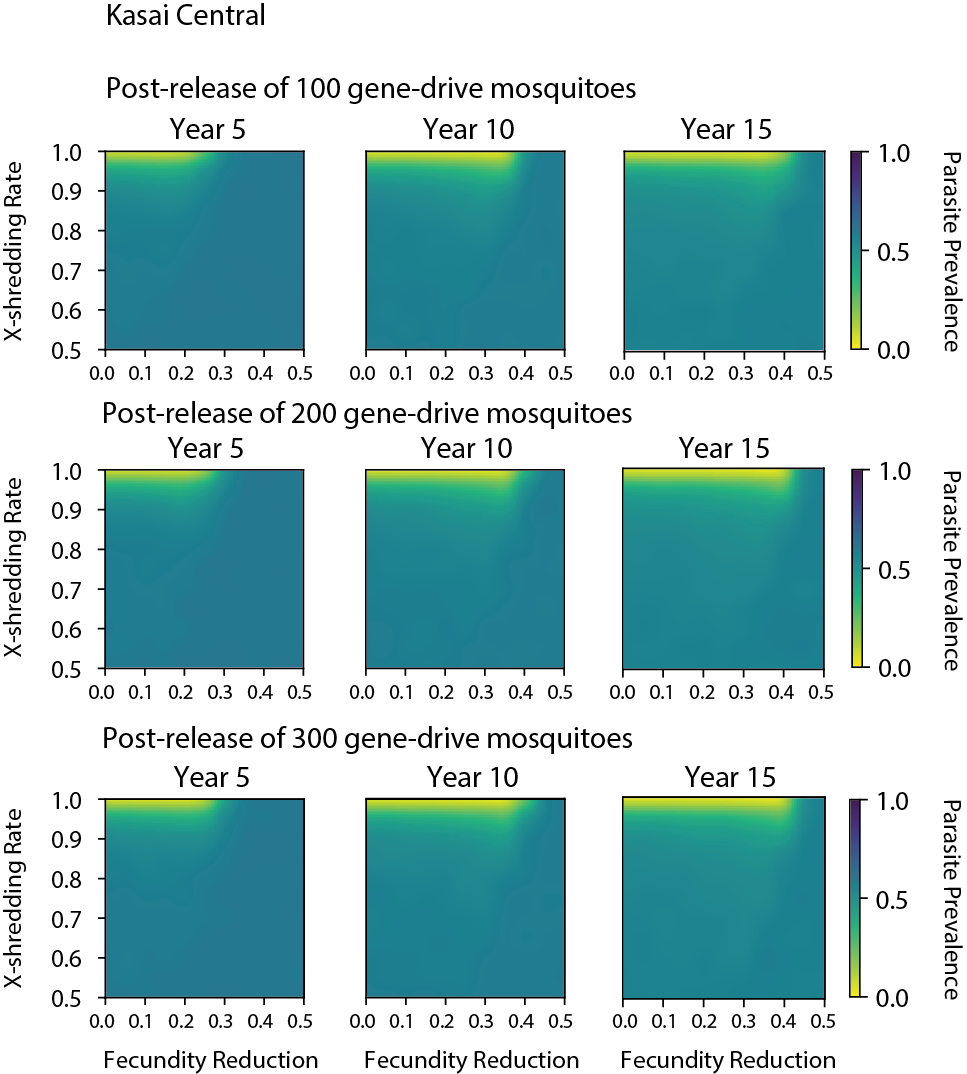

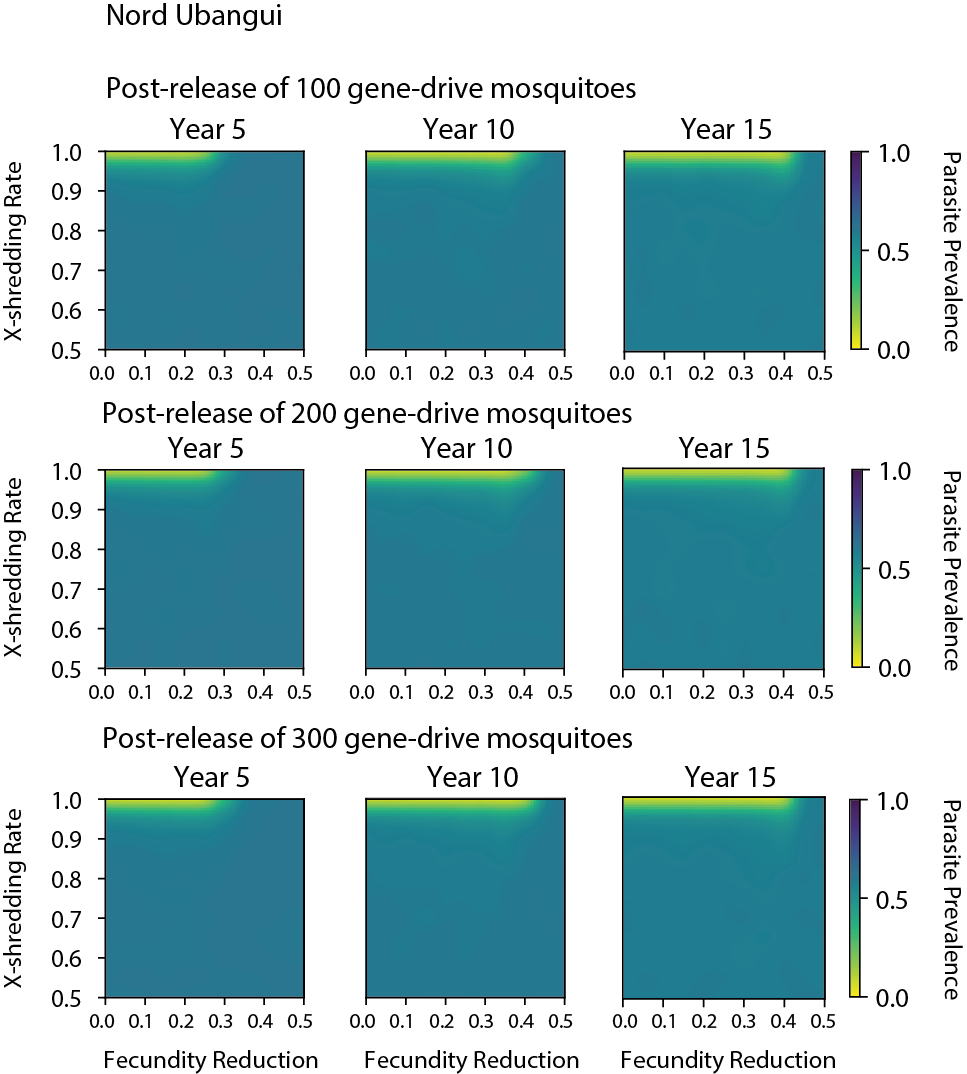


# Supplementary 4: Non-spatial simulation framework: ratio between current and initial numbers of adult vectors, 15-year post- single release of 300 drive mosquitoes at year 0


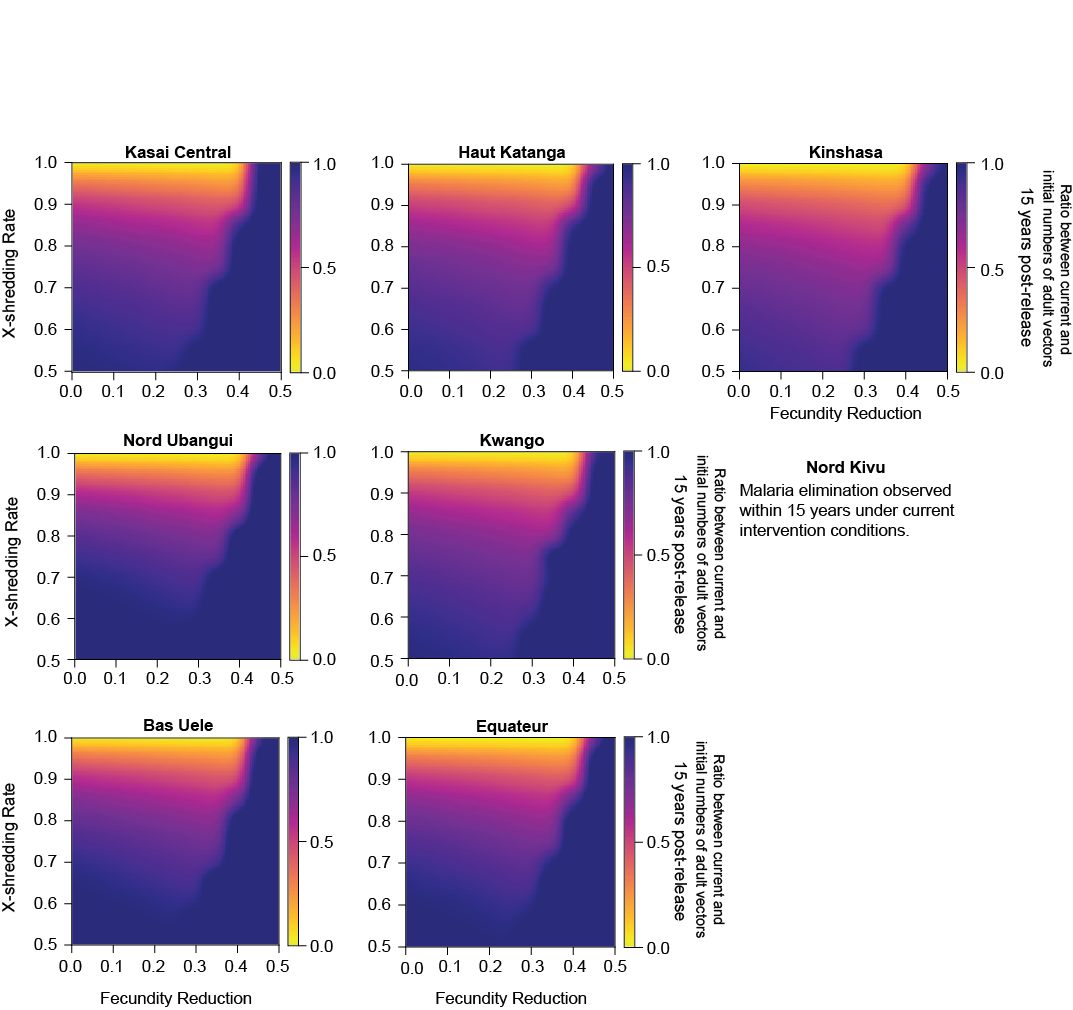


# **Supplementary 5: Spatial simulation framework: simulation outputs.**

Figure S5.1-5.7: Shaded areas indicate 95% predicted intervals from 25 stochastic realizations.

***Figure S5.1 Bas Uele***

A) ACT vs Baseline B) ITN vs Baseline C) ITN+ACT vs Baseline


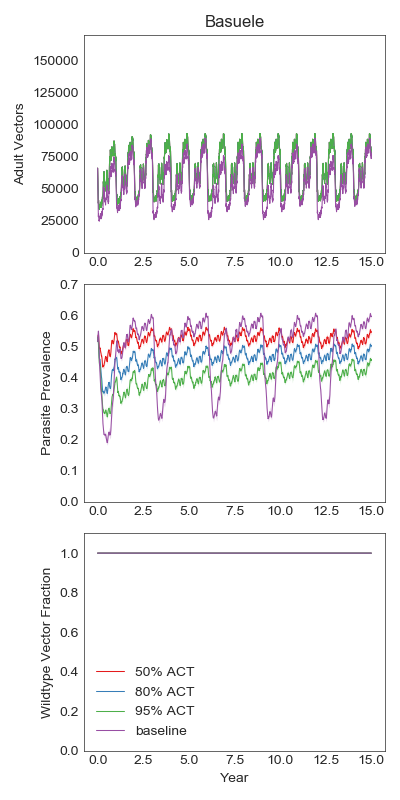

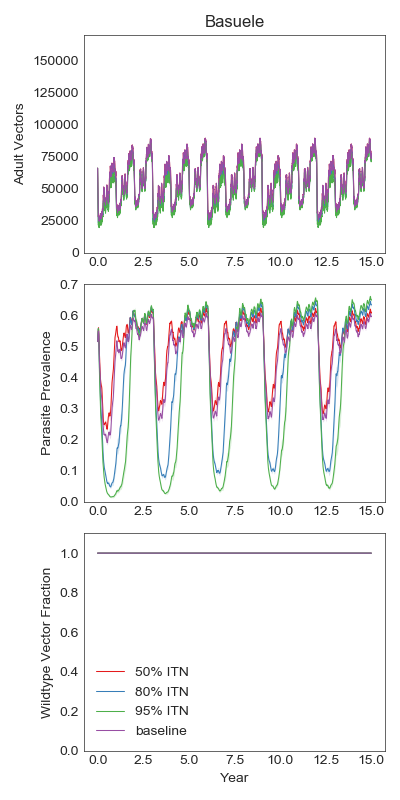
**
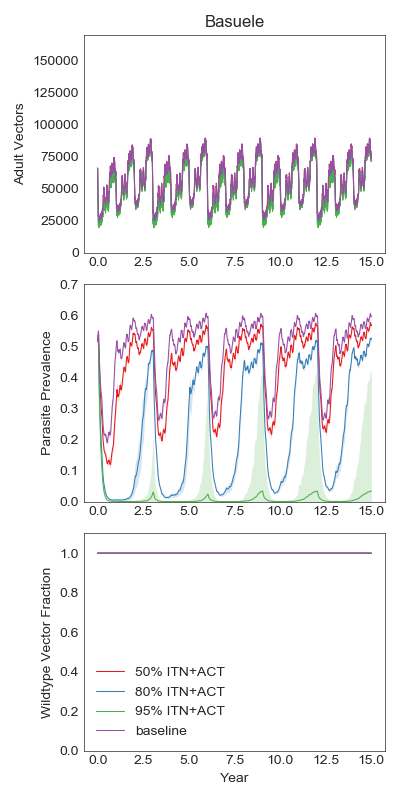
**

D) Gene drives alone vs Baseline E) 80%ITN+ACT+gene drives vs Baseline F) 95%ITN+ACT+gene drives vs Baseline


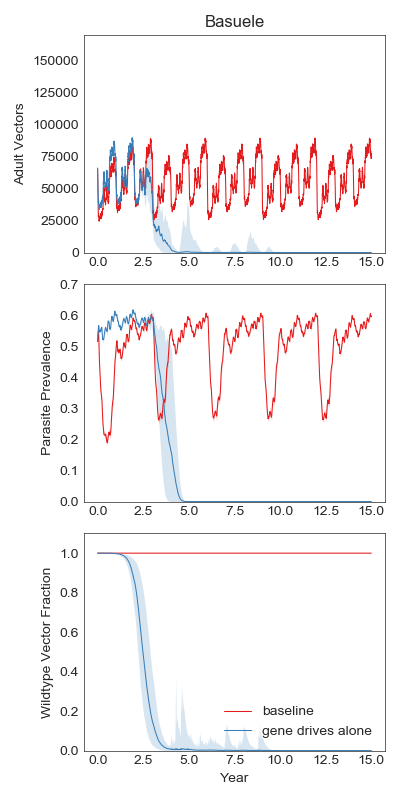

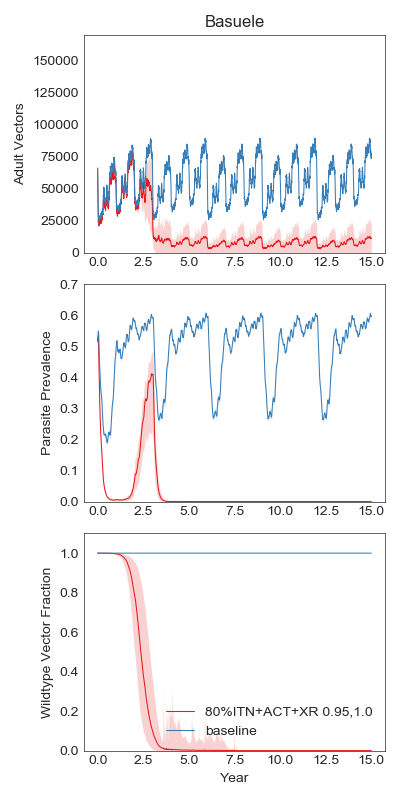

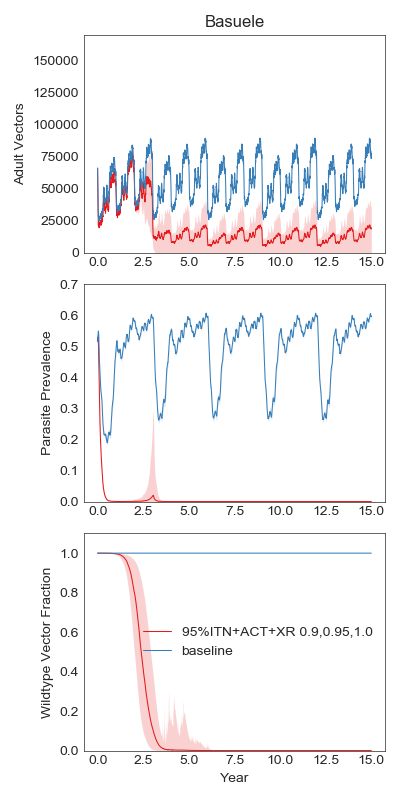


***Figure S5.2 Equateur***

A) ACT vs Baseline B) ITN vs Baseline C) ITN+ACT vs Baseline

| 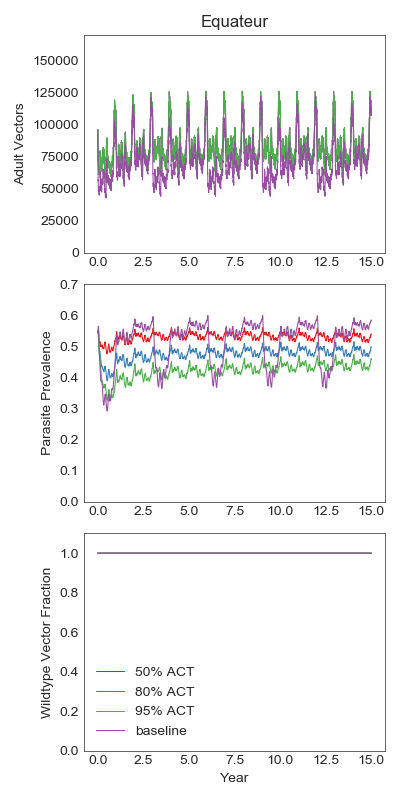 | 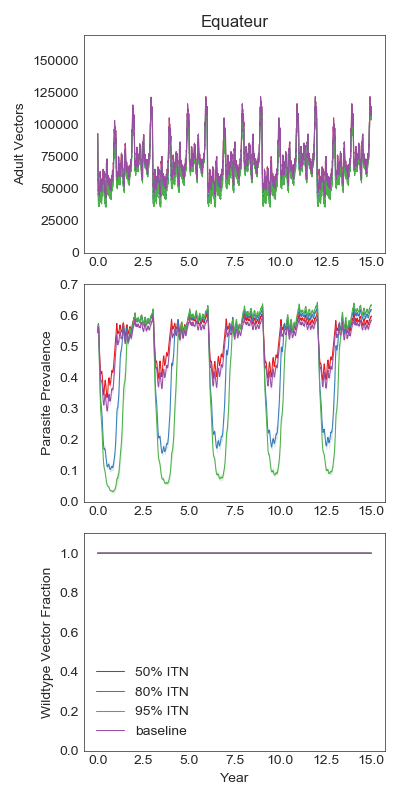 | 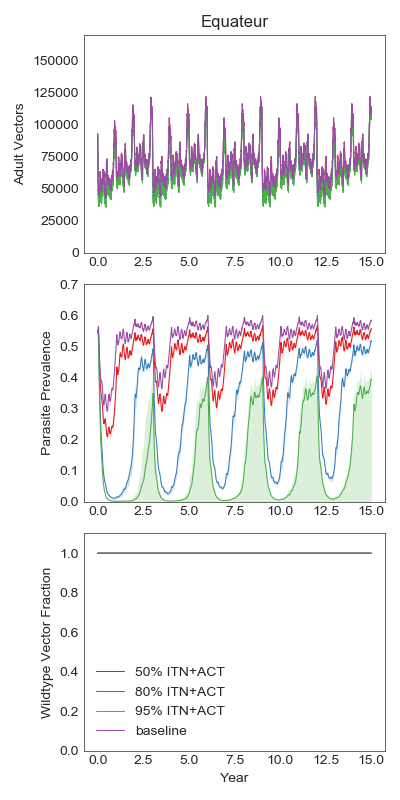 |
| --- | --- | --- |

D) Gene drives alone vs Baseline E) 80%ITN+ACT+gene drives vs Baseline F) 95%ITN+ACT+gene drives vs Baseline

| 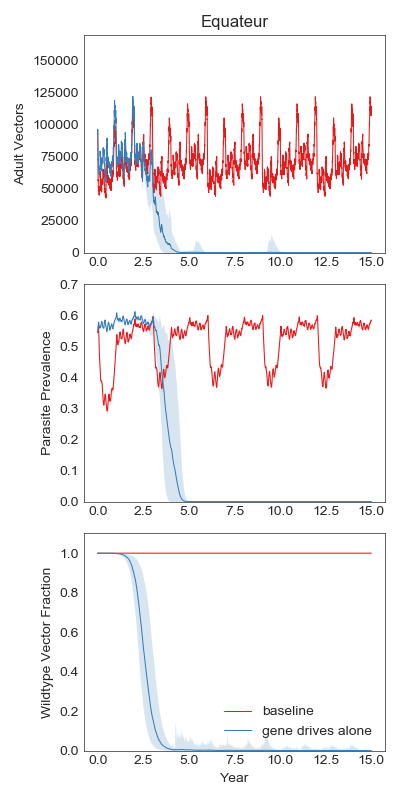 | 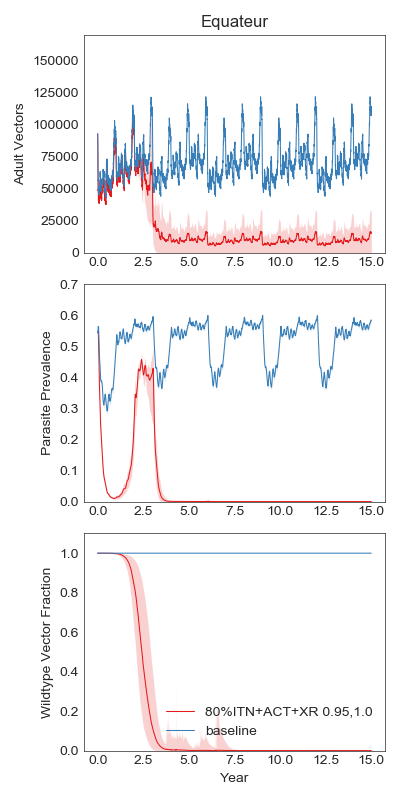 | 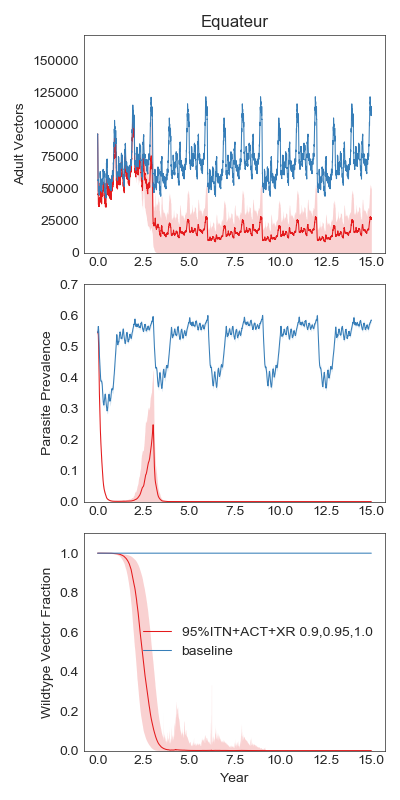 |
| --- | --- | --- |

***Figure S5.3 Haut Katanga***

A) ACT vs Baseline B) ITN vs Baseline C) ITN+ACT vs Baseline

| 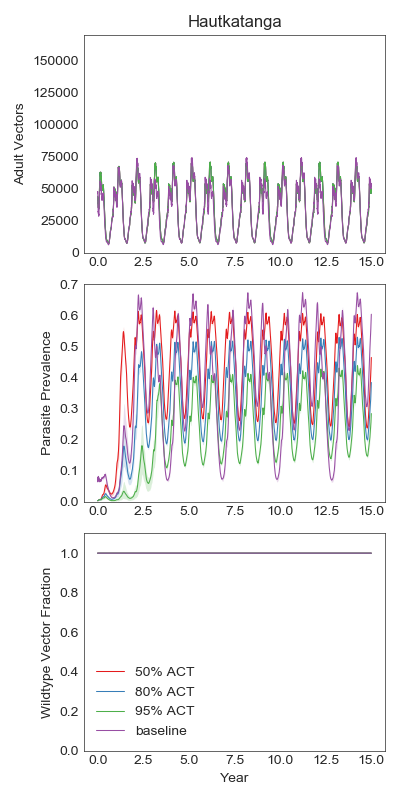 | 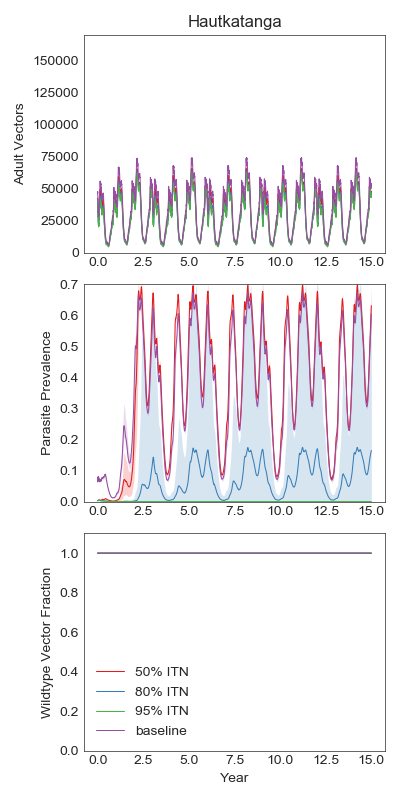 | 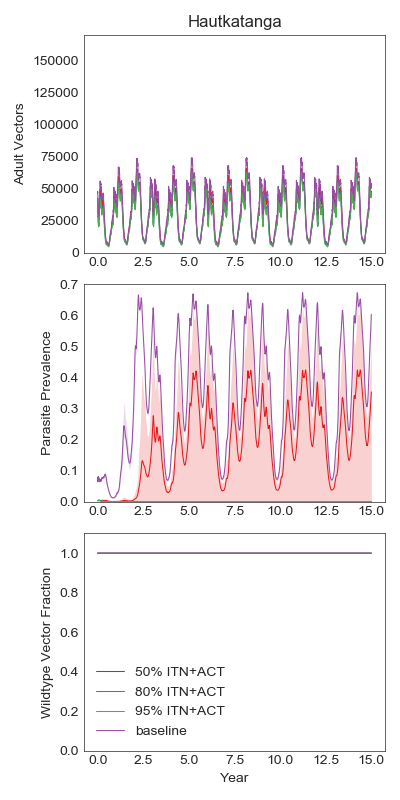 |
| --- | --- | --- |

D) Gene drives alone vs Baseline E) 80%ITN+ACT+gene drives vs Baseline F) 95%ITN+ACT+gene drives vs Baseline

| 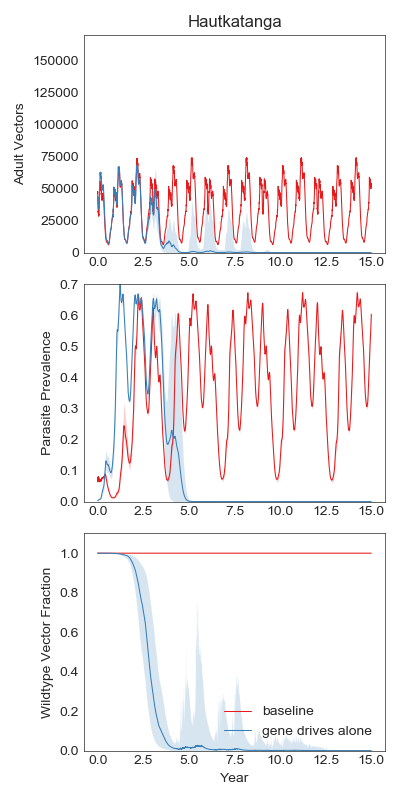 | 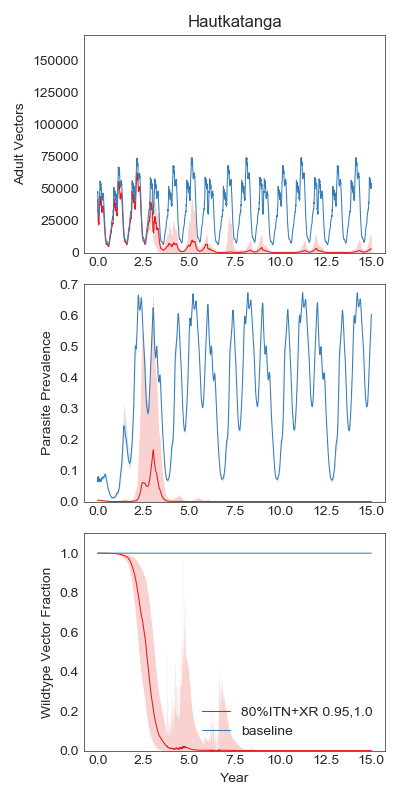 | 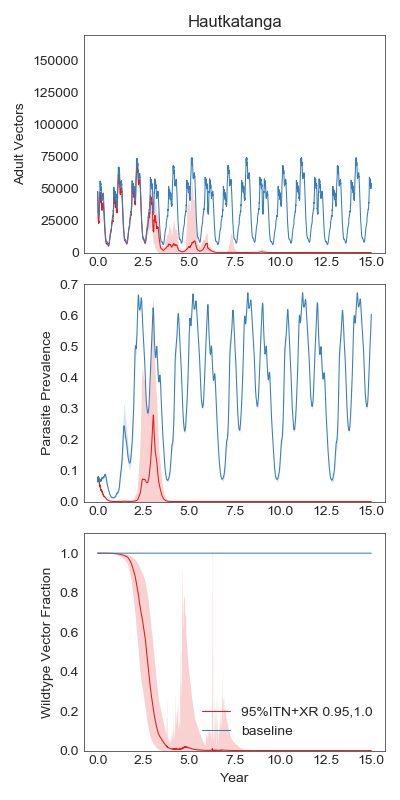 |
| --- | --- | --- |

G) 95%ITN+ACT+gene drives vs Baseline H) 50%ITN+ACT+gene drives vs Baseline

| 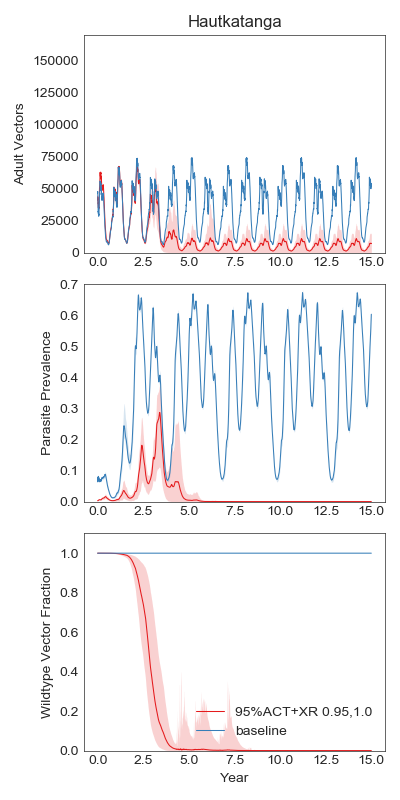 | 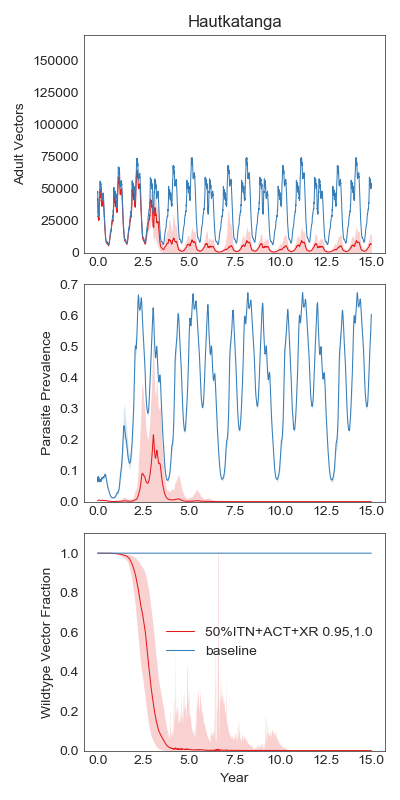 |  |
| --- | --- | --- |

***Figure S5.4 Kasai Central***

A) ACT vs Baseline B) ITN vs Baseline C) ITN+ACT vs Baseline

| 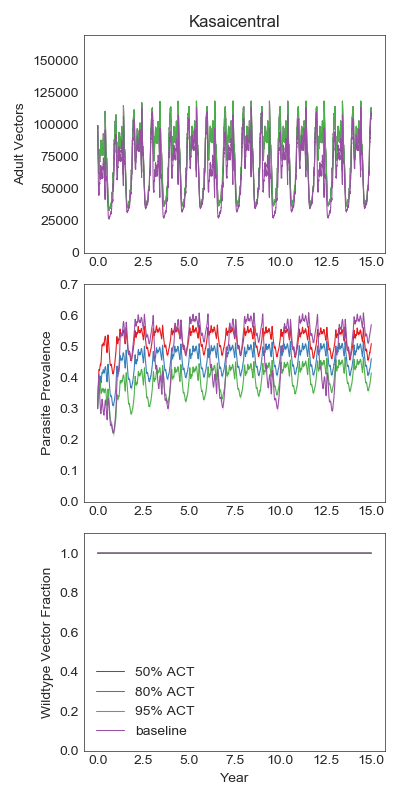 | 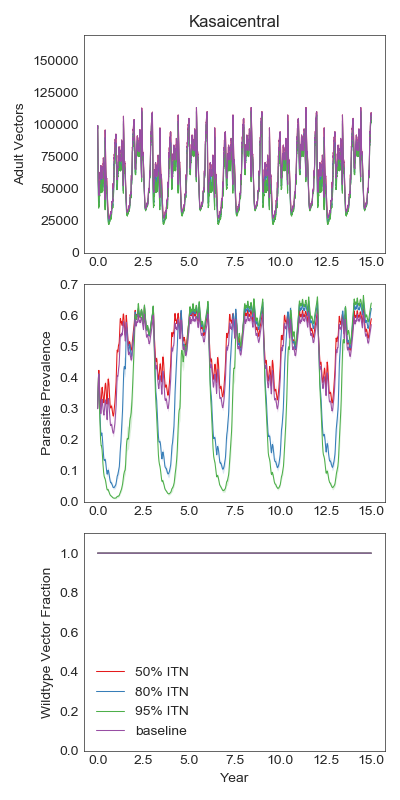 | 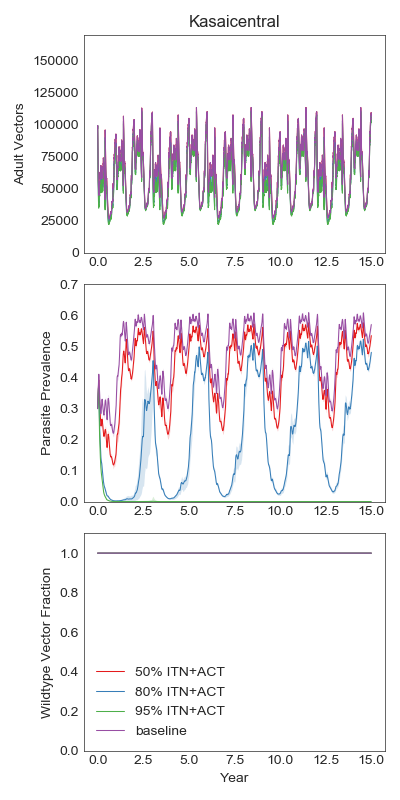 |
| --- | --- | --- |

D) Gene drives alone vs Baseline E) 95%ITN+ACT+gene drives vs Baseline F) 80%ITN+ACT+gene drives vs Baseline

| 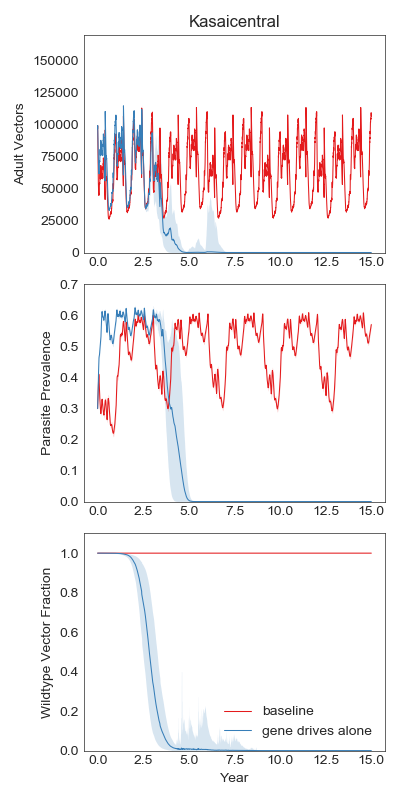 | 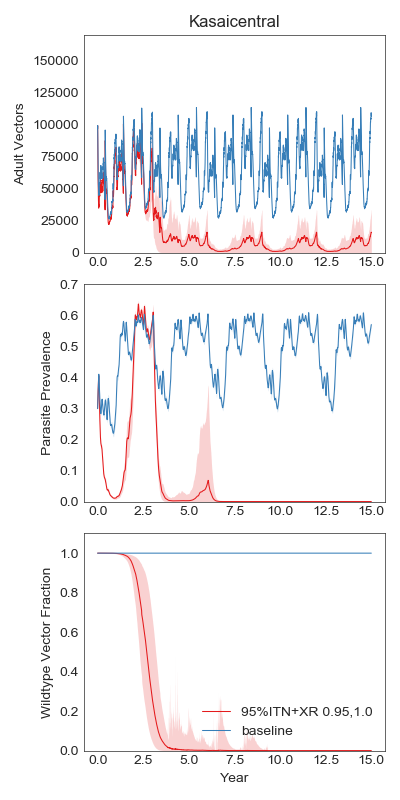 | 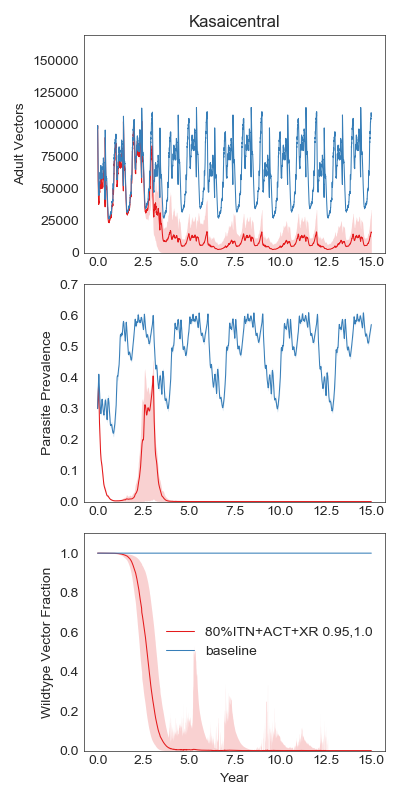 |
| --- | --- | --- |

***Figure S5.5 Kinshasa***

A) ACT vs Baseline B) ITN vs Baseline C) ITN+ACT vs Baseline

| 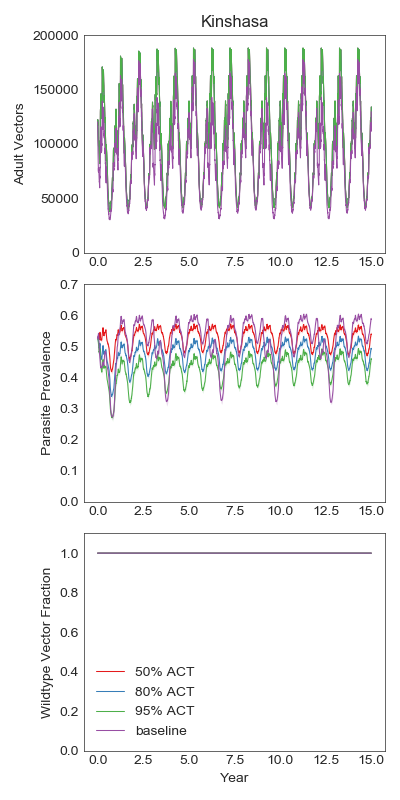 | 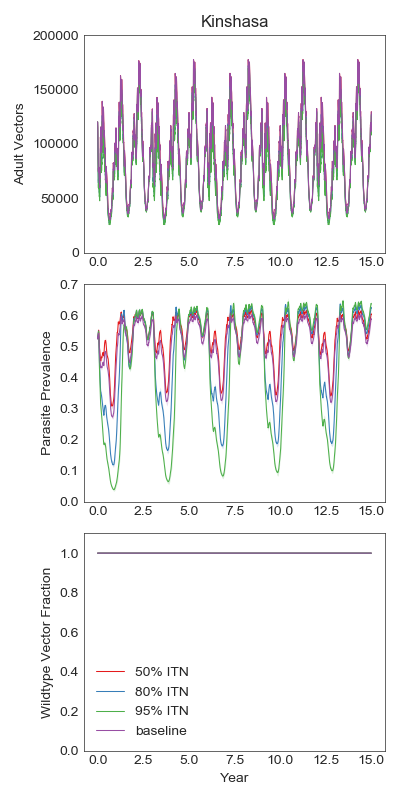 | 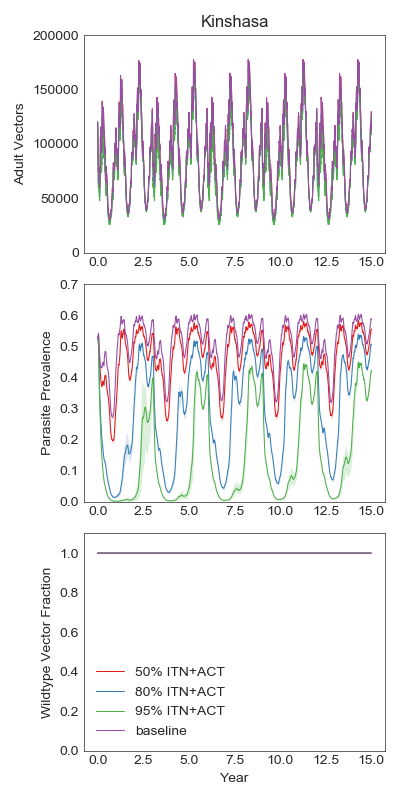 |
| --- | --- | --- |

D) Gene drives alone vs Baseline E) 80%ITN+ACT+gene drives vs Baseline F) 95%ITN+ACT+gene drives vs Baseline

| 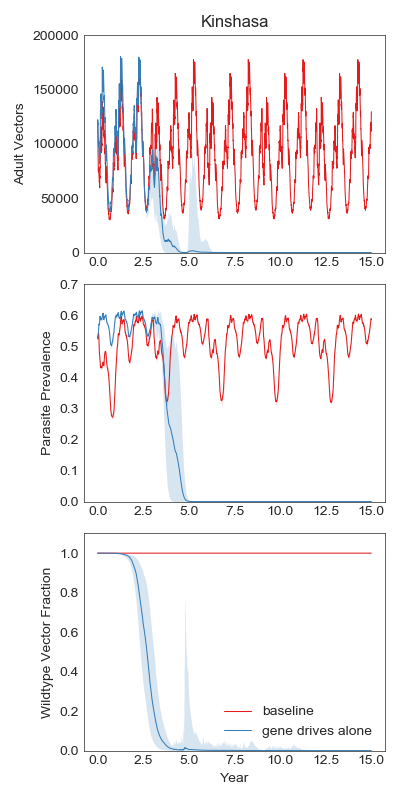 | 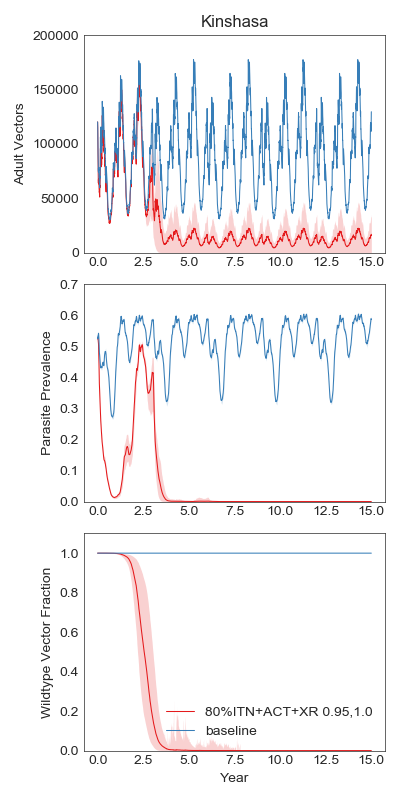 | 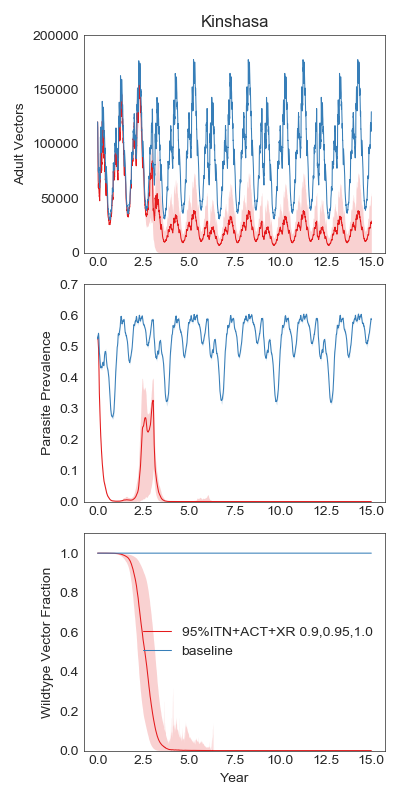 |
| --- | --- | --- |

***Figure S5.6 Kwango***

A) ACT vs Baseline B) ITN vs Baseline C) ITN+ACT vs Baseline

| 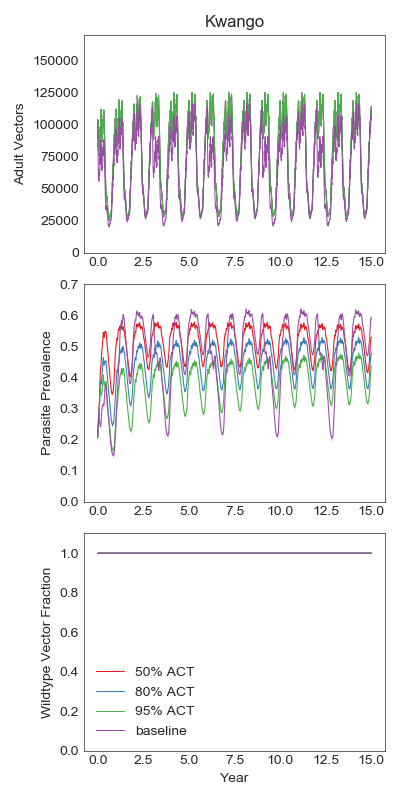 | 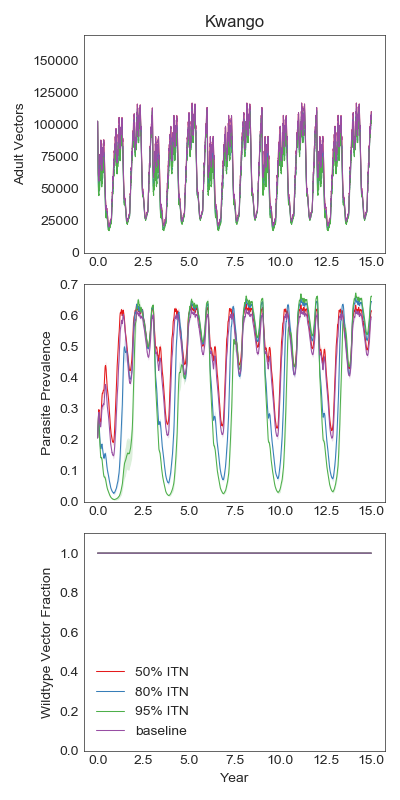 | 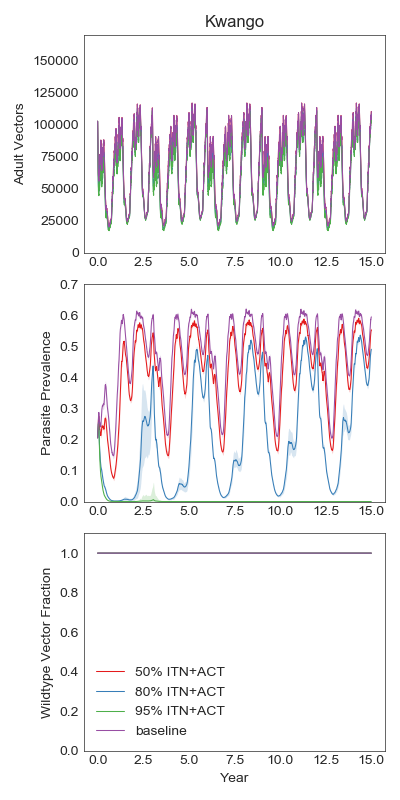 |
| --- | --- | --- |

D) Gene drives alone vs Baseline E) 95%ITN+ACT+gene drives vs Baseline F) 80%ITN+ACT+gene drives vs Baseline

| 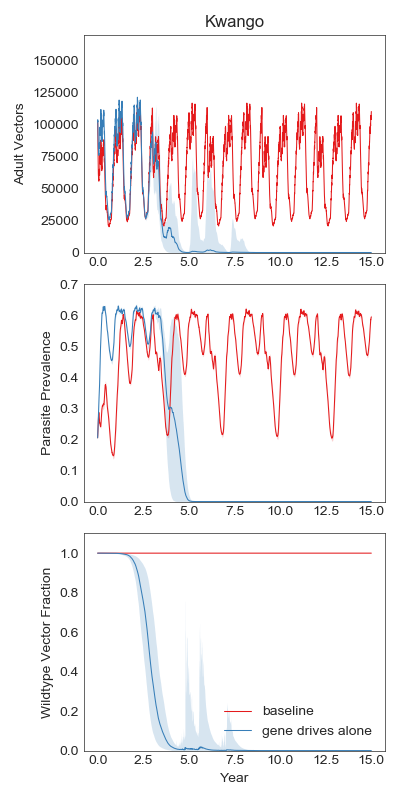 | 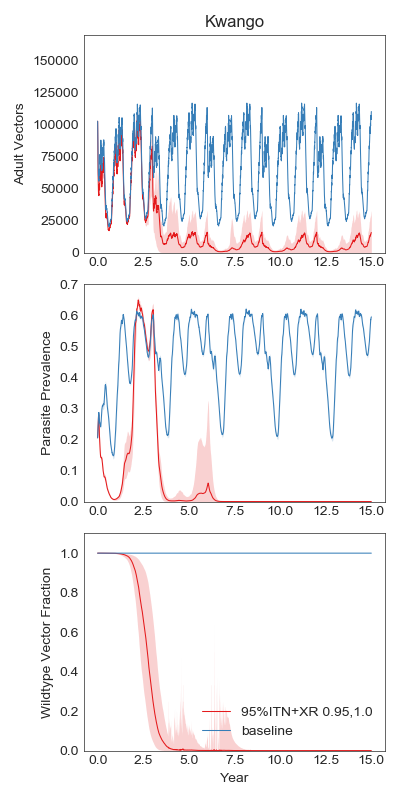 | 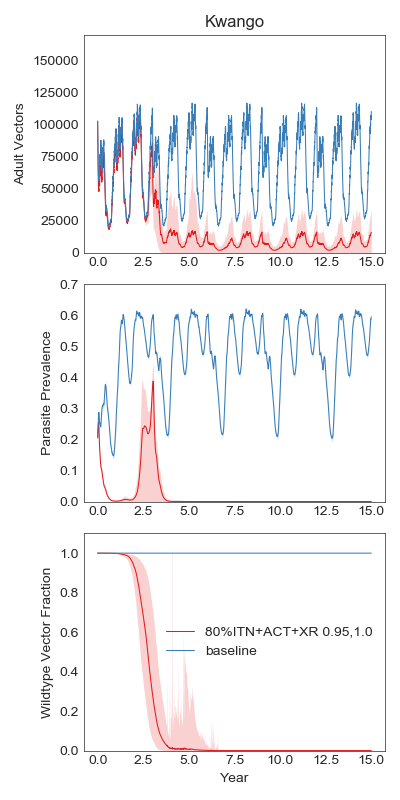 |
| --- | --- | --- |

***Figure S5.7 Nord Ubangui***

A) ACT vs Baseline B) ITN vs Baseline C) ITN+ACT vs Baseline

| 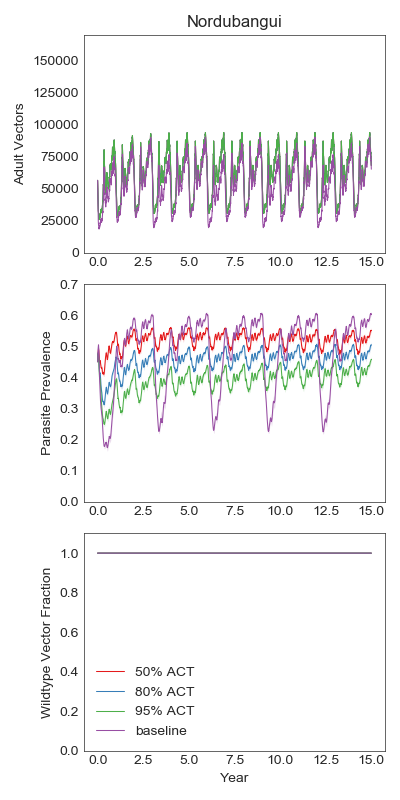 | 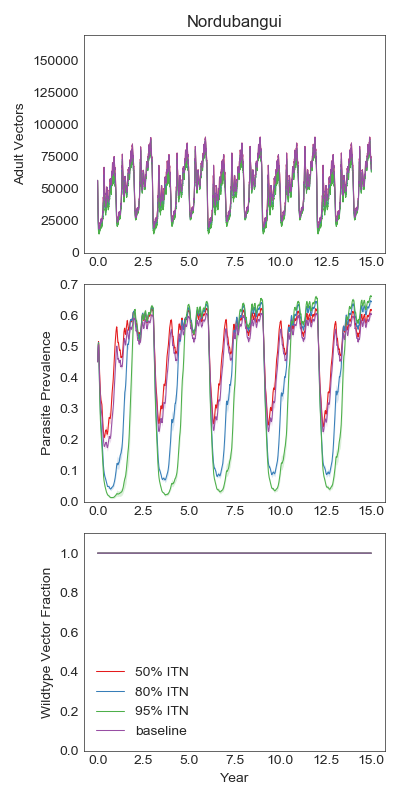 | 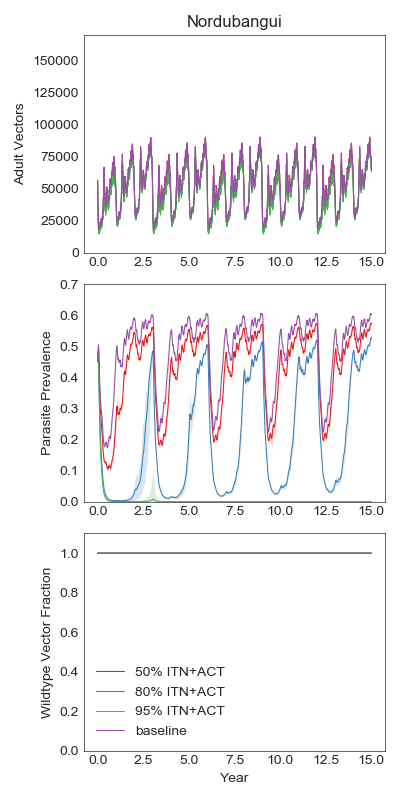 |
| --- | --- | --- |

D) Gene drives alone vs Baseline E) 95%ITN+ACT+gene drives vs Baseline F) 80%ITN+ACT+gene drives vs Baseline

| 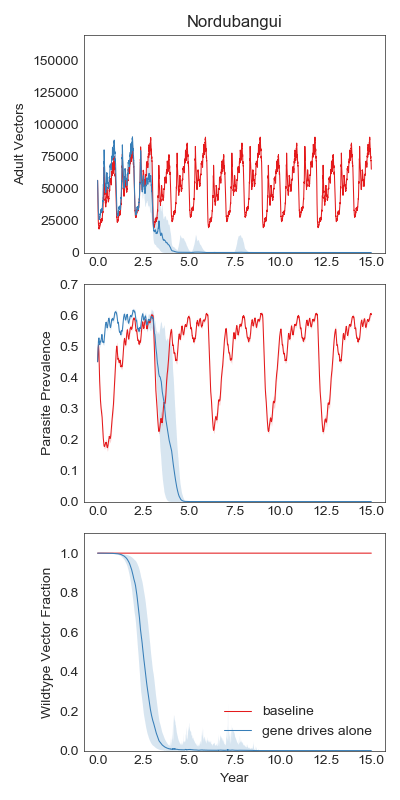 | 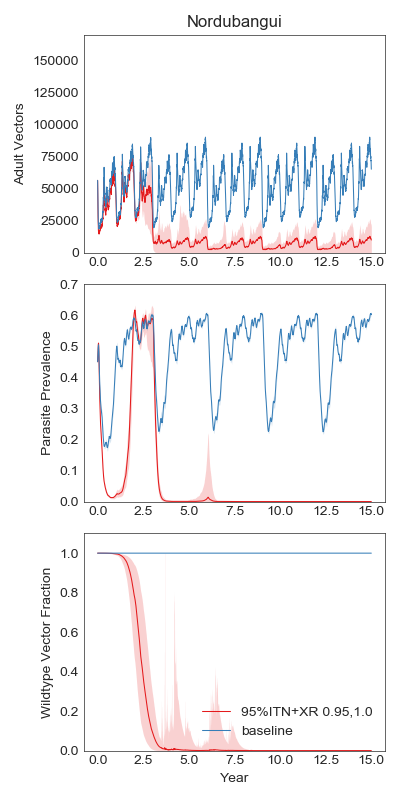 | 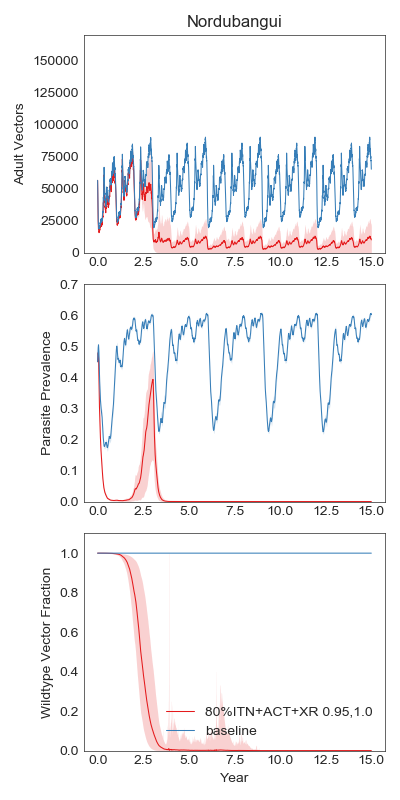 |
| --- | --- | --- |

Figure S5.8 Simulation outputs comparing post-release of 300 driving-Y gene drives in non-spatial and spatial setups of the study sites.

No other interventions are included and drive parameters are X-Shredding = 1.0, FR = 0.05 to 0.15. Shaded areas indicate 95% predicted intervals from 75 stochastic realizations.

|  | | | |  |
| --- | --- | --- | --- | --- |
| 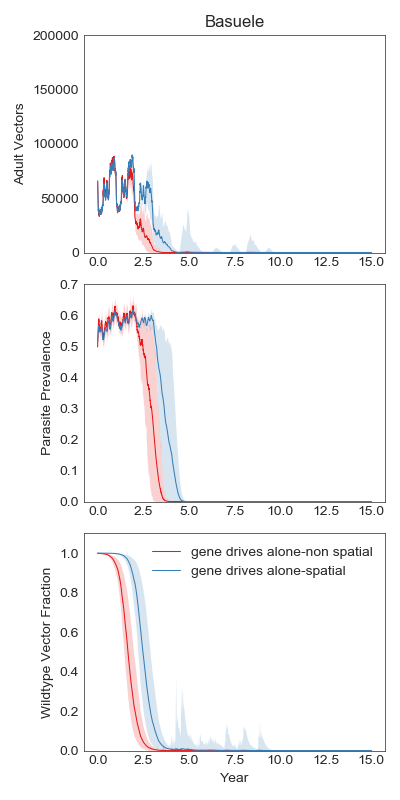 | 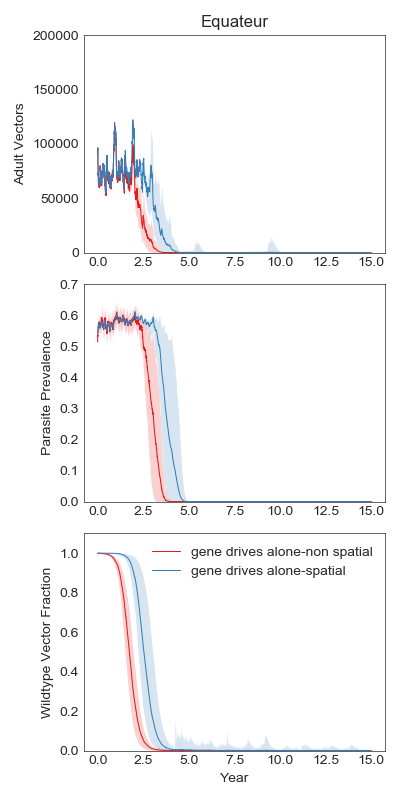 | 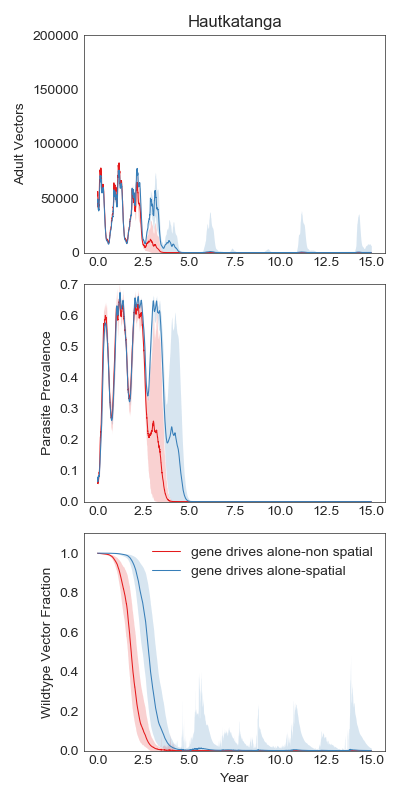 | 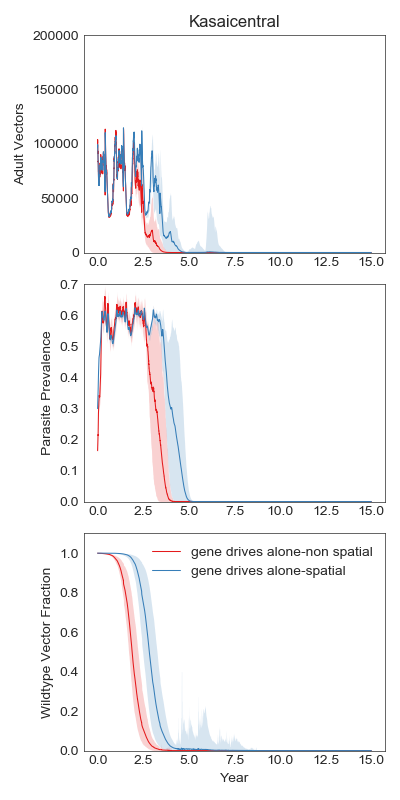 | |
| 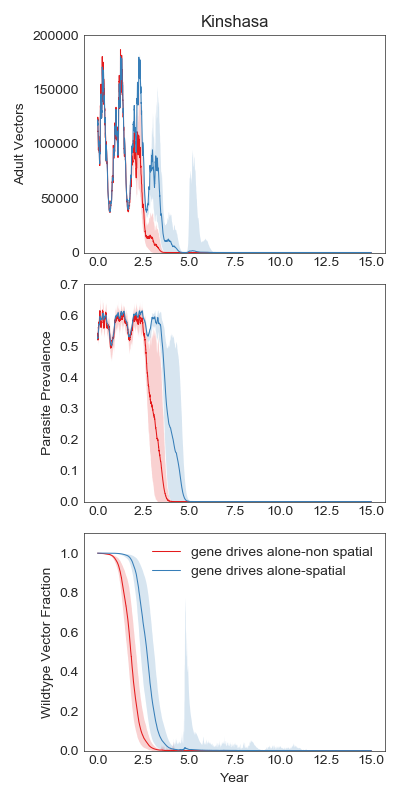 | 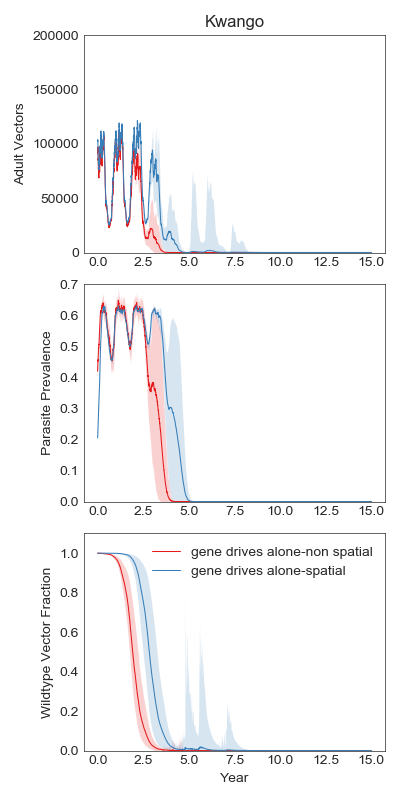 | 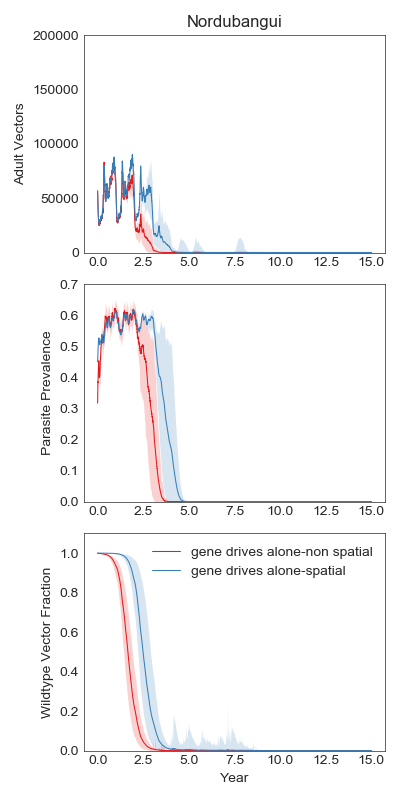 |  | |

# Supplementary 6: Sensitivity of results to vector and human migration

***Figure S6.1 Impact of reducing vector migration. Red line: results from main text. Scale factor (sf) is a uniform multiplier on vector migration rate.***

Kwango, Nord Ubangui, Bas Uele: 300 gene drives alone, X-shredding = 1.0

***
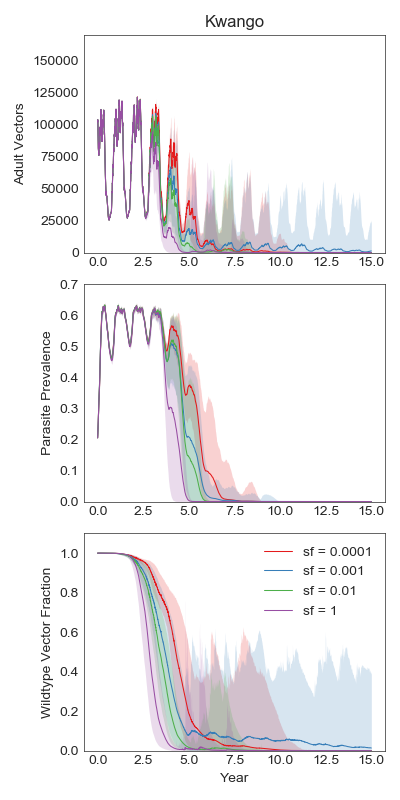

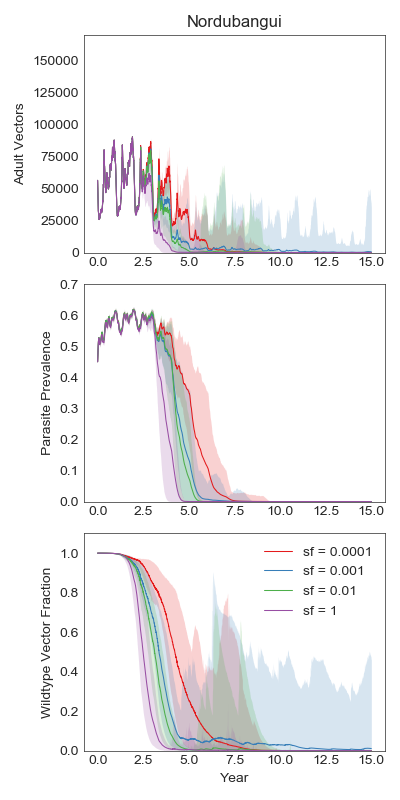
*
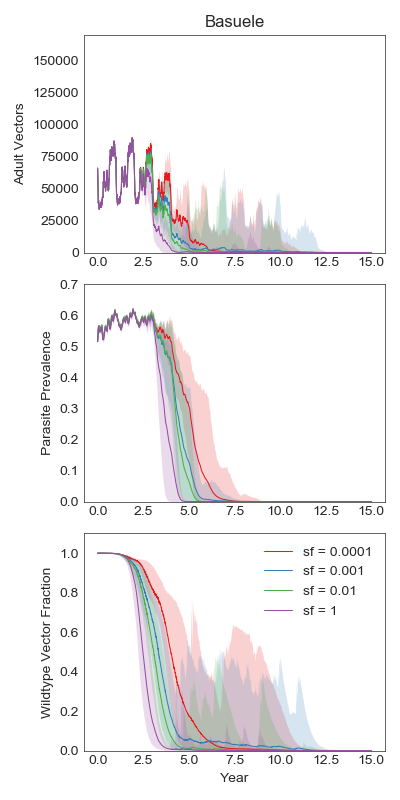
**

Haut Katanga: 95% ACT + 300 drives (X-shred = 1.0); Kasai Central: 50% ITN + 300 drives (X-shred = 0.95 and 1.0); Equateur: 80% ITN+ACT + 300 drives (X-shred = 1.0); Kinshasa: 95% ITN+ACT + 300 drives (X-shred = 0.9, 0.95, and 1.0)

**
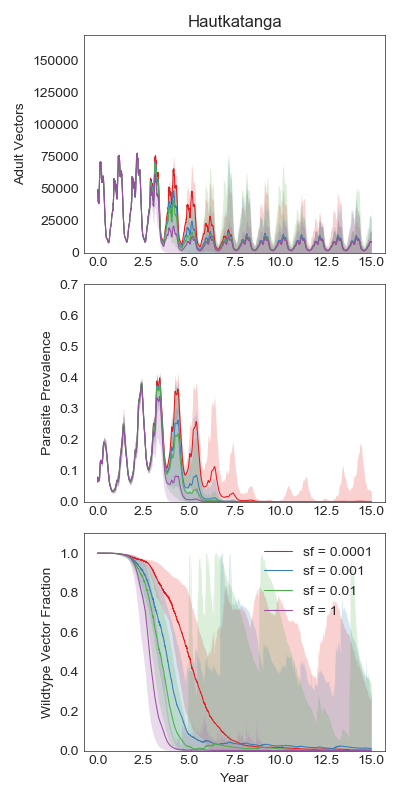

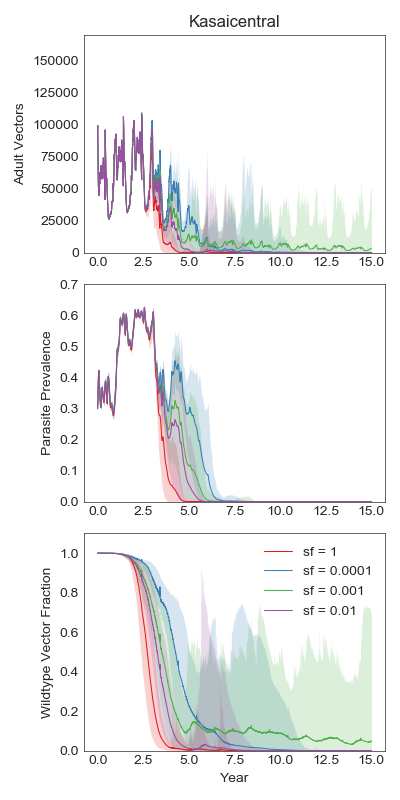

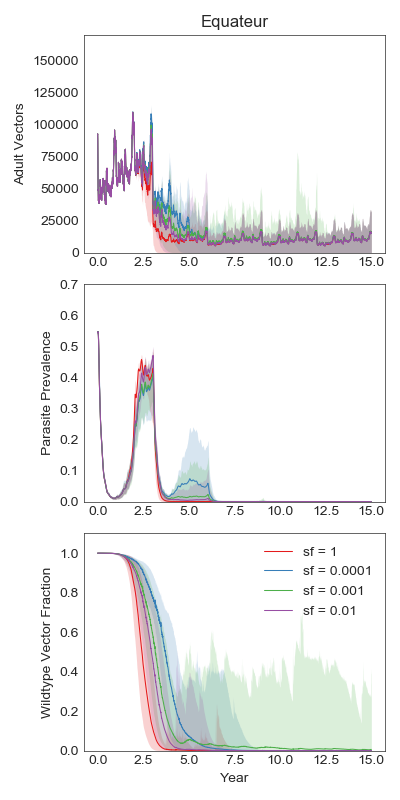

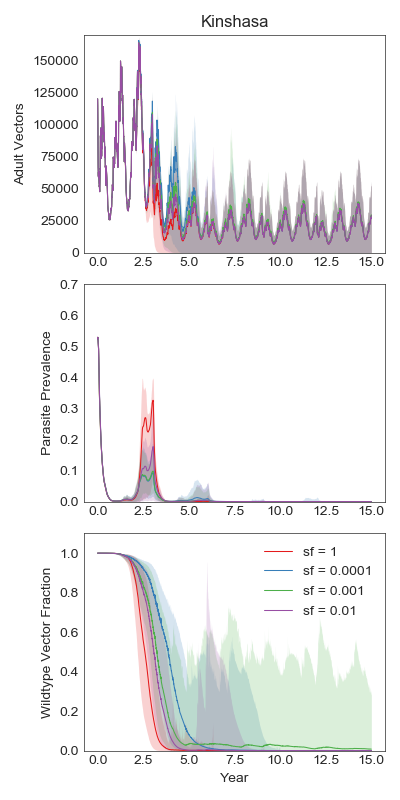
**

***Figure S6.2 Impact of including human migration***

Kwango, Nord Ubangui, Bas Uele: 300 gene drives alone, X-shredding = 1.0

***
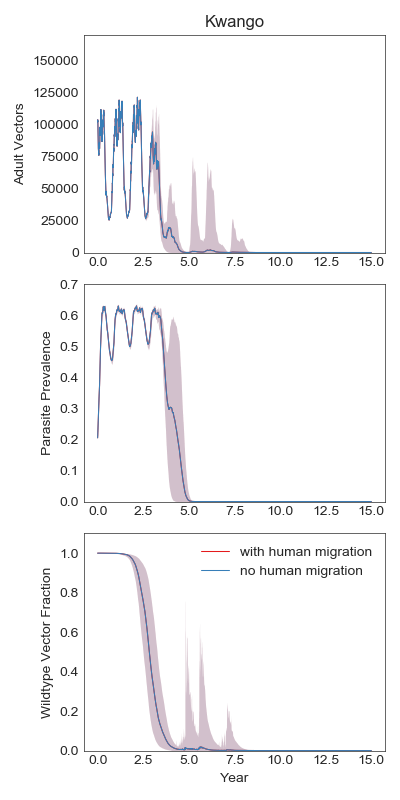

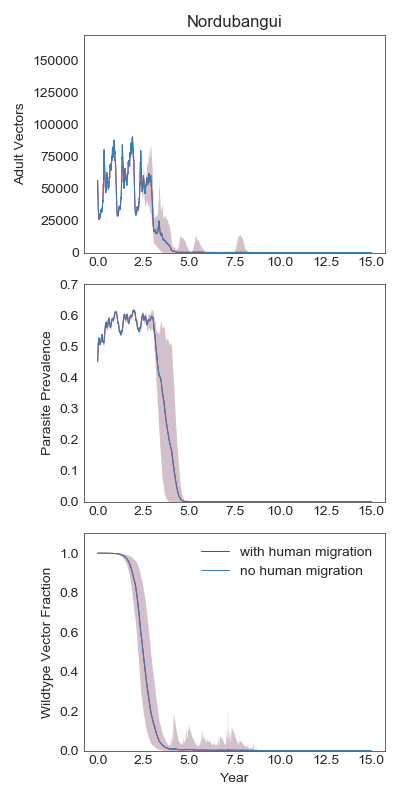
***
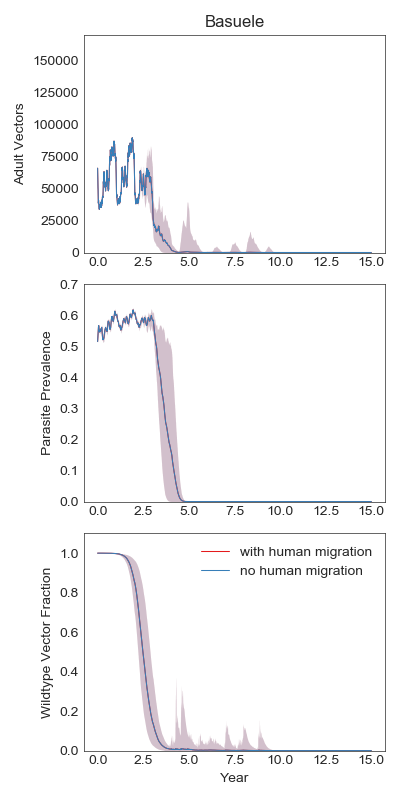


Haut Katanga: 95% ACT + 300 drives (X-shred = 1.0); Kasai Central: 50% ITN + 300 drives (X-shred = 0.95 and 1.0); Equateur: 80% ITN+ACT + 300 drives (X-shred = 1.0); Kinshasa: 95% ITN+ACT + 300 drives (X-shred = 0.9, 0.95, and 1.0)


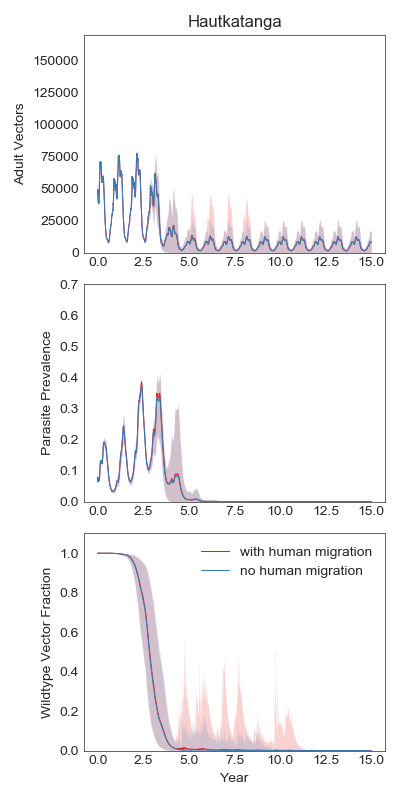

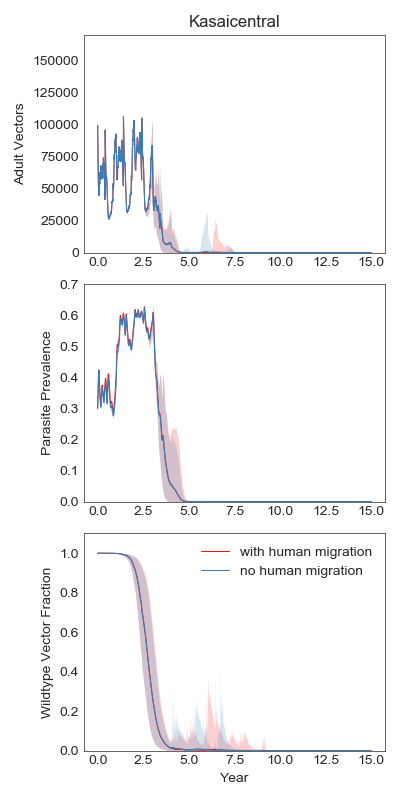

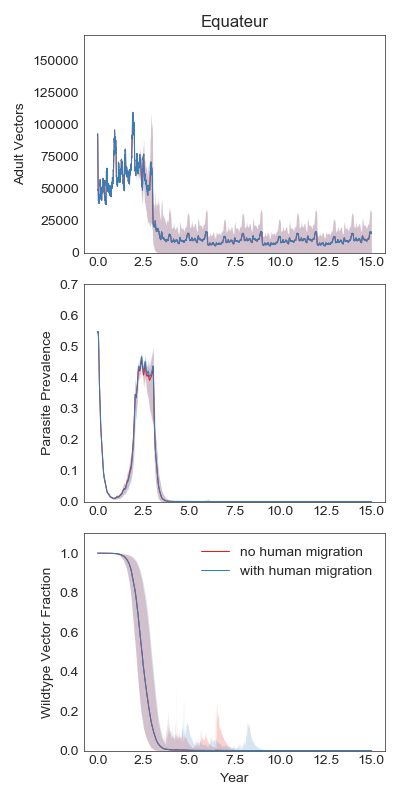

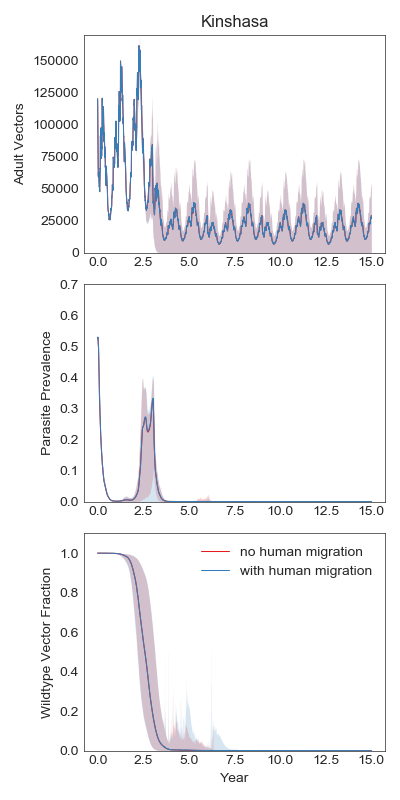


# Supplementary 7: Cost-effectiveness analysis

Table S7.1 Average DALYs averted per year per one million population across all locations estimated from model’s outputs in spatial simulation framework. Estimates of each scenario were compared with baseline scenario, which 50% ITNs and 19% ACT coverage were applied. For scenarios that included gene drives, only the estimates from scenarios that resulted in malaria elimination were included.

|  |  |  | **Average DALYs averted per year per one million population** | | | |
| --- | --- | --- | --- | --- | --- | --- |
|  |  |  | **Model’s estimates** | | | |
|  | **Intervention** | **Coverage** | **Average over 15 years** | **The first**  **interval:**  **year 1-5** | **The second**  **interval:**  **year 6-10** | **The last interval:**  **year 11-15** |
| Scenarios without  gene drives | ITNs | 50% | -3,696 | -6,818 | -2,361 | -1,910 |
|  |  | 80% | 1,482 | 12,827 | -1,990 | -6,390 |
|  |  | 95% | 8,727 | 27,165 | 2,158 | -3,143 |
|  | ACT | 50% | 1,680 | -7,733 | 2,506 | 10,266 |
|  |  | 80% | 12,962 | 10,390 | 10,212 | 18,283 |
|  |  | 95% | 21,437 | 25,261 | 15,883 | 23,169 |
|  | ITNs & ACT | 50% | 8,004 | 14,973 | 4,432 | 4,606 |
|  |  | 80% | 33,477 | 60,693 | 21,288 | 18,451 |
|  |  | 95% | 72,706 | 81,875 | 69,014 | 67,230 |
| Scenarios with gene drives | 300 gene drive mosquitoes with X-shredding rates = 1.0 alone | NA | 57,298 | 2,888 | 82,542 | 86,464 |
|  | ITNs plus gene drives with X-shredding rates = 0.95 and 1.0 | 80 | 57,561 | 12,201 | 81,580 | 78,904 |
|  | ITNs plus gene drives with X-shredding rates = 0.95 and 1.0 | 95 | 68,222 | 43,505 | 75,420 | 85,741 |
|  | ACT plus gene drives with X-shredding rates = 0.95 and 1.0 | 95 | 68,006 | 40,162 | 83,090 | 80,766 |
|  | ITNs & ACT plus gene drives with X-shredding rates = 0.95 and 1.0 | 50 | 67,740 | 39,311 | 83,142 | 80,766 |
|  | ITNs & ACT plus gene drives with X-shredding rates = 0.95 and 1.0 | 80 | 81,029 | 73,234 | 82,414 | 87,441 |
|  | ITNs & ACT plus gene drives with X-shredding rates = 0.9, 0.95 and 1.0 | 95 | 85,307 | 86,609 | 81,819 | 87,492 |

Notes for Table S7.1: 1) NA: Not applicable

2) Green highlight: the scenario achieved malaria elimination

3) It is possible that the DALY averted results turned out to be negative figures in some scenarios since the combination of ITNs at 50% coverage and ACT at 19% coverage was applied in the baseline scenarios that were used as a comparator to reflect reality. For example, a 50%ITNs scenario means only ITNs at 50% coverage was applied as a single intervention in the scenario. Therefore, it is understandable that the lower efficacy of 50%ITNs alone could be observed once compared to the comparator in which the combination of 50%ITNs and 19%ACT was applied.

Negative DALYs averted are in red texts.

4) WHO estimated the DALYs averted using null (do nothing) scenario as a comparator.

Table S7.2 Average cost per DALY averted of interventions and combinations applied estimated from model’s outputs.

|  |  | | | **Scenarios without gene drives** | | | | | | | | | |  | **Scenarios with gene drives** | | | | | | | | | | | | |
| --- | --- | --- | --- | --- | --- | --- | --- | --- | --- | --- | --- | --- | --- | --- | --- | --- | --- | --- | --- | --- | --- | --- | --- | --- | --- | --- | --- |
|  |  |  |  | **Intervention(s)** | | | | | | | | | | | | | | | | | | | | | | | |
|  |  |  |  | **ITNs** | | | **ACT** | | | **The combination of**  **ITNs and ACT** | | | |  | **300 gene-drive mosquitoes with X-shredding rates = 1.0 alone** | | **ITNs plus gene drives with X-shredding rates = 0.95 and 1.0** | | **ITNs plus gene drives with X-shredding rates = 0.95 and 1.0** | | **ACT plus gene drives with X-shredding rates = 0.95 and 1.0** | | **ITNs & ACT plus gene drives with X-shredding rates = 0.95 and 1.0** | | **ITNs & ACT plus gene drives with X-shredding rates = 0.95 and 1.0** | | **ITNs & ACT plus gene drives with X-shredding rates = 0.9, 0.95 and 1.0** |
|  |  |  |  | **Coverage (%)** | | | | | | | | | | | | | | | | | | | | | | | |
|  | **Parasite prevalence at the end of the 50-year run-in (%)** | **Coverage (%)** | **Interval** | **50** | **80** | **95** | **50** | **80** | **95** | **50** | **80** | **95** | **Bound** | | **NA** | **80** | | **95** | | **95** | | **50** | | **80** | | **95** | |
|  | **WHO’s estimates for Afr E**  **(4)** | | | 49 | 42 | 41 | 21 | 14 | 12 | 43 | 35 | 28 | NA | | NA | NA | | NA | | NA | | NA | | NA | | NA | |
|  | **7.27** | **Haut Katanga** | **Year**  **1-5** | -45 | 22 | 13 | -9 | 143 | 9 | 28 | 12 | 11 | **Lower bound** | | -36 | 111 | | 37 | | 23 | | 36 | | NA | | NA | |
|  |  |  |  |  |  |  |  |  |  |  |  |  | **Upper bound** | | -355 | 641 | | 205 | | 184 | | 200 | | NA | | NA | |
| **Estimates from model’s outputs** |  |  | **Year**  **6-10** | -238 | -91 | 23 | 43 | 18 | 12 | 303 | 10 | 9 | **Lower bound** | | 9 | 17 | | 26 | | 11 | | 17 | | NA | | NA | |
|  |  |  |  |  |  |  |  |  |  |  |  |  | **Upper bound** | | 96 | 142 | | 89 | | 95 | | 96 | | NA | | NA | |
|  |  |  | **Year**  **11-15** | -207 | -121 | 25 | 20 | 12 | 11 | 169 | 10 | 9 | **Lower bound** | | 9 | 17 | | 18 | | 12 | | 17 | | NA | | NA | |
|  |  |  |  |  |  |  |  |  |  |  |  |  | **Upper bound** | | 99 | 98 | | 91 | | 97 | | 99 | | NA | | NA | |
|  | **20.81** | **Kwango** | **Year**  **1-5** | -60 | 51 | 26 | -24 | 23 | 9 | 47 | 11 | 8 | **Lower bound** | | -340 | NA | | 32 | | NA | | NA | | 20 | | NA | |
|  |  |  |  |  |  |  |  |  |  |  |  |  | **Upper bound** | | -3,382 | NA | | 175 | | NA | | NA | | 101 | | NA | |
|  |  |  | **Year**  **6-10** | -427 | -494 | -156 | 55 | 17 | 12 | 112 | 59 | 9 | **Lower bound** | | 9 | NA | | 17 | | NA | | NA | | 18 | | NA | |
|  |  |  |  |  |  |  |  |  |  |  |  |  | **Upper bound** | | 85 | NA | | 95 | | NA | | NA | | 94 | | NA | |
|  |  |  | **Year**  **11-15** | -275 | -117 | -106 | 18 | 11 | 9 | 120 | 89 | 8 | **Lower bound** | | 8 | NA | | 16 | | NA | | NA | | 18 | | NA | |
|  |  |  |  |  |  |  |  |  |  |  |  |  | **Upper bound** | | 82 | NA | | 90 | | NA | | NA | | 91 | | NA | |
|  | **29.65** | **Kasai Central** | **Year**  **1-5** | -69 | 51 | 26 | -29 | 11 | 8 | 46 | 11 | 8 | **Lower bound** | | 11,661 | NA | | 32 | | NA | | NA | | 20 | | NA | |
|  |  |  |  |  |  |  |  |  |  |  |  |  | **Upper bound** | | 116,124 | NA | | 176 | | NA | | NA | | 103 | | NA | |
|  |  |  | **Year**  **6-10** | -175 | -245 | -161 | 64 | 20 | 14 | 157 | 68 | 9 | **Lower bound** | | 9 | NA | | 18 | | NA | | NA | | 19 | | NA | |
|  |  |  |  |  |  |  |  |  |  |  |  |  | **Upper bound** | | 87 | NA | | 97 | | NA | | NA | | 97 | | NA | |
|  |  |  | **Year**  **11-15** | -220 | -102 | -81 | 20 | 11 | 9 | 142 | 121 | 9 | **Lower bound** | | 8 | NA | | 16 | | NA | | NA | | 18 | | NA | |
|  |  |  |  |  |  |  |  |  |  |  |  |  | **Upper bound** | | 83 | NA | | 91 | | NA | | NA | | 92 | | NA | |
|  | **45.03** | **Nord Ubangui** | **Year**  **1-5** | -64 | 53 | 30 | -27 | 20 | 8 | 48 | 11 | 8 | **Lower bound** | | 82 | NA | | 31 | | NA | | NA | | 20 | | NA | |
|  |  |  |  |  |  |  |  |  |  |  |  |  | **Upper bound** | | 816 | NA | | 172 | | NA | | NA | | 102 | | NA | |
|  |  |  | **Year**  **6-10** | -168 | -925 | -206 | 125 | 22 | 15 | 138 | 66 | 9 | **Lower bound** | | 9 | NA | | 17 | | NA | | NA | | 19 | | NA | |
|  |  |  |  |  |  |  |  |  |  |  |  |  | **Upper bound** | | 88 | NA | | 96 | | NA | | NA | | 98 | | NA | |
|  |  |  | **Year**  **11-15** | -419 | -96 | -82 | 17 | 10 | 9 | 129 | 98 | 8 | **Lower bound** | | 8 | NA | | 16 | | NA | | NA | | 17 | | NA | |
|  |  |  |  |  |  |  |  |  |  |  |  |  | **Upper bound** | | 82 | NA | | 89 | | NA | | NA | | 91 | | NA | |
|  | **51.64** | **Bas Uele** | **Year**  **0-5** | -81 | 61 | 29 | -36 | 19 | 8 | 48 | 12 | 10 | **Lower bound** | | 65 | NA | | NA | | NA | | NA | | 20 | | 16 | |
|  |  |  |  |  |  |  |  |  |  |  |  |  | **Upper bound** | | 645 | NA | | NA | | NA | | NA | | 105 | | 87 | |
|  |  |  | **Year**  **6-10** | -181 | -606 | -322 | 117 | 22 | 15 | 140 | 170 | 9 | **Lower bound** | | 9 | NA | | NA | | NA | | NA | | 19 | | 18 | |
|  |  |  |  |  |  |  |  |  |  |  |  |  | **Upper bound** | | 87 | NA | | NA | | NA | | NA | | 97 | | 96 | |
|  |  |  | **Year**  **11-15** | -204 | -81 | -73 | 19 | 11 | 9 | 180 | 111 | 9 | **Lower bound** | | 8 | NA | | NA | | NA | | NA | | 18 | | 17 | |
|  |  |  |  |  |  |  |  |  |  |  |  |  | **Upper bound** | | 82 | NA | | NA | | NA | | NA | | 91 | | 90 | |
|  | **52.33** | **Kinshasa** | **Year**  **1-5** | -103 | 104 | 51 | -89 | 19 | 9 | 67 | 17 | 10 | **Lower bound** | | 77 | NA | | NA | | NA | | NA | | 25 | | 18 | |
|  |  |  |  |  |  |  |  |  |  |  |  |  | **Upper bound** | | 765 | NA | | NA | | NA | | NA | | 129 | | 97 | |
|  |  |  | **Year**  **6-10** | -170 | -366 | -1,602 | 110 | 20 | 13 | 181 | 69 | 23 | **Lower bound** | | 9 | NA | | NA | | NA | | NA | | 19 | | 18 | |
|  |  |  |  |  |  |  |  |  |  |  |  |  | **Upper bound** | | 88 | NA | | NA | | NA | | NA | | 98 | | 97 | |
|  |  |  | **Year**  **11-15** | -271 | -118 | -98 | 18 | 10 | 8 | 143 | 82 | 36 | **Lower bound** | | 8 | NA | | NA | | NA | | NA | | 17 | | 17 | |
|  |  |  |  |  |  |  |  |  |  |  |  |  | **Upper bound** | | 81 | NA | | NA | | NA | | NA | | 91 | | 90 | |
|  | **54.33** | **Equateur** | **Year**  **1-5** | -104 | 76 | 40 | -86 | 17 | 8 | 53 | 40 | 9 | **Lower bound** | | 55 | NA | | NA | | NA | | NA | | 23 | | 17 | |
|  |  |  |  |  |  |  |  |  |  |  |  |  | **Upper bound** | | 547 | NA | | NA | | NA | | NA | | 120 | | 91 | |
|  |  |  | **Year**  **6-10** | -180 | 5,457 | -706 | 115 | 21 | 13 | 143 | 75 | 19 | **Lower bound** | | 9 | NA | | NA | | NA | | NA | | 19 | | 18 | |
|  |  |  |  |  |  |  |  |  |  |  |  |  | **Upper bound** | | 88 | NA | | NA | | NA | | NA | | 98 | | 97 | |
|  |  |  | **Year**  **11-15** | -252 | -75 | -74 | 20 | 12 | 9 | 164 | 115 | 31 | **Lower bound** | | 8 | NA | | NA | | NA | | NA | | 18 | | 17 | |
|  |  |  |  |  |  |  |  |  |  |  |  |  | **Upper bound** | | 83 | NA | | NA | | NA | | NA | | 92 | | 91 | |

Note for Table S7.2: 1) NA: Not applicable

2) The scenarios that could achieve malaria elimination when adding gene drives were highlighted in green.

3) $int: International Dollars

4) upper bound: upper bound price, lower bound: lower bound price

Table S7.3 Summary annual costs and effectiveness by scenario number in Figure 4.

|  |  |  |  | **Year 1-5** | | | | **Year 6-10** | | | | **Year 11-15** | | | |  | |
| --- | --- | --- | --- | --- | --- | --- | --- | --- | --- | --- | --- | --- | --- | --- | --- | --- | --- |
| **Intervention** | **Coverage** | **Elimination** | **Label** | **Average yearly costs, $int** | | **Yearly effectiveness  (DALYs averted)** | | **Average yearly costs, $int** | | **Yearly effectiveness  (DALYs averted)** | | **Average yearly costs, $int** | | **Yearly effectiveness  (DALYs averted)** | |  | |
| ITN | 50% | No | A | 11,891 | | **-6,818** | | 11,885 | | **-2,361** | | 11,881 | | **-1,910** | |  | |
|  | 80% | No | B | 15,766 | | 12,827 | | 15,758 | | **-1,990** | | 15,756 | | **-6,390** | |  | |
|  | 95% | No | C | 17,711 | | 27,165 | | 17,704 | | 2,158 | | 17,696 | | **-3,143** | |  | |
| ACT | 50% | No | D | 4,823 | | **-7,733** | | 4,820 | | 2,506 | | 4,817 | | 10,266 | |  | |
|  | 80% | No | E | 5,091 | | 10,390 | | 5,090 | | 10,212 | | 5,089 | | 18,283 | |  | |
|  | 95% | No | F | 5,299 | | 25,261 | | 5,296 | | 15,883 | | 5,294 | | 23,169 | |  | |
| ITN & ACT | 50% | No | G | 16,953 | | 14,973 | | 16,947 | | 4,432 | | 16,941 | | 4,606 | |  | |
|  | 80% | Yes/No  (Yes in low transmission intensity) | H | 20,529 | | 60,693 | | 20,521 | | 21,288 | | 20,511 | | 18,451 | |  | |
|  | 95% | Yes/No  (Yes in low and medium transmission intensity) | I | 18,494 | | 81,875 | | 18,484 | | 69,014 | | 18,478 | | 67,230 | |  | |
| 300 gene drive mosquitoes with X-shredding rates = 1.0 alone | NA | Yes | J | 18,061 | 179,859 | 2,888 | 2,888 | 18,054 | 179,787 | 82,542 | 82,542 | 18,051 | 179,757 | 86,464 | 86,464 |  |  |
| ITNs plus gene drives with X-shredding rates = 0.95 and 1.0 | 80 | Yes | K | 33,893 | 196,004 | 12,201 | 12,201 | 33,874 | 195,897 | 81,580 | 81,580 | 33,840 | 195,700 | 78,904 | 78,904 |  |  |
| ITNs plus gene drives with X-shredding rates = 0.95 and 1.0 | 95 | Yes | L | 35,826 | 197,880 | 43,505 | 43,505 | 35,805 | 197,767 | 75,420 | 75,420 | 35,776 | 197,607 | 85,741 | 85,741 |  |  |
| ACT plus gene drives with X-shredding rates = 0.95 and 1.0 | 95 | Yes | M | 23,412 | 185,562 | 40,162 | 40,162 | 23,392 | 185,404 | 83,090 | 83,090 | 23,375 | 185,266 | 80,766 | 80,766 |  |  |
| ITNs & ACT plus gene drives with X-shredding rates = 0.95 and 1.0 | 50 | Yes | N | 35,084 | 197,217 | 39,311 | 39,311 | 35,069 | 197,127 | 83,142 | 83,142 | 35,049 | 197,015 | 80,766 | 80,766 |  |  |
| ITNs & ACT plus gene drives with X-shredding rates = 0.95 and 1.0 | 80 | Yes | O | 38,579 | 200,297 | 73,234 | 73,234 | 38,569 | 200,242 | 82,414 | 82,414 | 38,561 | 200,205 | 87,441 | 87,441 |  |  |
| ITNs & ACT plus gene drives with X-shredding rates = 0.9, 0.95 and 1.0 | 95 | Yes | P | 36,480 | 197,936 | 86,609 | 86,609 | 36,482 | 197,947 | 81,819 | 81,819 | 36,478 | 197,923 | 87,492 | 87,492 |  |  |
|  |  |  |  | Lower | Upper | Lower | Upper | Lower | Upper | Lower | Upper | Lower | Upper | Lower | Upper | Price |  |

Table S7.4 Incremental cost-effectiveness ratio after applying intervention(s)

|  |  | | | **ICER**  **($int per DALY averted)** | | | | | |
| --- | --- | --- | --- | --- | --- | --- | --- | --- | --- |
|  |  |  |  | **The first interval:**  **year 1-5** | | **The second interval:**  **year 6-10** | | **The last interval:**  **year 11-15** | |
|  | **Intervention** | **Coverage** | **Label** | **Lower bound** | **Upper bound** | **Lower bound** | **Upper bound** | **Lower bound** | **Upper bound** |
| Scenarios without gene drives | ITNs | 50% | A | dominated | dominated | negative | dominated | negative | dominated |
|  |  | 80% | B | dominated | dominated | negative | dominated | negative | dominated |
|  |  | 95% | C | 6.52 | 6.52 | negative | dominated | negative | dominated |
|  | ACT | 50% | D | negative | negative | negative | negative | negative | negative |
|  |  | 80% | E | negative | negative | negative | negative | negative | negative |
|  |  | 95% | F | **First point** | **First point** | negative | **First point** | negative | **First point** |
|  | ITNs & ACT | 50% | G | dominated | dominated | negative | dominated | negative | dominated |
|  |  | 80% | H | 0.43 | 0.43 | dominated | 2.82 | dominated | dominated |
|  |  | 95% | I | 0.23 | 0.23 | dominated | 0.25 | dominated | 0.30 |
| Scenarios with gene drives | 300 gene drive mosquitoes with X-shredding rates = 1.0 alone | NA | J | dominated | dominated | **First point** | 2.62 | **First point** | 2.76 |
|  | ITNs plus gene drives with X-shredding rates = 0.95 and 1.0 | 80 | K | dominated | dominated | dominated | 2.90 | dominated | 3.42 |
|  | ITNs plus gene drives with X-shredding rates = 0.95 and 1.0 | 95 | L | 1.67 | 10.56 | dominated | 3.23 | dominated | 3.07 |
|  | ACT plus gene drives with X-shredding rates = 0.95 and 1.0 | 95 | M | 1.22 | 12.10 | 9.74 | 2.68 | dominated | 3.12 |
|  | ITNs & ACT plus gene drives with X-shredding rates = 0.95 and 1.0 | 50 | N | 2.12 | 13.66 | 28.36 | 2.85 | dominated | 3.33 |
|  | ITNs & ACT plus gene drives with X-shredding rates = 0.95 and 1.0 | 80 | O | 0.69 | 4.06 | dominated | 2.93 | 20.99 | 3.03 |
|  | ITNs & ACT plus gene drives with X-shredding rates = 0.9, 0.95 and 1.0 | 95 | P | 0.51 | 3.14 | dominated | 2.92 | 17.93 | 2.99 |

**Keys for Table S7.4:**

- ICER: Incremental cost-effectiveness ratio
- Negative: the incremental cost and the incremental effect are negative
- Dominated: the incremental cost is positive, and the incremental effect is negative
- Vector control strategies that could reach malaria elimination were highlighted in green.
- **Incremental cost-effectiveness ratio (ICER)** is defined as the incremental change in cost, divided by the incremental change in its effectiveness. The first, second, and third points of each expansion path were highlighted in red, orange, and yellow accordingly.

Figure S7.4 Cost-effectiveness plane showing 16 intervention packages (10 individual and combination interventions at three assumed coverage levels) with 95% confidence interval and expansion paths for year 1-5, year 6-10, and year 11-15.

**
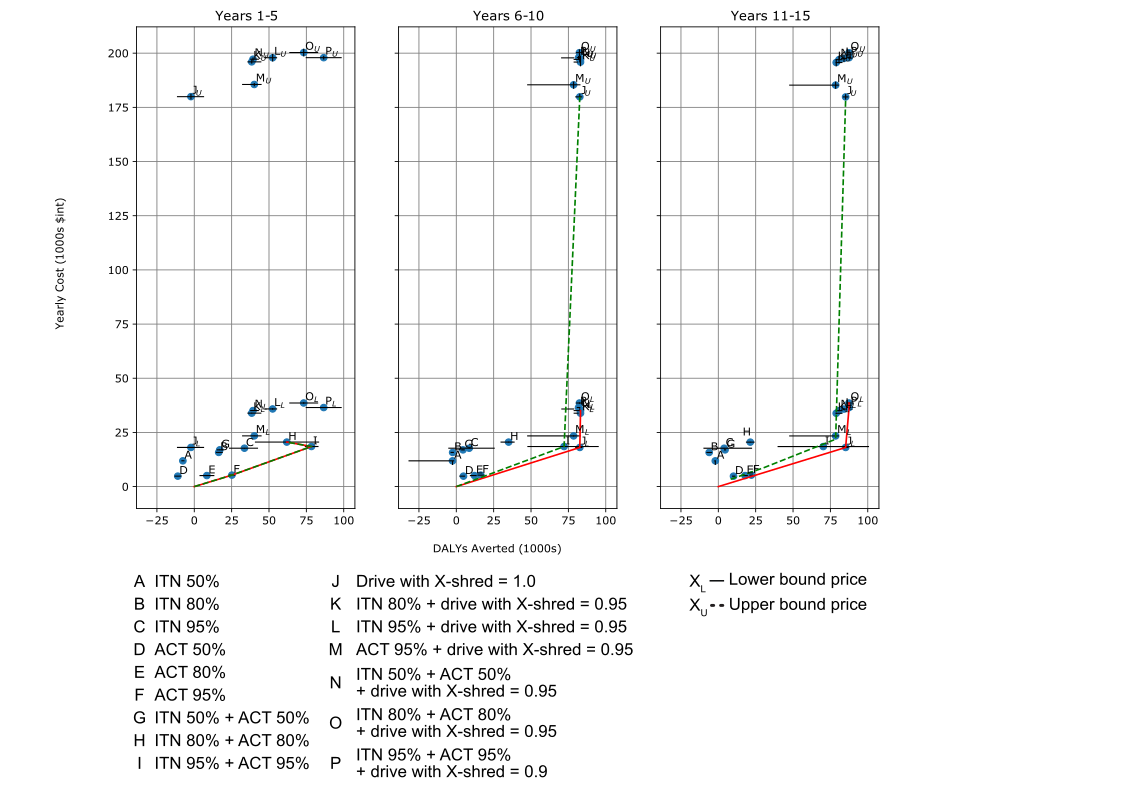
**

# Supplementary 8: Costs of vector control approaches that involve the release of mosquitoes to modify vector population – A systematic scoping review

Background

Vector control approaches that involve the release of mosquitoes to modify the vector population range from *Wolbachia* related technique to sterile insect production using radiation technique (1,2) . For *Wolbachia* related technique, the strategy proposed to infect mosquitoes with *Wolbachia* endosymbiotic that inhibits the viral replication and dissemination and eventually completely blocks vector-borne disease transmissions (3). The latter strategy, sterile insect technique (SIT) exposes male mosquitoes with low radiation in the laboratory production. This leads to sterilization of the male mosquitoes but maintain their copulation capacity (4). Once released to the environment, the sterile male mosquitoes mate wild female mosquitoes which would produce sterile eggs thereby eliminating the next generation progenies (5). Producing SIT insects at a large scale requires standardized mass-rearing procedures to produce good quality males that could compete with wild males to mate with females in the wild environment (6). Later development of these strategies includes the release of genetically engineered mosquitoes carrying dominant lethal allele that are capable of killing a subsequent generation (7). The produced OX513A mosquitoes are males that once mate with wild female mosquitoes will produce descendants that would not reach the adult stage due to lethal genes (8).

It is necessary for the decision to adapt these strategies specifically modifying mosquito vectors to be based on evidence of program effectiveness and cost-effectiveness of the interventions (9,10). However, evidence on costs and cost assessments of these strategies remain disparate. Data on costs could provide invaluable information for implementing vector control programs as malaria and other mosquito-borne infectious diseases continue to be a public health problem despite past and on-going control efforts (10). For sustenance of control efforts to achieve the disease elimination goal, it is important that the most cost-effective interventions are deployed (11). This supplementary is a systematic scoping review on costs and cost analysis of vector control approaches that involve the release of mosquitoes to modify the vector population focusing relevance on mosquito-borne diseases, especially malaria.

Methods

A systematic search of literatures in English language pertaining to costs of vector control methods to modify mosquito populations, published from 2010 to 2020, was performed (Diagram S1). Databases include National Center for Biotechnology Information (NCBI), Google Scholar, Crossref, and Scopus. Search terms include ‘Culicidae’ and ‘genetic engineering’ or ‘genetically modified’ and ‘costs’ or ‘cost analysis’ and ‘malaria’.

3,486 records of the initial search: 761 articles in PubMed Central, 13 articles in PubMed, 22 items in 13 books, 2480 articles in Google Scholar, 200 articles in Crossref, and 10 articles in Scopus, were screened for relevance, and those that included some forms of information on costs of new vector control intended to modify mosquito populations were assessed further in full texts for eligibility. 8 articles from reference lists thought to be relevant based on their titles alone were included in the full-text assessment. We excluded 3,428 articles that were duplicated, or full texts not provided. In full-text assessment, 66 articles were included based on selection criteria suggested costs or cost analysis of vector control methods intended to modify mosquito populations were described in monetary terms. 58 publications did not mention costs or mentioned but did not provide estimates in terms of monetary numbers were excluded. Details of literature selection procedures are in Diagram S1.

After eligible articles passed to the full-text assessment, we extracted details on publication year, country/region, target development, target disease, costs, the category of costs. Monetary cost data were first adjusted to US$ in the year of the initial study using historical exchange rates provided in the article if the adjustment was not supplied by the authors. If the currency year was not mentioned in the article, the publication date or date of article submission was used for the currency conversion. All monetary data were standardized to 2000 US$ using the US government consumer price index (CPI) data to adjust for inflation (12). All costs were adjusted to US$ value in year 2000 to allow comparison of the costs of interventions across data sources.

Diagram S8.1 Search strategy for costing studies

**
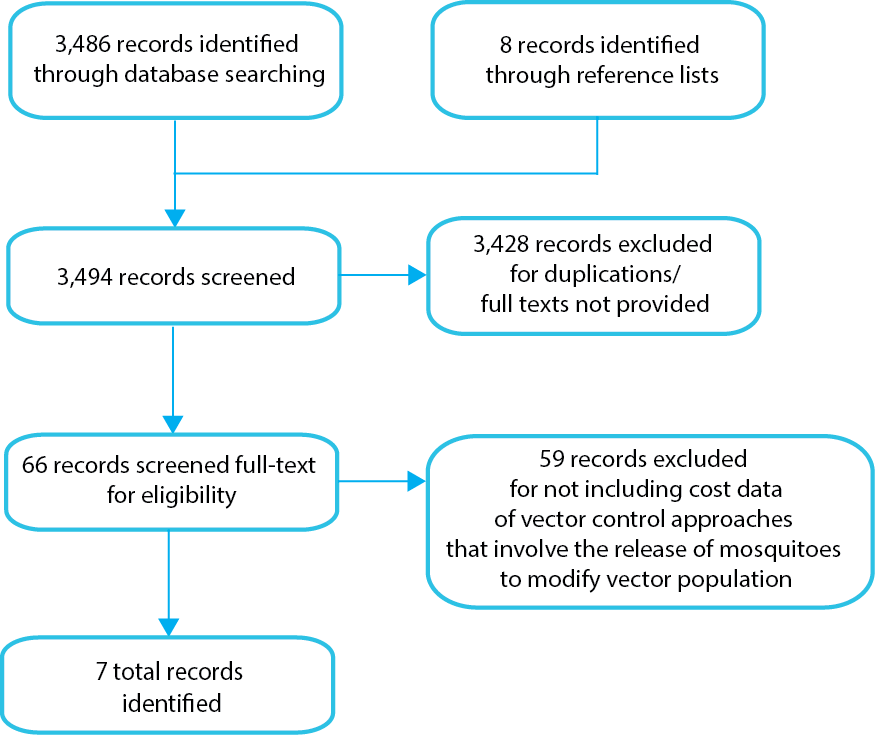
**

Results

Four out of seven eligible records focused on or used data from research conducted in low- and middle-income countries and were published between 2011 and 2018. Most studies focused on Dengue using various techniques including *Wolbachia* infected mosquitoes, sterile insect technique (SIT), Release of Insects carrying Dominant Lethals (RIDL), and Genetically engineered (GE) mosquitoes i.e., Oxitec. Only one study specifically identified cost of in malaria control. One study (13) developed a cost model to calculate costs of genetic RIDL technology for dengue and was referenced by another study (14). In terms of costs, costs of the release of SIT insects range from 29.42 to 800 US$ (14) per million insects depending on cost categories. The costs of GM mosquitoes are varied and higher at the beginning of release with lower cost during later maintenance phase. *Wolbachia* infected mosquitoes gives low cost at 0.72 US$ per person. Table S4 summarized the study characteristics and details on costs described in each study.

Table S8.2 Summary of study characteristics and details on costs of vector control approaches that involve the release of mosquitoes to modify the vector population focusing relevance on mosquito-borne diseases.

| **References** | **Publication year** | **Country** | **Target development** | **Target disease** | **Target species** | **Cost originally indicated** | **Converted cost to year 2000** | **Category of costs** |
| --- | --- | --- | --- | --- | --- | --- | --- | --- |
| Khamis D, El Mouden C, Kura K, et al. (14) | 2018 | Modelled based on multiple sources including other models, studies in South America, South Asia, and Africa | Sterile insect technique (SIT) mosquitoes | Malaria | Not specified | mean = 9.11 (min = 1.93, max = 25.36) US$ per 10,000 insect per day | 6.25 (1.32, 17.39) US$ per 10,000 insects per days | Operational costs |
| O’Neill SL, Ryan PA, Turley AP, Wilson G, Retzki K, Iturbe-Ormaetxe I et al.  (15) | 2018 | Australia | *Wolbachia* infected mosquitoes | Dengue and other *Aedes* transmitted arboviruses | *Aedes aegypti* | *Wolbachia* infected mosquito: 1.07 US$ per person | 0.72 US$ per person* | Deployment cost |
| Meghani Z and BoeÈte C.  (16) | 2018 | Brazil | Genetically engineered (GE) mosquitoes | Dengue | *Aedes aegypti* | 1.9 million US$ and 384,000 US$ for subsequent years per 50,000 urban population | 1.36 million US$ and 275,511.96 US$ for subsequent years per 50,000 urban population | Not specified but mentioned that the costs did not consider recurrent relicensing and subsidizing the cost |
|  |  |  |  |  |  | Oxitec: 10 US$ per person (in year 2016) | Oxitec: 7.17 US$ per person** |  |
| Alfaro-Murillo JA, Parpia AS, Fitzpatrick  MC, Tamagnan JA, Medlock J, Ndeffo-Mbah ML, et  al.  (17) | 2016 | Middle-income countries  (main country: Brazil) | GE mosquitoes (Oxitec) | Zika | *Aedes aegypti* (male) | 1.9 million US$ (in year 2015) in the first year and 384,000 US$ each year thereafter for an urban population of 50,000 | 1.38 million US$ (for 1^st^ year) and 278,987.58 US$ per year afterward | Not stated |
| Undurraga EA, Halasa YA, Shepard DS. (18) | 2016 | Brazil, Mexico, Panama, Puerto Rico, and Thailand | Genetic modified (GM) mosquitoes | Dengue | *Aedes* | 25-75 US$ per person per year in suppression phase and 10-20 US$ per person per year in maintenance phase, based on preliminary estimates by Oxitec. | 17.94-53.81 US$ per person per year in suppression phase and 7.17**-14.35 US$ per person per year in maintenance phase | Overall cost estimate |
| Bellini R, Medici A, Puggioli A, Balestrino F, Carrieri AM.  (19) | 2013 | India | SIT | Arboviral diseases in humans | Aedes albopictus  (Skuse) | 40 US$ per million male mosquitoes per day | 29.42 US$ per million male mosquitoes per day | Production cost |
| Alphey N, Alphey L, and Bonsall MB. (13) | 2011 | Modelled based on multiple sources including locations in Southeast Asia and South Asia | Genetic Release of Insects carrying Dominant Lethals (RIDL) technology (Release of Insects carrying a  Dominant Lethal) | Dengue | *Aedes aegypti* | 1 US$ per 1000 insects (in year 2008) | 0.80 US$ per 1,000 insects | Construction and operational costs |
|  |  |  |  |  |  | 2.30 US$ per case averted (in year 2008) | 1.84 US$ per case averted |  |
|  |  |  |  |  |  | Mean cost per person protected per year during the assessment period is 0.05–0.07 US$ or 0.52–0.68 US$ (release ratio 1 or 10,  respectively in year 2008) | Mean cost per person protected per year during the assessment period is 0.04-0.07 US$ or 0.42-0.54 US$  (release ratio 1 or 10 respectively) |  |

*lower bound cost applied in the main study.

**upper bound cost applied in the main study.

**Discussion**

During the past 10 years, only handful costing evidence of vector control approaches that involve the release of mosquitoes to modify the vector population focusing relevance on mosquito-borne diseases, especially malaria, has been made available. When costs were mentioned, the studies were often not undertaken alongside an evaluation of the clinical and epidemiological effect of the methods of interest. This systematic scoping review is an early attempt to combine evidence on costs of the approaches.

**References**

1. Committee on Gene Drive Research in Non-Human Organisms: Recommendations for Responsible Conduct; Board on Life Sciences; Division on Earth and Life Studies; National Academies of Sciences Engineering and Medicine. Gene Drives on the Horizon: Advancing Science, Navigating Uncertainty, and Aligning Research with Public Values [Internet]. National Academies Press; 2016. Available from: http://www.nap.edu/catalog/23405

2. Flores HA, O’Neill SL. Controlling vector-borne diseases by releasing modified mosquitoes [Internet]. Vol. 16, Nature Reviews Microbiology. Nature Publishing Group; 2018 [cited 2021 Jan 24]. p. 508–18. Available from: https://www.nature.com/articles/s41579-018-0025-0

3. Gomes FM, Barillas-Mury C. Infection of *Anopheline* mosquitoes with Wolbachia: Implications for malaria control [Internet]. Vol. 14, PLoS Pathogens. Public Library of Science; 2018 [cited 2021 Jan 24]. Available from: https://www.ncbi.nlm.nih.gov/pmc/articles/PMC6237385/

4. Reis NN, Silva AL da, Reis EPG, Silva FC e., Reis IGN. Viruses vector control proposal: genus Aedes emphasis. Vol. 21, Brazilian Journal of Infectious Diseases. Elsevier Editora Ltda; 2017. p. 457–63.

5. Reis NN, Silva AL da, Reis EPG, Silva FC e., Reis IGN. Viruses vector control proposal: genus Aedes emphasis. Brazilian Journal of Infectious Diseases [Internet]. 2017;21(4):457–63. Available from: http://dx.doi.org/10.1016/j.bjid.2017.03.020

6. Mamai W, Lobb LN, Bimbilé Somda NS, Maiga H, Yamada H, Lees RS, et al. Optimization of mass-rearing methods for *Anopheles arabiensis* larval stages: Effects of rearing water temperature and larval density on mosquito life-history traits. Journal of Economic Entomology. 2018;111(5):2383–90.

7. Hoang PN, Daniel Zucker J, Choisy M, Vinh HT. Causality analysis between climatic factors and dengue fever using the Granger causality. 2016 IEEE RIVF International Conference on Computing and Communication Technologies: Research, Innovation, and Vision for the Future, RIVF 2016 - Proceedings. 2016;49–54.

8. Wentzel M. Brasil testa duas ’ armas ’ contra o Aedes aegypti ; saiba quando teremos resultado O mosquito inoculado. BBC News Brasil. 2015;1–9.

9. WHO. Guidelines for malaria vector control [Internet]. 2019 [cited 2021 Jan 24]. Available from: https://apps.who.int/iris/bitstream/handle/10665/310862/9789241550499-eng.pdf?ua=1

10. WHO. Global Vector Control Response 2017-2030 [Internet]. 2017 [cited 2021 Jan 24]. Available from: https://apps.who.int/iris/bitstream/handle/10665/259205/9789241512978-eng.pdf;jsessionid=BCA2E1473557FBC791074D7675EA2BE3?sequence=1

11. White MT, Conteh L, Cibulskis R, Ghani AC. Costs and cost-effectiveness of malaria control interventions--a systematic review. Malaria journal. 2011 Nov;10:337.

12. U.S. Bureau of Labor Statistics. 2000 Home : The Economics Daily : U.S. Bureau of Labor Statistics [Internet]. 2000 [cited 2021 Jan 24]. Available from: https://www.bls.gov/opub/ted/2000/home.htm

13. Alphey N, Alphey L, Bonsall MB. A model framework to estimate impact and cost of genetics-based sterile insect methods for dengue vector control. PLoS ONE. 2011;6(10).

14. Khamis D, El Mouden C, Kura K, Bonsall MB. Optimal control of malaria: Combining vector interventions and drug therapies. Malaria Journal [Internet]. 2018;17(1):1–18. Available from: https://doi.org/10.1186/s12936-018-2321-6

15. O’Neill SL, Ryan PA, Turley AP, Wilson G, Retzki K, Iturbe-Ormaetxe I, et al. Scaled deployment of Wolbachia to protect the community from dengue and other aedes transmitted arboviruses. Gates Open Research. 2018 Aug 13;2:36.

16. Meghani Z, Boëte C. Genetically engineered mosquitoes, Zika and other arboviruses, community engagement, costs, and patents: Ethical issues. PLoS Neglected Tropical Diseases. 2018;12(7):1–7.

17. Alfaro-Murillo JA, Parpia AS, Fitzpatrick MC, Tamagnan JA, Medlock J, Ndeffo-Mbah ML, et al. A Cost-Effectiveness Tool for Informing Policies on Zika Virus Control. PLoS Neglected Tropical Diseases [Internet]. 2016;10(5):1–14. Available from: http://dx.doi.org/10.1371/journal.pntd.0004743

18. Undurraga EA, Halasa YA, Shepard DS. Economic Analysis of Genetically Modified Mosquito Strategies. In: Genetic Control of Malaria and Dengue. Elsevier Inc.; 2016. p. 375–408.

19. Bellini R, Medici A, Puggioli A, Balestrino F, Carrieri AM. Pilot Field Trials With Aedes albopictus Irradiated Sterile Males in Italian Urban Areas. J Med Entomol [Internet]. 2013 [cited 2021 Jan 24];50(2):317–25. Available from: http://dx.doi.org/10.1603/ME12048
